# Prospective and longitudinal natural history study of patients with Type 2 and 3 spinal muscular atrophy: Baseline data NatHis-SMA study

**DOI:** 10.1371/journal.pone.0201004

**Published:** 2018-07-26

**Authors:** Aurélie Chabanon, Andreea Mihaela Seferian, Aurore Daron, Yann Péréon, Claude Cances, Carole Vuillerot, Liesbeth De Waele, Jean-Marie Cuisset, Vincent Laugel, Ulrike Schara, Teresa Gidaro, Stéphanie Gilabert, Jean-Yves Hogrel, Pierre-Yves Baudin, Pierre Carlier, Emmanuel Fournier, Linda Pax Lowes, Nicole Hellbach, Timothy Seabrook, Elie Toledano, Mélanie Annoussamy, Laurent Servais, the NatHis-SMA study group

**Affiliations:** 1 Institute of Myology, GH Pitié Salpêtrière, Paris, France; 2 Centre de Référence des Maladies Neuromusculaires, CHU de Liège, Belgium; 3 Centre de Référence Maladies Neuromusculaires Atlantique-Occitanie-Caraïbes, Hôpital Hôtel-Dieu, Nantes, France; 4 Centre de Référence des Maladies Neuromusculaires, Hôpital des Enfants, Toulouse, France; 5 Unité de neurologie pédiatrique, Hôpital des Enfants, Toulouse, France; 6 Service de rééducation pédiatrique infantile”L’Escale”, Hôpital Mère Enfant, CHU-Lyon, Lyon, France; 7 Department of Pediatric Neurology, University Hospitals Leuven, Leuven, Belgium; 8 Department of Development and Regeneration, KU Leuven Kulak Kortijk, Kortrijk, Belgium; 9 Centre de Référence des Maladies Neuromusculaires, Hôpital Roger Salengro, Lille, France; 10 Service de Neuropédiatrie, Hôpital Roger Salengro, Lille, France; 11 Neuropédiatrie/INSERM CIC 1434, CHU Strasbourg Hautepierre, Strasbourg, France; 12 Paediatric neurology and neuromuscular center, University of Essen, Essen, Germany; 13 Consultants for Research in Imaging and Spectroscopy (CRIS), Tournai, Belgium; 14 Center for Gene Therapy, Nationwide Children's Hospital, Columbus, Ohio, USA; 15 Roche Pharmaceutical Research and Early Development, Roche Innovation Center, Basel, Switzerland; 16 Institut Roche, Paris, France; 17 Service de Pédiatrie, CHU de Liège, Liège, Belgium; UPMC, FRANCE

## Abstract

Spinal muscular atrophy (SMA) is a monogenic disorder caused by loss of function mutations in the *survival motor neuron 1* gene, which results in a broad range of disease severity, from neonatal to adult onset. There is currently a concerted effort to define the natural history of the disease and develop outcome measures that accurately capture its complexity. As several therapeutic strategies are currently under investigation and both the FDA and EMA have recently approved the first medical treatment for SMA, there is a critical need to identify the right association of responsive outcome measures and biomarkers for individual patient follow-up. As an approved treatment becomes available, untreated patients will soon become rare, further intensifying the need for a rapid, prospective and longitudinal study of the natural history of SMA Type 2 and 3. Here we present the baseline assessments of 81 patients aged 2 to 30 years of which 19 are non-sitter SMA Type 2, 34 are sitter SMA Type 2, 9 non-ambulant SMA Type 3 and 19 ambulant SMA Type 3. Collecting these data at nine sites in France, Germany and Belgium established the feasibility of gathering consistent data from numerous and demanding assessments in a multicenter SMA study. Most assessments discriminated between the four groups well. This included the Motor Function Measure (MFM), pulmonary function testing, strength, electroneuromyography, muscle imaging and workspace volume. Additionally, all of the assessments showed good correlation with the MFM score. As the untreated patient population decreases, having reliable and valid multi-site data will be imperative for recruitment in clinical trials. The pending two-year study results will evaluate the sensitivity of the studied outcomes and biomarkers to disease progression.

**Trial Registration**: ClinicalTrials.gov (NCT02391831).

## Introduction

Spinal muscular atrophy (SMA) is the second most frequent autosomal recessive disorder worldwide. It is caused by the functional loss of the *SMN1 (survival motor neuron 1)* gene. However, the nearly identical gene, *SMN2 (survival motor neuron 2)*, remains resulting in reduced production of full‐length SMN protein [1, 2]. Decreased SMN protein leads to spinal motor neuron degeneration and neuromuscular junction dysfunction, which predominately appears clinically as proximal muscle weakness, hypotonia and muscle atrophy [3].

While SMA is a monogenic disorder, it displays a broad range of severity, and affects infants through to adults. SMA is classified by the maximal achieved motor milestone [3]: SMA Type 1 (never sit independently and generally do not live past 2 years of age), SMA Type 2 (sit but never walk independently), SMA Type 3 (stand and walk independently). The phenotype varies between and within each SMA Type, covering a wide range of functional abilities, but also progresses over time with motor function loss [4–6]. The main determining factor for clinical severity is the number of *SMN2* copies [7]. Yet, additional gene modifiers are under investigation [6].

Currently, there is a concerted effort to define the natural history of the disease and develop outcome measures [8–10]. Primary outcome measures of choice in current clinical trials in SMA are motor function scales (NCT02292537/Hammersmith Functional Motor Scale-Expanded, NCT02908685/MFM). To measure the complexity of the phenotype range, other outcome measures relevant to SMA such as muscle strength, electrophysiological assessments, quality of life as well as genetic biomarkers are integrated into clinical trial methods [11]. Further potential but much less studied target outcome measures include muscle imaging, workspace volume and daily activity reporting [12–14].

The FDA and EMA recently approved Nusinersen, the first medical therapy for all types of SMA. This major step forward provides the first approved pharmaceutical treatment option for individuals with SMA. Nusinersen has shown documented efficacy [15–17]. Nevertheless, the treatment administration is demanding and its long term efficacy has not yet been evaluated. Also, several therapeutic trials designed to increase SMN protein expression are currently underway (NCT02122952, NCT02908685, NCT02628743, NCT02913482). Therefore, clinical trials in SMA are still necessary to evaluate other potential treatments. This has intensified the need to identify reliable outcome measures for clinical trials and most particularly to stratify patient populations and define the specific target windows for treatments. Indeed, clinically meaningful, statistically robust and complementary outcome measures should decrease sample sizes and study length. Individual patients would also benefit as appropriate outcome measures could identify early good responders to new medications as well as avoid exposing non-responders to long-term expensive treatments.

As the number of untreated patients will likely decrease rapidly, we took this window of opportunity to gather prospective, longitudinal natural history data from a large Type 2 and 3 SMA cohort over 2 years. The objective of the study is to identify prognostic variables and biomarkers of SMA progression by using a broad range of carefully chosen, standardized evaluations and assessments. We present here the baseline data. The objective of this manuscript is to evaluate if these outcome measures and biomarkers define the different levels of the disease phenotype (muscle atrophy and its subsequent muscle weakness) and whether they can differentiate patients with various levels of disease severity and correlate with the motor function.

## Patients and methods

The protocol for this trial and supporting TREND checklist are available as supplementary information (S1 Protocol and S1 Checklist).

### Study design and patients

NatHis-SMA is a European, prospective, multicenter, longitudinal natural history study of Type 2 and 3 SMA conducted in nine reference centers for neuromuscular diseases in France, Belgium and Germany (S1 Protocol) between May 2015 and May 2018.

The protocol was approved in France by the regulatory authority and the central Ethics Committee and by the local Ethics Committees in Liège, Leuven and Essen. Before inclusion, all patients or their parent(s)/legal guardian(s) provided written informed consent. The study is registered on ClinicalTrials.gov (NCT02391831).

Enrollment was restricted to children and adults between 2 and 30 years old. Non-ambulant patients needed to tolerate sitting in a wheelchair for a minimum of three hours. Patients were excluded from the study for recent or current exposure to an investigational SMA treatment or if they had a comorbid condition that could significantly interfere with disease assessment. Pregnant or breastfeeding women were excluded. Patients with specific contraindication to Magnetic Resonance Imaging (MRI) were able to participate, but did not receive an MRI.

**Fig 1:**
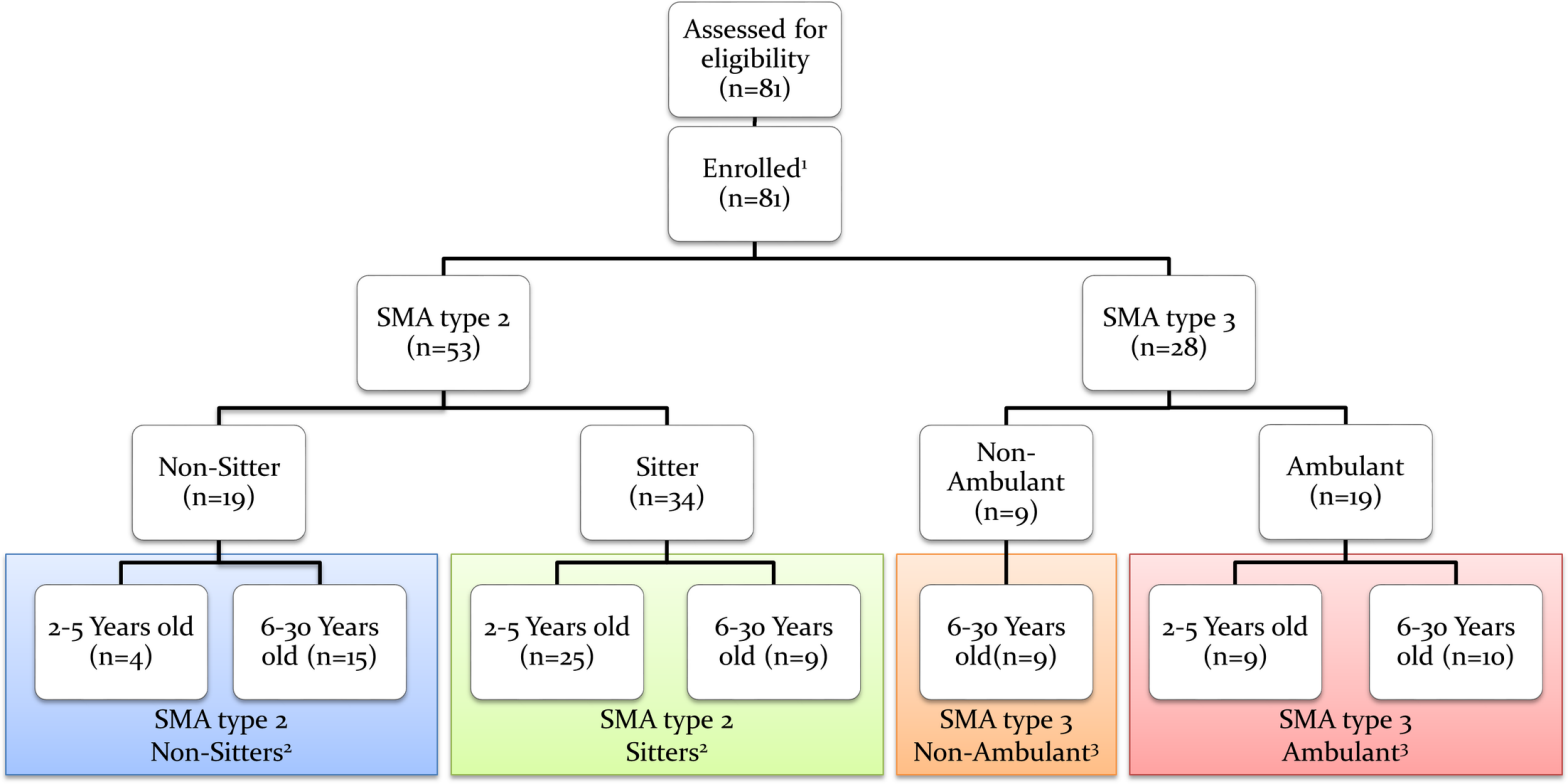
CONSORT diagram. ^1^ As this manuscript was limited to baseline data, there were no withdrawal of participants. ^2^ To be defined as “sitter” the patient must have a score ≥ 1 on item 9 of the MFM (“with support of one or both upper limbs maintain the seated position for 5 seconds”). For 3 patients the MFM could not be performed at baseline. 2 were classified as sitters since they had a score of 3 at the 6-month follow-up visit and 1 was retrospectively classified as a non-sitter according to patient files. ^3^ To be defined as “Ambulant” the patient must be able to walk 10 meters without human assistance or use of an ambulation device such as a cane or a walker.

The study required administrating the full battery of outcome measures at baseline, and then every 6 months (± 28 days) for the 24 months of the study, with the exception of the MRI, which is only performed every 12 months. Assessments were adjusted for patient age (2 to 5 years old and 6 to 30 years old) and ambulant status (ambulant or non-ambulant) to account for variations in phenotype and age (Fig 1). To be defined as “ambulant” the patient must be able to walk 10 meters without human assistance or use of an ambulation device such as a cane or a walker.

Inter or intra rater reliability has been well described for most of the assessments [13, 18–22], thus they were performed once at scheduled time point. This avoided unnecessary fatigue and burden to patients.

To minimize inter and intra site variability, the order in which each evaluation was to be performed was clearly stipulated in the protocol (S1 Protocol). Study sites were reminded to adhere to this schedule. Visits lasted 1 to 2 consecutive days depending on patient’s age and fatigability.

### Study procedures and outcomes

#### Clinical evaluation

At the baseline visit, a complete medical history was obtained including data related to perinatal period, SMA diagnosis and genetic confirmation, family history and hospitalizations in relation with SMA as well as previous and concomitant diseases.

At every visit, a full physical examination was performed including weight and height, upper and lower limb contractures, ambulant status and Brooke score (upper extremity function). Predicted height and weight have been calculated according to Flanders, Belgium normal reference ranges [23]. Adverse Events (AE), events notable for disease progression but not constituting an AE, concomitant treatments and the number of physiotherapy sessions per week were also reported. AE and Serious Adverse Events (SAE) were defined in the section 10 of the protocol (S1 Protocol).

**Disease progression**

In order to collect data from the usual SMA clinical follow-up, the investigators completed a survey at each visit from patient files and parent/patient interviews (S1 Protocol). The aim was to quantify motor development, cognition, respiratory function, feeding and orthopedic status, level of care required and disease burden. For patients without corrective surgery, scoliosis was evaluated in the sitting position. The Cobb angle was collected when available in medical records (X-ray measures).

#### Motor function assessments

**Motor Function Measure (MFM)**

The MFM is a linear scale with a 0 to 100% score, which assesses standing position, ambulation and transfers (D1 sub-score), axial and proximal motor function (D2 sub-score), and distal motor function (D3 sub-score) [18]. Higher scores reflect a higher level of function. The MFM-32 is used for patients older than 6 years old, and the MFM-20 for children between 2 and 5 years old [18, 24–26]. For patients between 4 and 6 years old, the physiotherapist selected either the MFM-20 or MFM-32 based on the child’s abilities, and was instructed to administer the same scale throughout the study.

**Moviplate**

Dominant and non-dominant upper arm function was assessed with the Moviplate device on patients older than 6 years old as previously described [19, 27]. Patients were given at least two attempts on each side. If they improved between attempts, they could perform one additional one and the maximal value was recorded. A higher number of taps indicates a higher level of upper limb function.

**Active-Seated**

Upper extremity Functional Reaching Volume (FRV) was measured in patients older than 6 years with Active-Seated as previously described [13]. Compensatory movements were permitted. The raw FRV was converted into percent predicted FRV (ppFRV) based on estimated height calculated using the ulnar length. The best ppFRV of three testing sessions was retained. A higher workspace volume reveals a higher level of upper limb function.

**Activity monitoring**

The ActiMyo^®^ device (Sysnav, Vernon, France) measures patient physical activity using sensors in three dimensions [14] (S1 Protocol). The daily use of the device was proposed to a subset of 30 non-ambulant patients older than 6 years. One device is worn as a wristwatch and the other is worn on the wheelchair. Five variables representing upper limb activity were generated: the wrist angular velocity (||Ω||), the wrist acceleration (||A||), the wrist vertical acceleration against gravity (vA), the power (P) and the percentage of activity time. Data presented here is recorded over the first 180 hours, which represents the minimal period of time with the lowest variability of ActiMyo® variables.

#### Muscle strength assessments

**Pulmonary function tests (PFT)**

Patients older than 6 years performed forced vital capacity (FVC), peak cough flow (PCF), maximum expiratory and inspiratory pressures (MEP and MIP) and sniff nasal inspiratory pressure (SNIP) assessments in the sitting position. FVC and PCF were captured with the Vitalograph spirometer and software, while MEP, MIP and SNIP were evaluated using the MicroRPM device. Patients weight and height measured by investigator or study nurse were transmitted to physiotherapists for entry into the Vitalograph software. Results are expressed in percent of predicted values based on age and height [28–32]. The best result of three measurements was retained.

**Upper arm strength and function evaluation**

Dominant and non-dominant upper arm strength was assessed with the MyoGrip and MyoPinch devices in patients older than 6 years as previously described [19, 27]. Patients were given at least three attempts on each side. If they improved between attempts, they could perform up to two additional ones. Results are expressed as a percentage of predicted values based on hand circumference [19, 33]. The maximal value was used for analysis. Higher values reflect higher level of upper arm strength and function.

**Timed tests**

Ambulant patients older than 6 years performed the Six-Minute Walk Test (6MWT) [34], 10 Meter timed Walk/run Test (10MWT), Time to Rise from Floor test (TRF) [35], time to climb and time to descend 4 standardized stairs.

**Electrophysiology measurement**

Neuromuscular transmission was tested by 3 Hz repetitive nerve stimulation performed on 4 nerves of the dominant side: accessory nerve, recording *trapezius* muscle; radial nerve, recording *anconeus* muscle; ulnar nerve, recording *adductor digiti minimi* (ADM) muscle; fibular nerve, recording *tibialis anterior* muscle. Compound Motor Action Potentials (CMAP) were recorded through surface electrodes, using standard placement. The amplitude and area decrements was defined as the ratio between the first and fifth CMAP in response to repetitive nerve stimulation.

#### MRI imaging

**Data acquisition**

Muscle MRI was performed at 2 study sites, Paris and Strasbourg, in a subset of 20 patients. The examination was performed without sedation at baseline and at one-year intervals on the dominant upper arm for all patients, and on the lower limbs for ambulant patients.

Quantitative water-fat imaging was obtained using a 3-point Dixon 3D gradient echo sequence. The images were obtained from the mid-humerus and proximal third of radius-cubitus for the upper limbs. In ambulant patients, the lower-limbs image acquisition was centered at mid-thigh and proximal third of the leg. Transverse relaxometry is achieved using a 2D multi-slice multi-echo (MSME) sequence.

**Data processing**

All MRI data were centralized and processed in a consistent manner. The in- and out-phase Dixon images were used to compute fat and water signal maps with standard 3-point reconstruction technique [36], from which fat-fraction maps *FF*_*dix*_ were derived. A multi-exponential signal model was fit onto the Multi Slice Multi Echo (MSME) data to generate water transverse relaxation (T2) maps as well as fat-fraction maps *FF*_*T2*_ [37, 38].

The MRI biomarkers were measured for several muscles or muscle groups in each segment. On the dominant upper limb, the *Triceps Brachii*, *Biceps Brachii*, Forearm *Flexors* and *Extensors* and on the lower legs, the *Quadriceps*, *Biceps Femoris*, *Semi Tendinosus*, *Semi Membranosus*, *Tibialis Anterior*, *Peroneus Lateralis* and *Triceps Suralis* were imaged. The Regions Of Interest (ROI) drawn on the MSME images were used in combination with the T2 and *FF*_*T2*_ maps to provide the mean T2 and fat-fraction values in all muscle groups.

ROI were manually drawn using an in-house interactive segmentation [39] software on the MRI images for all aforementioned regions on up to 5 slices of the Dixon and MSME images. The Dixon-based ROI were used to measure the cross-sectional area (CSA) of the muscle groups, that is, the mean area of the muscle axial section. Secondly, the same ROIs were used in combination with the fat-water mapping to provide the contractile CSA (C-CSA), which is computed as: *C-CSA* = *CSA*×(1−*FFdix*).

#### Molecular biomarkers

Venous blood was collected from patients using standard procedures in PAXgene (PreAnalytiX), p700, p800 and K_3_EDTA tubes (BD Bioscience), and processed and frozen according to manufacturer’s instructions, except for PAXgene tubes that were frozen immediately after blood collection and tube inversions. Samples were analyzed by Roche Laboratories using recently published assays [7, 12]. Briefly, DNA extraction from K_3_EDTA samples was performed using the MagNa Pure 96 instrument according to manufacturer’s instructions. *SMN1* and *SMN2* copy numbers were determined from 80 ng DNA input using a digital droplet PCR (ddPCR) approach. In parallel, RNA was isolated from the PAXgene samples and stored at—80°C before analysis. mRNA expression levels for the different *SMN* isoforms (*SMN1*, *SMN2* and *SMND7*) and the housekeeping gene were assessed by a multiplex qRT-PCR assay as described previously [7]. For SMN protein analysis, blood was collected in p700 tubes to stabilize the protein and after inverting immediately frozen. SMN protein levels were measured by the SMN research assay developed by Roche Diagnostics on the Elecsys platform in 1:2 dilutes samples as previously described [7, 12]. A correction factor of 0.86 was applied to the measured SMN protein values to correct for the switch to p800 tubes in March 2017 due to discontinuation of p700 tube supply. This correction factor has been determined by Roche Laboratories following experiments comparing SMN protein concentrations measured in blood from the sample donors collected in both tube types.

#### Training and data quality control

Prior to collecting data, the certified physiotherapists attended a specific protocol training. Assessments were administered according to a standardized procedure manual. Additional on-site refresh training was provided after the first patient evaluation by Institute of Myology certified staff.

Study monitors regularly evaluated protocol and GCP compliance as well as quality, completeness and consistency of the data collected in the case report forms at all sites. Additionally, data quality control was performed centrally on MyoSet (MyoGrip, MyoPinch, Moviplate) and Active-Seated data was collected at each site. CMAP curves were sent to the coordinating site for central review.

#### Statistical analysis

Statistical analyses were performed following pre-specified statistical analysis plan after database lock for baseline data (S1 Statistical Analysis Plan). Four functional groups were defined for comparison analysis: non-sitter patients with SMA Type 2, sitter patients with SMA Type 2, non-ambulant patients with SMA Type 3 and ambulant patients with SMA Type 3. The ambulant status is defined in the Methods section. To be defined as “sitter” the patient must have a score ≥1 on item 9 of the MFM (“with support of one or both upper limbs maintain the seated position for 5 seconds”). Characteristics of the four groups were computed by descriptive statistical analysis (median, Interquartile range (IQR), number). To determine outcome measures able to differentiate patients with different levels of the disease phenotype, proportions were compared between groups using Chi-square test and quantitative variables were compared using the Kruskal-Wallis one-way analysis of variance. Post hoc analyses based on Dunn-Bonferroni method were added where the significance was found. The side effect on upper limb strength assessment was assessed with a Wilcoxon signed-rank test. The relationships between the MFM and outcomes measures were examined using Spearman’s correlation coefficients. All analyses were performed using the IBM SPSS Statistics 22 statistical software (SPSS Inc., Chicago, IL). The limit of statistical significance was set to 0.05.

## Results

### Baseline clinical and physical characteristics and psychomotor development

#### Clinical condition

81 patients with SMA Type 2 (n = 53) and Type 3 (n = 28) were enrolled (Fig 1). Only three patients from one site previously participated in a therapeutic study (2 patients in trial NCT01302600/olesoxime and 1 patient in trial NCT00774423/riluzole/placebo).

Baseline clinical and physical characteristics are summarized in Table 1 and S1 Table.

**Table 1:** Baseline clinical and physical characteristics and of enrolled patients.

				SMA type 2		SMA type 3		Overall (n = 81)
				Non-Sitter (n = 19)	Sitter (n = 34)	Non-Ambulant (n = 9)	Ambulant (n = 19)	
**Age at enrollment (y)** **				14.9 _a_(6.3–17.2)	4.6 _b_ (2.5–7.1)	19.6 _a_ (15.3–25.6)	10.4 _a, b_ (4.5–19.2)	7.1 (3.8–16.5)
**Age at first symptoms (months; n = 80)** **				9.0 _a_ (6.0–15.0)	9.0 _a_ (7.0–12.0)	21.0 _b_ (13.0–33.0)	22.0 _b_ (17.0–32.0)	12.0 (8.0–18.0)
**Age at diagnosis (months)** **				16.0 _a_ (12.7–20.0)	17.6 _a_ (13.0–19.7)	30.3_b_ (25.9–52.7)	35.8 _b_ (25.4–56.4)	19.7 (14.6–28.7)
***SMN1* copy number** ^**1**^^**-**^^**3**^				*n = 17*	*n = 34*	*n = 9*	*n = 18*	*n = 78*
*0 (n)*				*17*	*33*	*9*	*17*	*76*
*1 (n)*				*0*	*1*	*0*	*1*	*2*
***SMN2* copy number** ^**1**^^,^ ^**2**^ * ^□^				*n = 17*	*n = 34*	*n = 9*	*n = 18*	*n = 78*
*2 (n)*				*1*	*1*	*1*	*0*	*3*
*3 (n)*				*16* _a_	*31* _a_	*5* _b_	*13* _a, b_	*65*
*4 (n)*				*0* _a_	*2* _a_	*3* _b_	*5* _b_	*10*
**Hospitalization number** **				8 _a_ (4–10)	2 _c_ (0–4)	0 _c_ (0–3)	0 _c_ (0–1)	2 (0–5)
**Hospitalization duration (days)** **				46_a_ (23–66)	13 _b_ (3–27)	23 _a, b_ (18–32)	4_b_ (0–11)	20 (6–42)
**Weight (predicted for age, %)** *				0.02 _a_ (0.00–21.94)	14.47 _a, b_ (1.03–42.07)	2.13 _a, b_ (0.08–72.42)	24.28_b_ (14.04–46.70)	14.15 (0.57–44.10)
**Height (predicted for age, %)** **				2.07 _a_ (0.12–7.58)	10.72 _a, b_ (2.75–28.84)	5.68 _a, b_ (1.14–31.96)	32.77_b_ (8.95–53.77)	8.95 (2.01–32.70)
**Respiratory rate (breath/min; n = 71)** *				26 _a_ (18–32)	24 _a_ (22–28)	16_b_ (15–20)	24 _a, b_ (20–26)	24 (12–90)
**Upper limb contractures (%)**								
	Fingers (n)			*6*	*3*	*1*	*1*	*11*
			*Flexion*	*1*	*2*	*0*	*1*	*4*
			*Extension* *^□^	*6* _a_	*2* _a, b_	*1* _a, b_	*0* _b_	*9*
	Wrist (n) * ^□^			*9* _a_	*2*_b_	*1* _a, b_	*1*_b_	*13*
			*Flexion*	*1*	*0*	*0*	*1*	*2*
			*Extension* ** ^□^	*9* _a_	*2*_b_	*1* _a, b_	*0*_b_	*12*
	Elbow (n) ** ^□^			*12* _a_	*6*_b_	*0*_b_	*0*_b_	*18*
			*Flexion*	*2*	*1*	*0*	*0*	*3*
			*Extension* ** ^□^	*12* _a_	*6*_b_	*0*_b_	*0*_b_	*18*
**Concomitant treatments at enrolment** ^**4**^								
*Treated patients (n)* * ^□^				*15* _a_	*22* _a, b_	*4* _a, b_	*6*_b_	*47*
*Treatment(s) per patient* **				4 _a_ (1–8)	2 _a, b_ (1–4)	1_b__, c_ (0–1)	0 _c_ (0–1)	2 (0–4)
**Physiotherapy (n)** ** ^□^				*19* _a, b_	*34* _b_	*7* _a_	*19* _a, b_	*79*
*Session(s) / week per patient* *				3 _a_ (2–3)	3 _a_ (2–4)	3 _a, b_ (2–3)	2_b_ (1–2)	2.5 (2–3)
**Respiratory function (n)**								
		Respiratory lower track infection during the previous year ** ^□^		*13* _a_	*22* _a_	*0*_b_	*3*_b_	*38*
			*Infections number*	2 (1–3)	2 (1–3)	-	1 (1–2)	2 (1–3)
		Sleep apnoea		*3*	*2*	*0*	*0*	*5*
		Breathing support ** ^□^		*15* _a_	*18* _a, b_	*1* _b, c_	*1* _c_	*35*
			*Non-Invasive Ventilation* *^□^	*15* _a_	*14* _a_	*0*_b_	*0*_b_	*29*
			*Invasive Ventilation*	*0*	*1*	*0*	*0*	*1*
			*Cough assist*	*8*	*8*	*0*	*0*	*16*
			*Intermittent Positive Pressure Ventilation*	*8*	*11*	*1*	*1*	*21*
**Feeding difficulties (n)** ** ^□^				*12* _a_	*12* _a, b_	*1* _a, b_	*1*_b_	*26*
*Current feeding tube*				*3*	*2*	*0*	*0*	*5*
*Past feeding tube*				*6*	*4*	*0*	*1*	*11*
*Nasogastric tube*				*3*	*3*	*0*	*1*	*7*
*Gastrostomy*				*4*	*2*	*0*	*0*	*6*
**Orthopaedic status (n)**								
		***Scoliosis*** ** ^□^		*17* _a_	*18*_b_	*6* _a, b_	*4* _c_	*45*
		Angle		*n = 3*	*n = 10*	*n = 5*	*n = 4*	*n = 22*
		*< 30°*		*3*	*5*	*5*	*4*	*17*
		*30–45°*		*0*	*1*	*0*	*0*	*1*
		*> 45°*		*0*	*4*	*0*	*0*	*4*
		Arthrodesis ** ^□^		*13* _a_	*4*_b_	*1* _a, b_	*0*_b_	*18*
		*Age at surgery (year)*		10 (9–12)	13 (7–15)	10 (-)	-	11 (9–12)
		*Type of surgery*:		*n = 9*	*n = 4*	*n = 1*	*n = 0*	*n = 14*
		*Fusion*		*5*	*3*	*1*	*0*	*9*
		*Growing rods*		*4*	*1*	*0*	*0*	*5*
		***Fracture***		*7*	*7*	*0*	*3*	*17*
		***Assistive device*** ** ^□^		*19* _a_	*33* _a_	*9* _a, b_	*13*_b_	*74*
		*Therapies other than physiotherapy*		*6*	*14*	*5*	*7*	*32*
			*Swimming*, *balneotherapy*, *massage*	*4*	*7*	*5*	*7*	*23*
			*Osteopathy*, *acupuncture*	*1*	*6*	*2*	*0*	*9*
			*Occupational therapy*	*2*	*8*	*1*	*1*	*12*
			*Speech Therapy*	*0*	*2*	*0*	*0*	*2*
**Caregiver required (6–30 years old)**^**5**^ ** ^□^				*n = 14*	*n = 8*	*n = 8*	*n = 10*	*n = 40*
				*14* _a_	*8* _a, b_	*4* _b, c_	*3*_c_	*29*

Values are median (IQR) and overall population size is n = 81 unless otherwise indicated

^1^ Baseline samples could not be collected for one 2-year-old non-sitter SMA type 2 patient due to non-compliance during the procedure

^2^ Baseline DNA samples of two patients could not be analyzed due to scarce amount of material collected (one non-sitter patient with SMA type 2 and one ambulant patient with SMA type 3)

^3^ According to their genetic initial diagnosis, two patients had 1 SMN1 copy number due to loss of function point mutations: c.779T>C (p.Leu260Ser) for one sitter patient with SMA type 2, and c.815A>G (p.Tyr272Cys) for one ambulant patient with SMA type 3

^4^ Concomitant treatments at enrollment included treatments prescribed for SMA: oral salbutamol for 1 non-sitter type 2 patient and 1 ambulant patient, and levocarnitine for 3 type 2 patients (1 sitter and 2 non-sitter)

^5^ Data not available for 1 non-sitter patient with SMA type 2, 1 sitter patient with SMA type 2 and 1 non-ambulant patient with SMA type 3

* p ≤ 0.05

** p ≤ 0.001

^□^ Application conditions of the Chi-square test not fully verified (theoretical effectives ≤ 5, too small effectives)

_a, b, c_ Subscript letters represent Post-hoc tests results. In a row, a same subscript letter indicates a subset of categories (non-sitter SMA type 2, sitter SMA type 2, non-ambulant SMA type 3 and ambulant SMA type 3) which do not differ significantly from each other at level 0.05.

All patients in this study had a genetically confirmed homozygous loss of function of *SMN1* on exon 7 and showed symptoms consistent with classical Type 2 or 3 phenotypes. As expected, medical history was stratified between groups based on age at first symptoms, number of *SMN2* copies, pulmonary events and hospitalizations. Birth weight, height, length and head circumference were within normal ranges. Non-ambulant patients with SMA Type 3 were the oldest, while sitter patients with SMA Type 2 were the youngest. Weight, height, respiratory rate, heart rate and diastolic blood pressure were significantly different between groups as expected for each age group (younger infants have lower blood pressure and higher heart rate). Heart rate and blood pressures were within normal ranges, consistent with the absence of cardiac impairment in this disease. Weight and height were lower compared to normal values for age, especially in the older groups.

Individuals with SMA Type 2 showed more severe musculoskeletal abnormalities (including contractures of the distal joints), respiratory difficulties (lower respiratory tract infections, sleep apnea, use of non-invasive ventilation and respiratory supports), feeding problems (difficulties for sucking, swallowing or chewing, reflux/vomiting and need for gastrostomy) and orthopedic problems (scoliosis, arthrodesis, fractures). Almost all patients received physiotherapy (n = 79), with ambulant patients receiving the lowest frequency of sessions. The majority of patients reported using at least one assistive device. This included the vast majority of non-ambulant individuals and about two-thirds of ambulant individuals. Patients with SMA also turned towards non-standard therapies like osteopathy, balneotherapy, swimming, massage, acupuncture, occupational therapy. No cognitive impairment was reported. All patients aged between 6 and 18 years were attending school, and 65% of patients older than 18 years had some level of higher education. Among patients over 6 years old, those with SMA Type 2 needed a caregiver for daily activities. Among patients with SMA Type 3, 50% of non-ambulant and 30% of ambulant patients were self-caring in connection with the lower disease burden in those patients.

#### Psychomotor development

**Fig 2:**
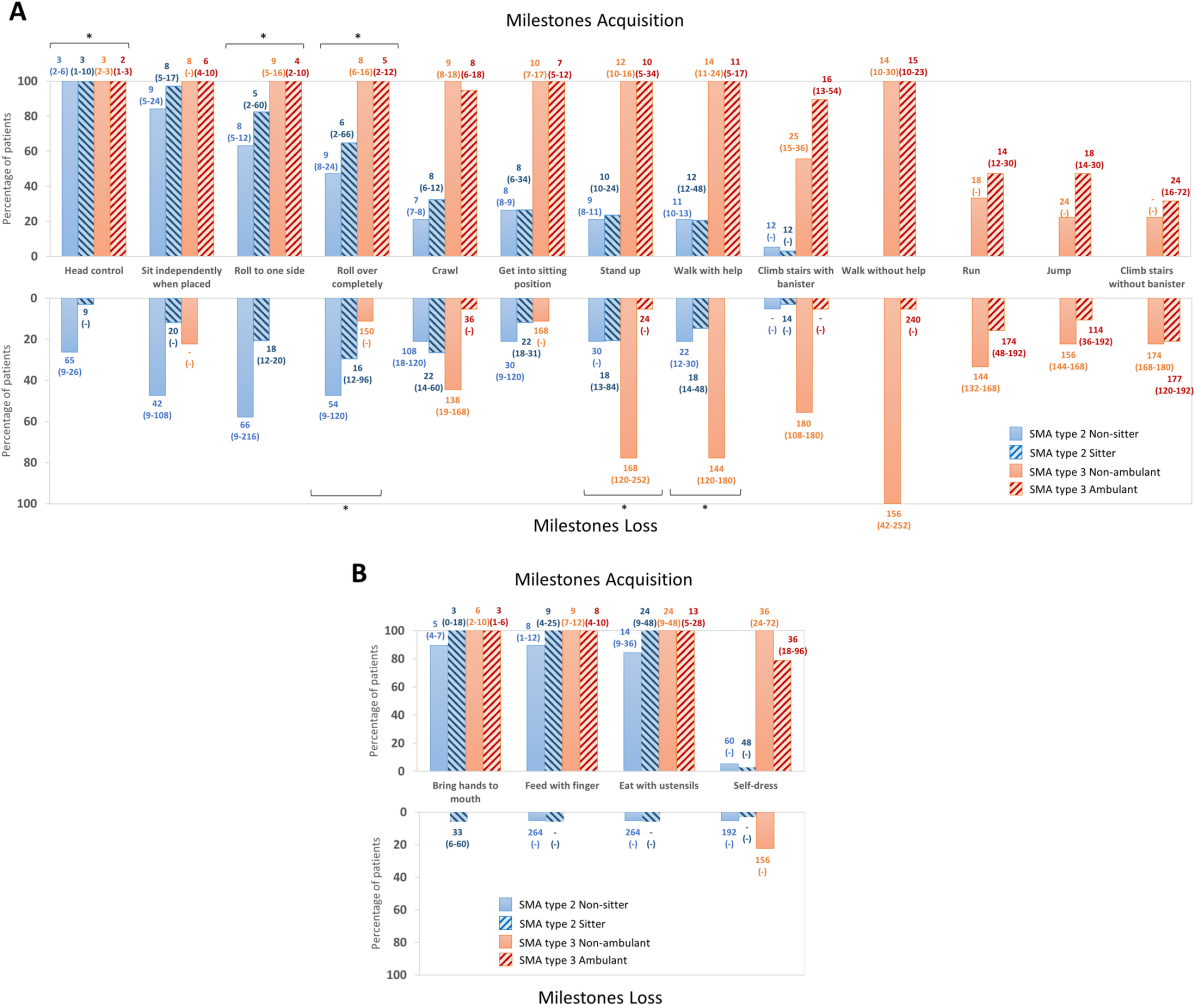
Psychomotor development of patients with SMA type 2 and 3. A: General motor development; B: Fine motor development; Values for ages displayed on histograms are median (min-max); * p ≤ 0.05.

All patients with SMA Type 3 had acquired the ability to sit and walk without help but fewer than 50% of patients with SMA Type 3 in this study were able to run, jump or climb stairs unassisted (Fig 2 and S2 Table). Fewer patients with SMA Type 2 acquired rolling, crawling, sitting, standing and self-dressing abilities when compared to patients with SMA Type 3. We also collected the age that individuals obtained motor milestones such as developing head control and rolling to one side. The age was significantly different between the groups: the ambulant patients achieved these skills at the youngest age followed by non-ambulant patients with SMA Type 3, then the sitter patients with SMA Type 2 and finally the non-sitter individuals with SMA Type 2.

The first loss of abilities by patients with SMA Type 3 occurred on average after the age of 9 years, whereas patients with SMA Type 2 first lost abilities before the age of 2 years (Fig 2). Indeed, patients with SMA Type 2 lost the ability to stand up and walk with help, if acquired, significantly earlier than non-ambulant SMA Type 3 patients. Most patients with SMA Type 2 lost rolling, crawling and self-dressing abilities, while fewer than a half of patients with SMA Type 3 lost these skills. Finally, patients with SMA Type 3 lost the ability to walk independently around the age of 12 years which is on average 10 years after the first disease symptoms (Table 1).

#### Baseline muscle weakness

**Motor function**

All MFM, Moviplate and Active-Seated scores increased from the most severely affected non-sitter patients with SMA Type 2 to the less severely affected ambulant patients with SMA Type 3 (Table 2). MFM total scores were variable in the youngest patients but then slowly decreased with age (S1 Fig). MFM D2 sub-scores were particularly correlated with age in patients with SMA Type 2 (S1 Fig; ρ = 0.60, p≤0.001). As expected, the Brooke upper limb scores were inversely proportional to disease severity. Eight non-sitter and five sitter patients with SMA Type 2 had their daily activity recorded with the ActiMyo^®^ device (Table 2). The median wrist angular velocity (||Ω||) was significantly higher in the sitter group when compared to the non-sitter group. The median of the wrist acceleration (||A||) was significantly correlated with the Moviplate score on the dominant side (ρ = 0.59; p = 0.04; n = 12). In addition, the 95^th^ percentile of the wrist vertical acceleration (vA) was significantly correlated with the upper limb reaching volume measured by Active-Seated (ρ = 0.75; p = 0.05; n = 8). The complete data for the ActiMyo recording will be presented elsewhere.

**Table 2:** Motor function in SMA type 2 and 3 patients.

	SMA type 2		SMA type 3		Overall
	Non-sitter	Sitter	Non-Ambulant	Ambulant	
**MFM-20 score** ^**1**^	*n = 3*	*n = 21*	*n = 0*	*n = 8*	*n = 32*
D1 (standing position and transfers)**	*4*.*2* _a_ *(0-*.*)*	*4*.*2*_a_ *(2*.*1–8*.*3)*	*-*	*56*.*2* _b_ *(43*.*7–68*.*7)*	*4*.*2 (4*.*2–36*.*4)*
D2 (axial and proximal motor function)**	*29*.*2* _a_ *(25*.*0-*.*)*	*75*.*0* _a_ *(64*.*6–83*.*3)*	*-*	*95*.*8* _b_ *(89*.*6–99*.*0)*	*83*.*3 (63*.*5–91*.*7)*
D3 (distal motor function)*	*66*.*7*_a_ *(50*.*0-*.*)*	*83*.*3* _a, b_ *(70*.*8–95*.*8)*	*-*	*100* _b_ *(93*.*7–100)*	*83*.*3 (66*.*7–100)*
Total**	*26*.*7* _a_ *(20*.*0-*.*)*	*48*.*3* _a_ *(40*.*8–54*.*2)*	*-*	*80*.*8*_b_ *(73*.*3–85*.*8)*	*51*.*7 (40*.*4–63*.*7)*
**MFM-32 score** ^**2**^	*n = 15*	*n = 11*	*n = 9*	*n = 11*	*n = 46*
D1 (standing position and transfers)**	*2*.*6* _a_ *(0–2*.*6)*	*2*.*6* _a, b_ *(2*.*6–2*.*6)*	*10*.*2* _b, c_ *(6*.*4–11*.*5)*	*66*.*7* _c_ *(51*.*3–79*.*5)*	*3*.*8 (2*.*6–31*.*4)*
D2 (axial and proximal motor function)**	*38*.*9*_a_ *(27*.*8–44*.*4)*	*63*.*9*_a, b_ *(52*.*8–80*.*6)*	*91*.*7* _b, c_ *(88*.*9–97*.*2)*	*97*.*2* _c_ *(91*.*7–100)*	*76*.*4 (41*.*0–94*.*4)*
D3 (distal motor function)**	*71*.*4* _a_ *(66*.*7–90*.*5)*	*80*.*9* _a, b_ *(66*.*7–90*.*5)*	*95*.*2* _b, c_ *(90*.*5–95*.*2)*	*100* _c_ *(95*.*2–100)*	*90*.*5 (70*.*2–95*.*2)*
Total**	*28*.*1*_a_ *(23*.*9–35*.*4)*	*42*.*7* _a, b_ *(35*.*4–48*.*9)*	*60*.*4* _b, c_ *(57*.*8–60*.*9)*	*84*.*4* _c_ *(70*.*1–90*.*6)*	*48*.*4 (35*.*1–67*.*4)*
**Brooke** ^**3**^ ** ^**□**^	*n = 16*	*n = 26*	*n = 9*	*n = 15*	*n = 66*
*1 (n)*	*0* _a_	*3* _a_	*6* _b_	*13* _b_	*22*
*2 (n)*	*2*	*9*	*2*	*2*	*15*
*3 (n)*	*8* _a_	*9* _a, b_	*1* _a, b_	*0* _b_	*18*
*4 (n)*	*5*	*4*	*0*	*0*	*9*
*5 (n)*	*1*	*1*	*0*	*0*	*2*
**Moviplate** ^**4**^	*n = 13*	*n = 9*	*n = 9*	*n = 10*	*n = 41*
Dominant side**	*47* _a_ *(38–58)*	*42* _a_ *(27–46)*	*71*_b_ *(62–78)*	*69* _b_ *(62–79)*	*54 (41–71)*
Non-dominant side**	*46* _a_ *(24–50)*	*40* _a_ *(32–50)*	*60* _b_ *(55–65)*	*65* _b_ *(51–70)*	*50 (40–64)*
**Active-Seated** ^**5**^	*n = 9*	*n = 5*	*n = 8*	*n = 6*	*n = 28*
Predicted FRV (%)**	*7*.*1* _a_ *(3*.*8–16*.*0)*	*9*.*5* _a, b_ *(7*.*1–18*.*4)*	*65*.*1* _b, c_ *(42*.*4–82*.*9)*	*76*.*0* _c_ *(57*.*2–108*.*0)*	*32*.*5 (7*.*4–66*.*1)*
**Home-monitoring by ActiMyo**^**® 6**^	*n = 8*	*n = 5*			*n = 13*
*Activity time (%)*	*46*.*9 (40*.*3–50*.*9)*	*59*.*4 (48*.*4–75*.*2)*			*48*.*1 (43*.*9–57*.*2)*
||Ω||*–median (°/s)* *	*14*.*7 (13*.*7–17*.*1)*	*18*.*1 (16*.*1–23*.*8)*			*15*.*3 (14*.*4–19*.*1)*
||Ω|| *- 95*^*th*^ *percentile (°/s)*	*111*.*2 (101*.*6–133*.*5)*	*136*.*9 (125*.*5–159*.*2)*			*115*.*4 (109*.*4–143*.*1)*
||A||*–median (m/s*^*2*^*)*	*0*.*040 (0*.*032–0*.*050)*	*0*.*048 (0*.*040–0*.*057)*			*0*.*040 (0*.*037–0*.*052)*
||A|| *- 95*^*th*^ *percentile (m/s*^*2*^*)*	*0*.*244 (0*.*194–0*.*285)*	*0*.*286 (0*.*220–0*.*333)*			*0*.*245 (0*.*205–0*.*304)*
*vA–median (m/s*^*2*^*)*	*0*.*015 (0*.*013–0*.*022)*	*0*.*019 (0*.*017–0*.*023)*			*0*.*018 (0*.*014–0*.*022)*
*vA - 95*^*th*^ *percentile (m/s*^*2*^*)*	*0*.*134 (0*.*111–0*.*177)*	*0*.*156 (0*.*131–0*.*197)*			*0*.*145 (0*.*122–0*.*181)*
*P–median (W/kg)*	*0*.*053 (0*.*049–0*.*061)*	*0*.*075 (0*.*055–0*.*095)*			*0*.*059 (0*.*050–0*.*074)*
*P - 95*^*th*^ *percentile (W/kg)*	*0*.*680 (0*.*551–0*.*777)*	*0*.*877 (0*.*623–1*.*051)*			*0*.*722 (0*.*579–0*.*906)*

Abbreviations: MFM: Motor Function Measure; FRV: Functional reaching volume; ||Ω||: norm of the angular velocity of the wrist; ||A||: norm of the acceleration of the wrist; vA: vertical acceleration of the wrist; P: power.

Values are median (IQR) and overall population size is n = 78 unless otherwise indicated

^1^ Thirty-two patients performed the MFM-20 scale; 1 sitter and 2 non-sitter patients with SMA type 2 aged 2 to 3 years old could not perform the MFM-20 due to lack of cooperation

^2^ Forty-six patients performed the MFM-32 scale

^3^ The Brooke level could not be evaluated in thirteen patients (3 non-sitter and 8 sitter patients with SMA type 2 and 4 ambulant patients)

^4^ Two non-sitter patients with SMA type 2 could not perform the Moviplate test due to a technical issue

^5^ Nine patients could not perform the Active-Seated test due to technical issues (n = 8) and patient compliance issue (n = 1 refused to perform the test because of fatigue). Six results were excluded from the analysis since these tests were performed under different conditions (with a second version of the software, including a calibration and performance of the test without any table)

^6^ In the initial protocol, the ActiMyo^®^ device was only proposed to non-ambulant patients with SMA type 2 aged over 6 years old. During the study this eligible population was broadened to patients with SMA type 3, including ambulant patients. Results presented here correspond to data recorded in thirteen patients with SMA type 2 who have been home using every day as part the trial. Measured parameters were averaged on a 180-hour period spread over two weeks.

* p ≤ 0.05

** p ≤ 0.001

^□^ Application conditions of the Chi-square test not fully verified (theoretical effectives ≤ 5, too small effectives)

_a, b, c_ Subscript letters represent Post-hoc tests results. In a row, a same subscript letter indicates subset of categories (non-sitter SMA type 2, sitter SMA type 2, non-ambulant SMA type 3 and ambulant SMA type 3) which do not differ significantly from each other at level 0.05.

**Pulmonary function**

Analysis of pulmonary function test results revealed that predicted FVC and MEP improved across the continuum of patient phenotype with the poorest values in the lowest functional group (non-sitter SMA Type 2) and the best values in the highest functional group (ambulant SMA Type 3) (Table 3). Predicted MIP and PCF values were significantly different between patients with SMA Type 2 and patients with SMA Type 3 with the lowest value in the former group.

**Table 3:** Pulmonary function in SMA type 2 and 3 patients.

	SMA type 2		SMA type 3		Overall (n = 43)
	Non-Sitter (n = 15)	Sitter (n = 9)	Non-Ambulant (n = 9)	Ambulant (n = 10)	
**Pulmonary function tests *(% of predicted values)***					
FVC (n = 41)**	*44* _*a*_ *(23–81)*	*62* _*a*, *b*_ *(37–83)*	*90* _*b*_ *(77–105)*	*96* _*b*_ *(82–107)*	*81 (43–92)*
MEP (n = 43)**	*25* _*a*_ *(21–33)*	*43* _*a*, *b*_ *(24–47)*	*72* _*b*, *c*_ *(51–97)*	*75* _*c*_ *(59–98)*	*44 (25–72)*
*MIP (n = 42)*	*63 (43–100)*	*61 (56–81)*	*110 (64–129)*	*99 (80–124)*	*78 (55–111)*
PCF (n = 40)*	*43* _*a*_ *(34–62)*	*68* _*a*, *b*_ *(51–79)*	*88* _*b*_ *(67–112)*	*79* _*b*_ *(70–88)*	*69 (45–84)*
*SNIP (n = 43)*	*35 (22–64)*	*33 (26–56)*	*56 (27–99)*	*45 (35–76)*	*39 (28–60)*

Abbreviations: FVC: Forced Vital Capacity; MEP: Maximum Expiratory Pressure; MIP: Maximum Inspiratory Pressure; PCF: Peak Cough Flow; SNIP: Sniff Nasal Inspiratory Pressure

Values are median (IQR) and overall population size is n = 81 unless otherwise indicated

* p ≤ 0.05

** p ≤ 0.001

_a, b, c_ Subscript letters represent Post-hoc tests results. In a row, a same subscript letter indicates subset of categories (non-sitter SMA type 2, sitter SMA type 2, non-ambulant SMA type 3 and ambulant SMA type 3) which do not differ significantly from each other at level 0.05.

**Upper limb muscle strength**

Differences were also seen in skeletal muscle strength. Remarkably, strength, expressed in percent of predicted values for hand circumference, showed a five to seven-fold increase in both grip and pinch strength between the ambulant SMA Type 3 group and the sitter SMA Type 2 group (Table 4).

**Table 4:** Muscle strength in SMA type 2 and 3 patients.

	SMA type 2		SMA type 3		Overall (n = 42)
	Non-Sitter (n = 15)	Sitter (n = 8)	Non-Ambulant (n = 9)	Ambulant (n = 10)	
**Myotools** ^**a**^					
Hand circumference (cm)					
Dominant side**	*15*.*2* _*a*_ *(14*.*8–17*.*0)*	*15*.*0* _*a*_ *(14*.*4–16*.*7)*	*20*.*0* _*b*_ *(19*.*1–20*.*7)*	*19*.*7* _*b*_ *(18*.*2–21*.*3)*	*17*.*0 (15*.*0–20*.*0)* ^*ΔΔ*^
Non-dominant side**	*15*.*1* _*a*_ *(14*.*7–16*.*5)*	*14*.*9* _*a*_ *(14*.*3–17*.*0)*	*19*.*5* _*b*_ *(15*.*3–21*.*0)*	*19*.*0* _*b*_ *(17*.*0–22*.*0)*	*16*.*8 (16*.*8–19*.*4)*
Myogrip *(% of predicted values for hand circumference)*					
Dominant side**	*8*.*7* _*a*_ *(5*.*5–12*.*2)*	*9*.*5* _*a*_ *(5*.*8–12*.*1)*	*24*.*9* _*a*, *b*_ *(18*.*8–27*.*1)*	*46*.*6* _*b*_ *(29*.*1–71*.*4)*	*15*.*0 (15*.*0–27*.*8)*
Non-dominant side**	*7*.*8* _*a*_ *(4*.*9–11*.*5)*	*10*.*3* _*a*, *b*_ *(9*.*2–12*.*9)*	*19*.*7* _*b*, *c*_ *(15*.*3–34*.*3)*	*48*.*5* _*c*_ *(27*.*2–70*.*4)*	*13*.*7 (13*.*7–29*.*3)*
Myopinch *(% of predicted values for hand circumference)*					
Dominant side**	*8*.*8* _*a*_ *(6*.*1–9*.*8)*	*13*.*9* _*a*, *b*_ *(8*.*4–16*.*6)*	*47*.*8* _*b*, *c*_ *(29*.*6–62*.*1)*	*68*.*0* _*c*_ *(61*.*7–90*.*3)*	*17*.*4 (17*.*4–56*.*9)* ^*Δ*^
Non-dominant side**	*6*.*9* _*a*_ *(4*.*7–9*.*9)*	*10*.*3* _*a*, *b*_ *(7*.*7–14*.*8)*	*37*.*1* _*b*, *c*_ *(22*.*9–63*.*5)*	*73*.*6* _*c*_ *(58*.*8–83*.*7)*	*17*.*4 (7*.*9–58*.*2)*

Values are median (IQR) and overall population size is n = 81 unless otherwise indicated

^a^ One sitter patient with SMA type 2 could not be evaluated by Myotools due to a starting position not compatible with the tests

** p ≤ 0.001

Wilcoxon signed rank test– ^Δ Δ^ p ≤ 0.001, ^Δ^ p ≤ 0. 05

_a, b, c_ Subscript letters represent Post-hoc tests results. In a row, a same subscript letter indicates subset of categories (non-sitter SMA type 2, sitter SMA type 2, non-ambulant SMA type 3 and ambulant SMA type 3) which do not differ significantly from each other at level 0.05.

**Timed tests**

Lower limb strength and functional endurance/fatigue were evaluated with timed-tests in 10 ambulant patients. The median distance walked during the 6MWT was 369 (255–451) meters which is 54.1 (34.9–63.5) % of the predicted distance for age [40, 41]. The predicted 6MWT distance according to the first 25 meters’ velocity was 386.2 (106.9) which was significantly higher (p = 0.013). The median time in seconds to complete the 10MWT was 5.3 (4.5–9.1), to climb 4 standard stairs was 3.9 (4.8–11.6), to descend stairs was 2.7 (3.1–12.1) and finally to rise from floor was 8.5 (2.0–5.3).

#### Muscle atrophy

**Neurophysiological condition**

In all the four nerve-muscle groups studied, CMAP amplitude and area differed significantly between the four groups. The lowest values were observed in non-sitter patients with SMA Type 2, the intermediate values in sitter patients with SMA Type 2 and non-ambulant patients with SMA Type 3, and the highest values in ambulant patients. These differences were especially prominent in the accessory-*trapezius* and peroneal-*tibialis anterior* (Table 5). Likewise, a higher decrement in amplitude was measured in patients with SMA Type 2 in the proximal accessory-*trapezius*. However, the decrement in area was significantly different between the four functional groups only in the more distal muscle-nerve group studied, the peroneal *tibialis anterior*, with the highest decrement in the youngest sitter SMA Type 2 group.

**Table 5:** Electrophysiologic assessment of SMA type 2 and 3 patients.

	SMA type 2			SMA type 3		Overall (n = 63)
	Non-Sitter (n = 12)		Sitter (n = 24)	Non-Ambulant (n = 8)	Ambulant (n = 16)	
**Ulnar-*ADM***						
*Exploitable results (%)*	*87*		*92*	*100*	*100*	*97*
*1*^*st*^ *CMAP Amplitude (mV)* **	*0*.*9* _*a*_ *(0*.*6–2*.*0)*		*1*.*1* _*a*_ *(0*.*7–1*.*7)*	*7*.*0* _*b*_ *(5*.*7–9*.*0)*	*6*.*8* _*b*_ *(4*.*9–10*.*2)*	*2*.*0 (0*.*8–6*.*6)*
*Amplitude Decrement (%)* ^*1*^	*-4*.*1 (-5*.*0–0*.*0)*		*-8*.*0 (-12*.*4–0*.*9)*	*-4*.*2 (-7*.*7- -2*.*1)*	*-2*.*2 (-7*.*3–0*.*7)*	*-4*.*1 (-8*.*0–0*.*3)*
*Pathologic Amplitude Decrement (n)*	*2* _*a*_		*9* _*a*_	*0* _*a*_	*2* _*a*_	*13*
*Area Decrement (%)*	*-6*.*0 (-14*.*7–0*.*3)*		*-9*.*2 (-18*.*2- -2*.*4)*	*-7*.*6 (-9*.*5- -1*.*2)*	*-8*.*0 (-11*.*7- -3*.*5)*	*-8*.*0 (-14*.*2- -2*.*0)*
**Accessory-*trapezius***						
*Exploitable results (%)*	*87*		*92*	*88*	*100*	*92*
*1*^*st*^ *CMAP Amplitude (mV)* **	*0*.*4* _*a*_ *(0*.*2–1*.*2)*		*0*.*7* _*a*_ *(0*.*5–1*.*4)*	*4*.*0* _*b*_ *(3*.*1–6*.*4)*	*5*.*2* _*b*_ *(3*.*5–8*.*2)*	*1*.*5 (0*.*5–4*.*2)*
*Amplitude Decrement (%)*	*-12*.*2 (-26*.*7- -9*.*0))*		*-18*.*0 (-36*.*0- -5*.*0)*	*-4*.*3 (-19*.*3–2*.*0)*	*-6*.*5 (-8*.*8- -2*.*4)*	*-11*.*3 (-22*.*5- -3*.*4)*
Pathologic Amplitude Decrement (n)*	*9* _*a*_		*16* _*a*_	*3* _*a*, *b*_	*3* _*b*_	*31*
*Area Decrement (%)*	*-16*.*1 (-38*.*5- -9*.*2)*		*-22*.*0 (-43*.*0- -5*.*3)*	*-11*.*2 (- 29*.*0- -4*.*0)*	*-8*.*4 (-19*.*0- -3*.*6)*	*-12*.*8 (-29*.*3- -5*.*0)*
**Radial-*anconeus***						
*Exploitable results (%)*	*73*		*62*	*88*	*94*	*73*
*1*^*st*^ *CMAP Amplitude (mV)* **	*0*.*5* _*a*_ *(0*.*4–1*.*2)*		*0*.*8* _*a*_ *(0*.*4–1*.*2)*	*3*.*1* _*b*_ *(1*.*5–7*.*4)*	*2*.*9* _*b*_ *(2*.*3–4*.*4)*	*1*.*5 (0*.*7–3*.*1)*
*Amplitude Decrement (%)*	*-0*.*1 (-17*.*2–4*.*2)*		*-6*.*0 (-28*.*6- -3*.*0)*	*-9*.*0 (-14*.*7–4*.*6)*	*-6*.*0 (-8*.*6- -3*.*0)*	*-6*.*0 (-16*.*0–0*.*0)*
*Pathologic Amplitude Decrement (n)*	*3* _*a*_		*7* _*a*_	*3* _*a*_	*2* _*a*_	*15*
*Area Decrement (%)*	*-10*.*0 (-18*.*0–2*.*5)*		*-18*.*0 (-30*.*8- -4*.*5)*	*-12*.*5 (-21*.*7- -1*.*0)*	*-7*.*5 (-12*.*0- -1*.*0)*	*-11*.*0 (-20*.*4- -1*.*0)*
**Peroneal-*tibialis anterior***						
*Exploitable results (%)*	*40*		*77*	*100*	*94*	*73*
*1*^*st*^ *CMAP Amplitude (mV)* **	*0*.*5* _*a*_ *(0*.*3–1*.*0)*		*0*.*8* _*a*, *b*_ *(0*.*5–1*.*2)*	*2*.*4* _*b*, *c*_ *(1*.*7–3*.*9)*	*4*.*5* _*c*_ *(2*.*4–5*.*9)*	*1*.*5 (0*.*6–3*.*9)*
*Amplitude Decrement (%)* *	*1*.*0 (-11*.*5–15*.*0)*		*-7*.*0 (-25*.*0- -2*.*3)*	*-6*.*5 (-9*.*5- -1*.*5)*	*-4*.*0 (-6*.*0- -2*.*0)*	*-5*.*5 (-10*.*0- -1*.*8)*
*Pathologic Amplitude Decrement (n)*	*1* _*a*_		*9* _*a*_	*2* _*a*_	*1* _*a*_	*13*
*Area Decrement (%)* *	*2*.*4* _*a*_ *(-13*.*0–14*.*5)*		*-21*.*4* _*b*_ *(-31*.*7- -6*.*2)*	*-7*.*8* _*a*, *b*_ *(-13*.*7- -3*.*8)*	*-6*.*0* _*a*, *b*_ *(-11*.*0- -5*.*0)*	*-8*.*7 (-20*.*2- -4*.*4)*

Abbreviations: *ADM*: *Abductor Digiti Minimi*; CMAP: Compound Muscle Action Potentials

Values are median (IQR) and overall population size is n = 63 unless otherwise indicated

^1^ The decrement is considered as pathologic when higher than 10%

* p ≤ 0.05

** p ≤ 0.001

_a, b, c_ Subscript letters represent Post-hoc tests results. In a row, a same subscript letter indicates a subset of categories (SMA type 2, non-ambulant SMA type 3 and ambulant SMA type 3) which do not differ significantly from each other at level 0.05.

**Muscle imaging**

**Fig 3:**
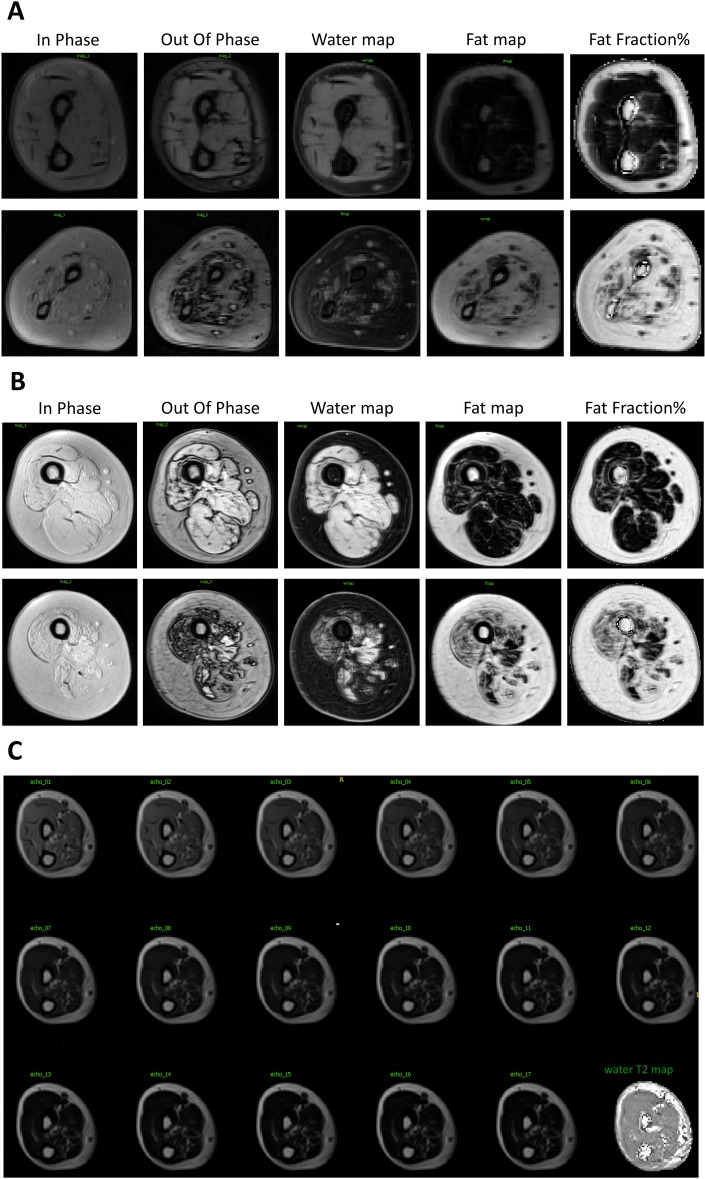
Example of MRI images of patients with SMA type 2 and 3. A. Example of Dixon images and water T2 map in mildly (upper panel; non-ambulant SMA type 3) and more severely (lower panel; non-sitter SMA type 2) infiltrated patient forearms; B. Example of Dixon images and water T2 map in mildly (upper panel) and more severely (lower panel) infiltrated ambulant patient thighs; C. Example of series of spin echo images at increasing TEs (10 ms steps) in the forearm of a patient and the water T2 map reconstructed from this series using the tri-exp fitting method.

In the four upper limb muscle groups studied by MRI, CSA and more significantly C-CSA were different between the four patient groups, with the lowest average in the youngest sitter SMA Type 2 group and the highest average in the oldest non-ambulant SMA Type 3 group (Table 6 and Fig 3). Interestingly, the fat fraction ranges in forearm *Flexors* and *Extensors* were higher in all patient groups (20–50%) than in previously published healthy controls (>5%) [37, 38]. The highest fat fraction was found in sitter patients with SMA Type 2 and the lowest fat fraction in ambulant patients in the *Biceps Brachii* and the forearm *Flexors*. Conversely, the *Triceps* showed the greatest difference between the four groups in water T2 mean and percentage of muscle volume with abnormal water T2. These results were also most pronounced in sitter patients with SMA Type 2. Water T2 heterogeneity was significantly different between the four groups only in the forearm *Extensors*. Although the forearm *Flexors* and *Extensors* water T2 values and T2 heterogeneity were within normal ranges, the percentage of voxels with abnormal T2 was higher compared to healthy controls.

**Table 6:** Muscle MRI imaging of SMA type 2 and 3 patients.

		SMA type 2		SMA type 3			Overall (n = 20) ^1^	Healthy controls
		Non-Sitter (n = 2)	Sitter (n = 6)	Non-Ambulant (n = 4)		Ambulant (n = 8)		
Arm								
	*Biceps Brachii*							
	*CSA (cm*^*2*^)*	*317* _*a*, *b*_ *(-)*	*185* _*a*_ *(172–224)*	*599* _*b*_ *(557–645)*		*300* _*a*, *b*_ *(245–1052)*	*281 (201–574)*	
	*C-CSA (cm*^*2*^)*	*214* _*a*, *b*_ *(-)*	*160* _*a*_ *(131–179)*	*536* _*b*_ *(492–598)*		*257* _*a*, *b*_ *(215–583)*	*249 (163–502)*	
	Fat Fraction (%)*	*45*.*9* _*a*_ *(-)*	*24*.*5* _*a*, *b*_ *(19*.*1–32*.*2)*	*15*.*5* _*a*, *b*_ *(10*.*0–17*.*9)*		*11*.*9* _*b*_ *(8*.*2–13*.*4)*	*18*.*5 (11*.*9–28*.*3)*	
	*Water T2 mean (ms)*	*34*.*5 (-)*	*35*.*2 (27*.*8–36*.*9)*	*36*.*1 (35*.*4–38*.*0)*		*36*.*4 (32*.*8–37*.*4)*	*35*.*7 (33*.*4–36*.*7)*	
	*Abnormal T2 (%)*	*25*.*8 (-)*	*25*.*0 (8*.*7–28*.*8)*	*18*.*1 (9*.*5–34*.*1)*		*13*.*7 (4*.*5–27*.*2)*	*19*.*6 (8*.*2–28*.*1)*	
	*Water T2 heterogeneity (CV)*	*0*.*098 (-)*	*0*.*126 (0*.*107–0*.*152)*	*0*.*094 (0*.*082–0*.*114)*		*0*.*108 (0*.*082–0*.*132)*	*0*.*108 (0*.*086–0*.*130)*	
	*Triceps*							
	*CSA (cm*^*2*^)*	*339* _*a*, *b*_ *(-)*	*180* _*a*_ *(124–201)*	*632* _*b*_ *(517–707)*		*308* _*b*_ *(236–308)*	*279 (201–548)*	
	*C-CSA (cm*^*2*^)*	*168* _*a*, *b*_ *(-)*	*116* _*a*_ *(96–177)*	*522* _*b*_ *(415–608)*		*229* _*a*, *b*_ *(217–462)*	*220 (151–426)*	
	*Fat Fraction (%)*	*70*.*9 (-)*	*19*.*8 (12*.*9–42*.*8)*	*20*.*3 (13*.*6–36*.*5)*		*12*.*9 (11*.*6–19*.*9)*	*18*.*1 (12*.*9–39*.*0)*	
	Water T2 mean (ms)*	*36*.*2* _*a*, *b*_ *(-)*	*34*.*0* _*a*_ *(32*.*8–36*.*4)*	*39*.*2*_*b*_ *(37*.*6–40*.*5)*		*37*.*3* _*a*, *b*_ *(36*.*7–38*.*9)*	*37*.*2 (34*.*4–38*.*8)*	
	Abnormal T2 (%)*	*26*.*0* _*a*, *b*_ *(-)*	*10*.*4* _*a*_ *(8*.*7–23*.*1)*	*40*.*8* _*b*_ *(34*.*6–60*.*4)*		*29*.*8* _*a*, *b*_ *(18*.*0–47*.*6)*	*29*.*8 (17*.*7–37*.*0)*	
	*Water T2 heterogeneity (CV)*	*0*.*130 (-)*	*0*.*125 (0*.*092–0*.*150)*	*0*.*080 (0*.*070–0*.*114)*		*0*.*111 (0*.*079–0*.*111)*	*0*.*111 (0*.*084–0*.*129)*	
Forearm								
	*Flexor* group							*n = 12*^*2*^
	*CSA (cm*^*2*^)*	*531* _*a*, *b*_ *(-)*	*204* _*a*_ *(177–240)*	*519* _*b*_ *(483–611)*		*349* _*b*_ *(319–860)*	*349 (229–554)*	
	*C-CSA (cm*^*2*^)*	*183* _*a*, *b*_ *(-)*	*143* _*a*_ *(119–200)*	*435* _*b*_ *(392–513)*		*325* _*a*, *b*_ *(271–499)*	*269 (156–398)*	
	Fat Fraction (%)*	*77*.*9* _*a*_ *(-)*	*36*.*1* _*a*, *b*_ *(28*.*5–62*.*1)*	*33*.*3* _*a*, *b*_ *(23*.*3–36*.*8)*		*17*.*1* _*b*_ *(12*.*7–23*.*6)*	*30*.*4 (19*.*8–50*.*9)*	*3*.*7 (1*.*1)*
	*Water T2 mean (ms)*	*34*.*6 (-)*	*36*.*1 (33*.*8–37*.*2)*	*37*.*2 (36*.*5–38*.*0)*		*36*.*9 (35*.*2–37*.*7)*	*36*.*5 (35*.*0–37*.*7)*	*35*.*1 (0*.*7)*
	*Abnormal T2 (%)*	*10*.*0 (-)*	*20*.*1 (14*.*2–28*.*5)*	*24*.*5 (16*.*5–32*.*2)*		*20*.*5 (15*.*4–30*.*2)*	*20*.*0 (15*.*4–30*.*2)*	*10*.*0 (5*.*1)*
	*Water T2 heterogeneity (CV)*	*0*.*151 (-)*	*0*.*138 (0*.*106–0*.*158)*	*0*.*098 (0*.*089–0*.*108)*		*0*.*104 (0*.*077–0*.*126)*	*0*.*115 (0*.*091–0*.*115)*	*0*.*10 (0*.*02)*
	*Extensor* group							*n = 12*^*2*^
	*CSA (cm*^*2*^)*	*229* _*a*, *b*_ *(-)*	*120* _*a*_ *(94–153)*	*453* _*b*_ *(319–474)*		*159* _*a*, *b*_ *(137–772)*	*203 (133–432)*	
	*C-CSA (cm*^*2*^)*	*101* _*a*, *b*_ *(-)*	*93* _*a*_ *(65–130)*	*397* _*b*_ *(263–412)*		*142* _*a*, *b*_ *(120–402)*	*130 (95–325)*	
	Fat Fraction (%)*	*64*.*6 (-)*	*26*.*5 (9*.*3–38*.*3)*	*12*.*8 (7*.*6–16*.*9)*		*10*.*2 (6*.*3–12*.*9)*	*12*.*8 (9*.*2–28*.*2)*	*4*.*5 (2*.*8)*
	*Water T2 mean (ms)*	*34*.*5 (-)*	*35*.*0 (33*.*8–36*.*1)*	*36*.*6 (35*.*6–38*.*7)*		*36*.*2 (34*.*2–36*.*7)*	*35*.*5 (34*.*2–36*.*7)*	*34*.*1 (1*.*9)*
	*Abnormal T2 (%)*	*10*.*4 (-)*	*15*.*3 (9*.*9–26*.*1)*	*12*.*4 (6*.*1–35*.*32)*		*11*.*1 (8*.*7–24*.*8)*	*12*.*2 (9*.*2–23*.*4)*	*6*.*7 (6*.*9)*
	Water T2 heterogeneity (CV)*	*0*.*145 (-)*	*0*.*136 (0*.*111–0*.*147)*	*0*.*083 (0*.*069–0*.*105)*		*0*.*108 (0*.*081–0*.*127)*	*0*.*116 (0*.*088–0*.*137)*	*0*.*10 (0*.*02)*
Thigh ^3^		*n = 0*	*n = 1* ^*3*^	*n = 0*		*n = 7* ^*3*^	*n = 8*	
	*Quadriceps*							*n = 33* ^*4*^
	*CSA (cm*^*2*^*)*		*626*			*840 (653–2029)*	*838 (633–1977)*	
	*C-CSA (cm*^*2*^*)*		*507*			*774 (543–1270)*	*772 (516–1159)*	
	*Fat Fraction (%)*		*31*.*9*			*27*.*3 (19*.*5–79*.*4)*	*29*.*1 (19*.*9–67*.*3)*	*1*.*8 (0*.*4)*
	*Water T2 mean (ms)*		*41*.*3*			*39*.*8 (35*.*3–41*.*0)*	*39*.*9 (36*.*0–41*.*2)*	*35*.*0 (0*.*9)*
	*Abnormal T2 (%)*		*68*.*1*			*53*.*5 (16*.*5–66*.*1)*	*55*.*7 (21*.*6–67*.*6)*	*4*.*0 (4*.*5)*
	*Water T2 heterogeneity (CV)*		*0*.*094*			*0*.*095 (0*.*085–0*.*103)*	*0*.*095 (0*.*087–0*.*101)*	*0*.*058 (0*.*009)*
	*Hamstring*							
	*CSA (cm*^*2*^*)*		*344*			*560 (462–225)*	*547 (435–2056)*	
	*C-CSA (cm*^*2*^*)*		*282*			*524 (409–1987)*	*500 (372–1734)*	
	*Biceps Femoris*							
	*Fat Fraction (%)*		*18*.*9*			*15*.*5 (10*.*2–27*.*5)*	*17*.*0 (10*.*6–25*.*3)*	
	*Water T2 mean (ms)*		*38*.*4*			*36*.*4 (35*.*0–38*.*7)*	*36*.*9 (35*.*2–38*.*6)*	
	*Abnormal T2 (%)*		*42*.*0*			*18*.*1 (7*.*6–40*.*7)*	*25*.*5 (8*.*4–41*.*7)*	
	*Water T2 heterogeneity (CV)*		*0*.*068*			*0*.*091 (0*.*065–0*.*125)*	*0*.*087 (0*.*065–0*.*117)*	
	*Semi Membranosus*							
	*Fat Fraction (%)*		*14*.*4*			*12*.*8 (8*.*0–27*.*5)*	*13*.*6 (8*.*6–25*.*3)*	
	*Water T2 mean (ms)*		*37*.*3*			*36*.*0 (35*.*5–36*.*8)*	*36*.*1 (35*.*6–36*.*8)*	
	*Abnormal T2 (%)*		*32*.*4*			*15*.*9 (10*.*7–28*.*2)*	*19*.*0 (11*.*4–29*.*1)*	
	*Water T2 heterogeneity (CV)*		*0*.*095*			*0*.*103 (0*.*098–0*.*110)*	*0*.*103 (0*.*095–0*.*108)*	
	*Semi Tendinosus*							
	*Fat Fraction (%)*		*21*.*0*			*20*.*1 (10*.*6–58*.*9)*	*27*.*8 (11*.*2–49*.*5)*	
	*Water T2 mean (ms)*		*34*.*4*			*35*.*1 (34*.*3–35*.*5)*	*35*.*0 (34*.*3–35*.*4)*	
	*Abnormal T2 (%)*		*13*.*1*			*5*.*6 (3*.*8–14*.*7)*	*7*.*5 (4*.*0–14*.*3)*	
	*Water T2 heterogeneity (CV)*		*0*.*111*			*0*.*089 (0*.*064–0*.*099)*	*0*.*093 (0*.*070–0*.*108)*	
Leg ^3^		*n = 0*	*n = 1* ^*3*^	*n = 0*		*n = 7* ^*3*^	*n = 8*	
	*Extensor*							
	*CSA (cm*^*2*^*)*		*125*			*203 (178–872)*	*197 (175–810)*	
	*C-CSA (cm*^*2*^*)*		*102*			*186 (168–709)*	*185 (163–655)*	
	*Peroneus Lateralis*							
	*CSA (cm*^*2*^*)*		*88*			*138 (89–397)*	*119 (88–383)*	
	*C-CSA (cm*^*2*^*)*		*61*			*122 (82–316)*	*108 (70–314)*	
	*Fat Fraction (%)*		*57*.*0*			*16*.*6 (14*.*1–17*.*0)*	*16*.*8 (14*.*7–29*.*3)*	
	*Water T2 mean (ms)*		*36*.*2*			*35*.*0 (33*.*7–36*.*9)*	*35*.*4 (33*.*8–36*.*7)*	
	*Abnormal T2 (%)*		*2*.*3*			*5*.*5 (2*.*7–20*.*4)*	*4*.*1 (2*.*3–19*.*0)*	
	*Water T2 heterogeneity (CV)*		*0*.*046*			*0*.*068 (0*.*044–0*.*086)*	*0*.*065 (0*.*044–0*.*082)*	
	*Tibialis Anterior*							
	*Fat Fraction (%)*		*40*.*5*			*10*.*3 (6*.*5–17*.*6)*	*13*.*5 (6*.*9–34*.*8)*	
	*Water T2 mean (ms)*		*38*.*0*			*35*.*0 (34*.*4–37*.*2)*	*35*.*3 (34*.*5–37*.*7)*	
	*Abnormal T2 (%)*		*46*.			*6*.*4 (2*.*1–22*.*7)*	*11*.*3 (2*.*7–29*.*4)*	
	*Water T2 heterogeneity (CV)*		*0*.*080*			*0*.*080 (0*.*059–0*.*082)*	*0*.*080 (0*.*064–0*.*082)*	
	*Triceps Surae*							
	*CSA (cm*^*2*^*)*		*192*			*768 (607–2774)*	*735 (554–2581)*	
	*C-CSA (cm*^*2*^*)*		*122*			*717 (572–2624)*	*697 (516–2351)*	
	*Fat Fraction (%)*		*67*.*4*			*13*.*6 (12*.*7–16*.*6)*	*14*.*1 (12*.*7–42*.*0)*	
	*Water T2 mean (ms)*		*34*.*8*			*35*.*5 (34*.*9–37*.*0)*	*35*.*7 (34*.*9–36*.*8)*	
	*Abnormal T2 (%)*		*14*.*6*			*16*.*0 (8*.*3–20*.*7)*	*15*.*3 (9*.*5–20*.*7)*	
	*Water T2 heterogeneity (CV)*		*0*.*112*			*0*.*062 (0*.*055–0*.*0121)*	*0*.*062 (0*.*056–0*.*119)*	

Abbreviations: CSA: Cross Section Area; C-CSA: Contractile-Cross Section Area; Abnormal T2: percentage of voxels with abnormal T2 (>39ms); SD: Standard Deviation

Values are median (IQR) and overall population size is n = 19 unless otherwise indicated

^1^ Twenty patients were evaluated for MRI, seventeen during baseline visit and three during month-6 visit

^2^ Forearm *Flexors* and *Extensors* fat fraction, water T2 mean, abnormal T2 and water T2 heterogeneity were previously measured in n = 12 healthy controls aged 7–18 years [38]. Values are mean (SD)

^3^ Lower limb muscles were assessed in seven ambulant patients with SMA type 3 and one sitter patient with SMA type 2. This latter patient was evaluated although the initial protocol imaging design did not include non-ambulant patients in lower limb MRI acquisition. Since then it has been amended in order to be able to capture these data

^4^ *Quadriceps* fat fraction, water T2 mean, abnormal T2 and water T2 heterogeneity were previously measured in n = 33 healthy boys aged 19–27 years[37]. Values are mean (SD).

* p ≤ 0.05

_a, b_ Subscript letters represent Post-hoc tests results. In a row, a same subscript letter indicates subset of categories (non-sitter SMA type 2, sitter SMA type 2, non-ambulant SMA type 3 and ambulant SMA type 3) which do not differ significantly from each other at level 0.05.

Lower limb muscles were assessed in seven ambulant patients with SMA Type 3 and one sitter patient with SMA Type 2 (Table 6). All muscles showed significant levels of fatty degenerative changes (15–40% in SMA versus <5% in the quadriceps of healthy subjects evaluated with the same technique). Water T2 was frequently within normal range but T2 heterogeneity was slightly higher in the study sample than in healthy subjects. Similar to the results seen in the upper extremity *Flexors* and *Extensors*, the percentage volume with abnormal T2 in quadriceps of patients with SMA was higher than in healthy controls.

#### Molecular biomarkers

Two patients were identified with one *SMN1* copy and expressed measurable levels of *SMN1* mRNA (Tables 1 and 7). This was supported by prior gene sequencing at diagnosis showing that both patients had a loss of function mutation in the *SMN1* allele. The number of *SMN2* copies was significantly different between SMA Types (p = 0.004), 92% of patients with SMA Type 2 had 3 copies whereas 28% of patients with SMA Type 3 had 4 copies (Table 1). As expected, *SMN2*, *SMND7* or their mRNA blood ratios were not significantly different between SMA Types or functional groups. Additionally, SMN protein levels in blood were similar between the four groups (Table 7).

**Table 7:** Molecular SMA biomarkers.

	SMA type 2		SMA type 3		Overall
	Non-Sitter	Sitter	Non-Ambulant	Ambulant	
***SMN1* mRNA level** ^**1**^^,^ ^**2**^ **(fold changes)**	*n = 0*	*n = 1*	*n = 0*	*n = 1*	*n = 2*
	0 (-)	0.11 (-)	0 (-)	0.30 (-)	*0*.*20 (0*.*08–0*.*22)*
***SMN2* mRNA level** ^**1**^^,^ ^**3**^ **(fold changes)**	*n = 14*	*n = 26*	*n = 7*	*n = 13*	*n = 60*
	0.76 (0.37–1.06)	1.16 (0.51–1.26)	0.62 (0.28–1.30)	1.06 (0.54–1.15)	0.96 (0.45–1.20)
***SMND7* mRNA level** ^**1**^^,^ ^**3**^ **(fold changes)**	*n = 14*	*n = 26*	*n = 7*	*n = 13*	*n = 60*
	0.67 (0.37–0.75)	0.79 (0.41–0.88)	0.49 (0.40–0.80)	0.72 (0.43–0.78)	0.69 (0.41–0.81)
**SMN protein level** ^**1**^^,^ ^**4**^ **(pg/mL)**	*n = 18*	*n = 33*	*n = 9*	*n = 18*	*n = 78*
	2832 (2452–3743)	3122 (2883–3810)	3316 (2445–3852)	3597 (2930–4292)	*3187 (2715–3858)*

Values are median (IQR)

^1^ Baseline samples could not be collected for one non-sitter SMA type 2 patient due to lack of patient compliance during the procedure (2 years old patient)

^2^ *SMN1* mRNA level have been only detected in the two patients with 1 *SMN1* copy (Table 1)

^3^ Baseline RNA results of 20 patients were excluded after quality control (missing results for SMN2, SMND7 or RG PCR for some samples or results out of assay specifications). Values are reported as described in [7]

^4^ Two baseline SMN protein samples could not be collected for two patients due to lack of collected blood (one sitter SMA type 2 and one ambulant SMA type 3).

#### Cross-sectional correlations between clinical parameters

**Fig 4:**
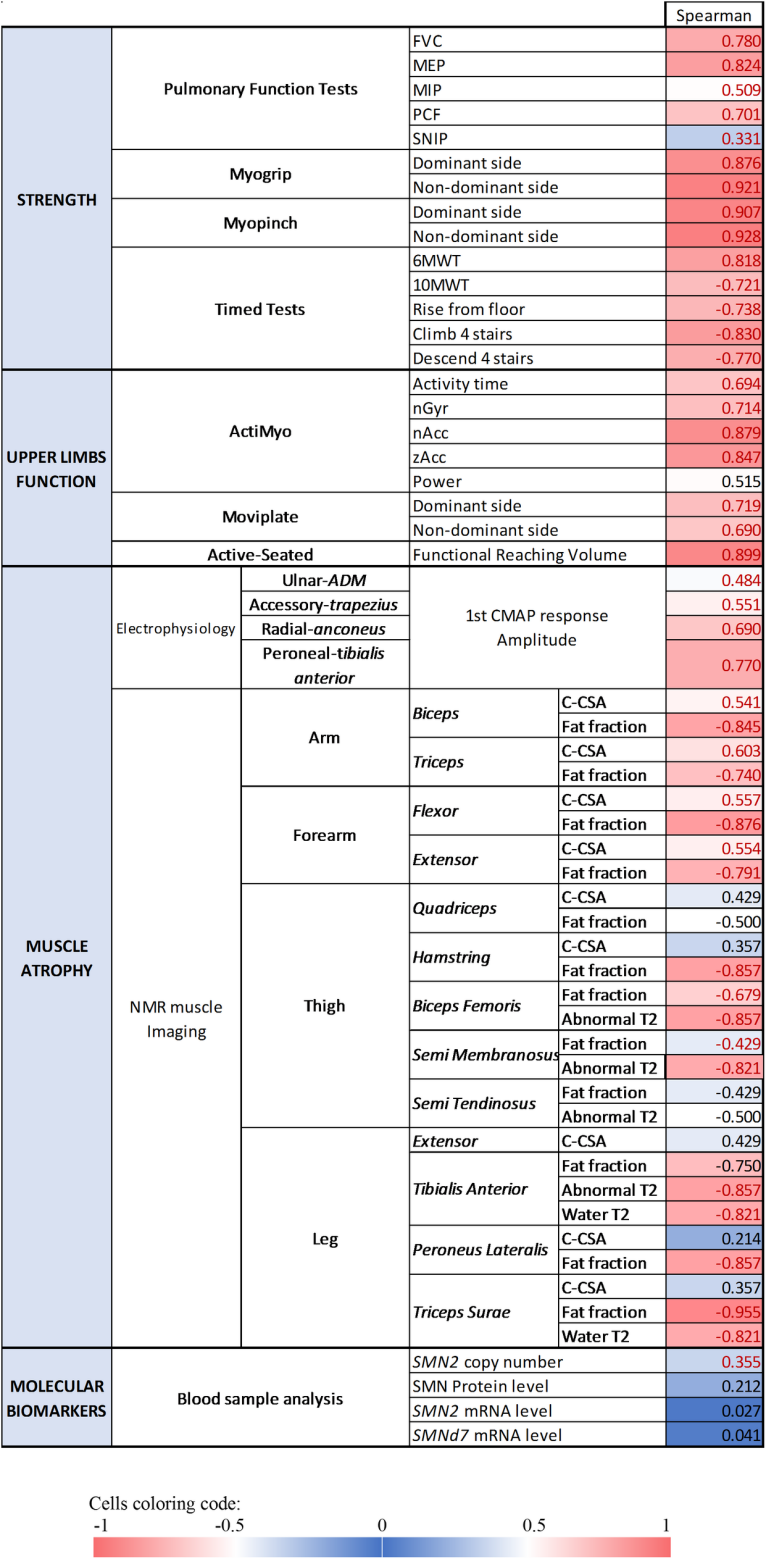
Correlation matrix between motor function (MFM) total score and other outcome measures. Abbreviations: FVC: Forced Vital Capacity; MEP: Maximum Expiratory Pressure; MIP: Maximum Inspiratory Pressure; PCF: Peak Cough Flow; SNIP: Sniff Nasal Inspiratory Pressure; 6MWT: 6-Minutes-Walk Test; 10MWT: 10-Meters-Walk Test; nGyr: norm of the angular velocity of the wrist; nAcc: norm of the acceleration of the wrist; zAcc: vertical acceleration of the wrist; ADM: *Abductor Digiti Minimi*; CMAP: Compound Muscle Action Potentials; C-CSA: Contractile-Cross Section Area; Abnormal T2: percentage of voxels with abnormal T2 Values in red: Spearman’s correlation test with p ≤ 0.05.

We evaluated the relationship between the MFM total score (MFM-20 or MFM-32), which is performed in all patients at baseline, and other measured outcomes (Fig 4). The lowest correlations were observed with the studied molecular biomarkers, which show that *SMN2* copy number, *SMN2* and *SMND7* mRNA levels as well as SMN protein levels are only weakly correlated with motor function. In contrast, the highest correlations were observed with strength and upper limb function measures (PFT, MyoSet, Timed Tests, ActiMyo^®^, Active-seated). We also correlated the MFM total scores with biomarkers of muscle atrophy, neurophysiology and muscle imaging variables. Indeed, CMAP nicely correlated with MFM for the four studied nerve-muscle groups. Lastly, the highest correlations between MFM and MRI variables were observed with fat fraction in arm and forearm muscles as well as in the posterior thigh muscles, and with the percentage of muscle volume with abnormal T2 in *Biceps Femoris*, *Semi Membranosus* and *Tibialis Anterior*.

## Discussion

We demonstrated the feasibility of gathering consistent data from numerous and sometimes complex assessments in a multicenter study of SMA. Most outcome measures discriminated between non-sitter patients with SMA Type 2, sitter patients with SMA Type 2, non-ambulant patients with SMA Type 3 and ambulant patients and strongly correlated with MFM scores.

The clinical features of the current population were similar to those previously described in patients with SMA Type 2 and 3, particularly the age first symptoms appeared, nutrition/gastrostomy complications and ventilation status [9, 42]. Cognitive impairments were not reported in any of the study subjects, confirming that this disease is not an obstacle to education. As expected, our results confirmed the psychomotor developmental delay as well as the higher and earlier functional degradation in the more affected patients with SMA Type 2. This included a higher incidence of scoliosis in patients with SMA Type 2 than in Type 3 and a higher incidence of arthrodesis in the non-sitter patients than in sitter individuals [43]. Comparable to recent reports [44], almost all patients received physiotherapy, with SMA Type 2 sitter patients reporting a greater frequency of sessions than ambulant patients. In the present cohort, the extensive panel of supportive devices used for nutrition and respiratory management as well as the frequent orthotic use reflects the adapting strategies as standard of care for the wide-range of phenotypes [45].

Motor function as assessed by MFM or other validated scales, such as the Revised Hammersmith Scale, is the primary outcome of choice in planned, ongoing or completed clinical trials in SMA [26, 46, 47]. Motor function scales have been previously shown to discriminate between SMA Type 2 and 3 [19, 42], and ambulant versus non-ambulant patients [48]. In the present cohort, we chose to use the MFM scale since it can be performed in patients as young as 2 years old irrespective of ambulant status and disease type [26]. It captures different patterns of weakness (proximal/distal) and motor function changes such as axial motor ability and upper limb performances even in very weak patients [11, 49]. Our cross-sectional analysis confirmed that MFM discriminates SMA Types as well as sitter and ambulant status, with no significant overlap between SMA Types. As previously described, we observed a slow decrease of MFM with age particularly affecting MFM sub-scores D2 in patients with SMA Type 2 [25]. Additionally, using scales adapted to patient age (MFM-20 from 2 to 5 and MFM-32 after 6 years old) avoided the threshold effect earlier described with MFM-20 in non-ambulant patients [49].

In addition to the MFM, upper extremity strength and function assessments are also key targets for outcome measures in SMA. In this study we evaluated three novel outcome measures to quantify upper extremity strength and function. The MyoSet devices measured grip and pinch strength and the capacity to repeatedly flex and extend the hand and fingers [19], the Active-Seated system measured upper extremity workspace volume and trunk control with a gaming interface [13] and the ActiMyo^®^ continuous monitored linear and rotational arm movements and velocity in the home [14]. We hypothesized that this combination would capture a complete picture of upper limb strength and function.

We previously demonstrated that the MyoGrip and MyoPinch are sensitive dynamometers that assess upper limb strength in non-ambulant SMA Type 2 and 3, even in the weakest ones [19]. The Active-Seated uses a 60-second video game to quantify workspace volume and the ability to lean. Our results confirmed that the MyoSet devices and Active-seated discriminate patients with SMA Type 2 from Type 3 and strongly correlated with MFM. Remarkably, these tests revealed varying levels of strength and function within the non-ambulant SMA Type 3 population, yet these differences were indiscernible from the MFM score alone (59.37 ±1.87). This suggests that quantitative upper limb distal strength and function could add granularity to the MFM functional scale. Altogether, this demonstrates that combining these tests with MFM optimizes motor function assessment, irrespective of the disease type or ambulant status.

The innovative ActiMyo^®^ device uses state of the art magneto-inertial sensors to record upper limb movements in patient everyday life. It has been shown to be an effective feasible measure in a pilot study in very weak non-ambulant DMD patients [14]. The current data shows that selected variables, representative of patient upper limb activity in real life significantly correlated with MFM scores, but also with other variables such as PFT and MyoGrip scores. The strongest correlations in this study were observed with the wrist vertical acceleration, and the median wrist angular velocity was decreased in the sitter patients with SMA Type 2 when compared to the non-sitter individuals. Both variables are clinically meaningful as they represent the vertical lifting and lowering movements of wrist and forearm performed during daily activities. In addition to evaluating disease progression in the different functional domains, it also has the potential to assess fatigue and loss of endurance during daily activities, both of which are of utmost interest for quality of life impairment measurement.

We also demonstrated that PFT is well correlated with the MFM. Our baseline results showed that FVC, MEP and PCF significantly separated the four functional groups. The lowest values were found in non-sitter patients with SMA Type 2 who had the most impaired lung function, whereas ambulant patients had close to normal values, which is in line with previous studies using FVC [8, 19, 35, 50]. None of the measured parameters significantly differed between non-ambulant SMA Type 3 patients and ambulant patients which could be due to the small sample sizes. Remarkably, all four parameters correlated with MFM total score, with the strongest correlations observed with FVC and MEP. In contrast with a previous report (44), SNIP displayed a high variability. In addition, SNIP failed to discriminate between the three groups and only weakly correlated with MFM. This result might be due to difficulties younger children may have had to perform inspiratory maneuvers, from incorrect follow-up procedures by physiotherapists or from technical issues such as air leaks.

Ambulatory capacity and endurance in SMA are also a central aspect of disease status. Our baseline results with ten ambulant patients showed that the distance walked during the 6MWT test and the time needed for 10MWT, climb or descend stairs were similar as previously described in other cohorts [22, 35]. The distance walked during the 6MWT by this cohort was less than 66% of the predicted distance for age and height. We reproduced the decreased gait velocity during the 6MWT [34], which highlights the contribution of increased fatigability and reduced leg strength to the disease phenotype [51]. Similarly, the TRF was ten times higher than in comparable healthy controls [52]. We found a strong positive correlation between the 6MWT tests and MFM total score [12], whereas the other four tests were negatively correlated with MFM. Altogether, this confirms that timed tests provide in ambulant patients a complementary measure of both strength and fatigue in close connection with motor function and therefore disease severity.

CMAP amplitude is a relevant indicator of motor neuron health and denervation severity and is therefore lower in patients with SMA than healthy controls, primarily in the most studied ulnar-*ADM* nerve-muscle group [10, 53]. Our baseline results are in complete agreement with previous reports [54, 55], showing reduced CMAP amplitude and area in the four nerve–muscle groups studied, which significantly discriminated the four sub-groups. The strong correlation between CMAP and the MFM total score is consistent with the correlations already described with other motor function scales [8, 56, 57] and is representative of the tight relationship between nerve integrity and motor function.

Repetitive nerve stimulation is a specific and relatively sensitive tool for detecting both presynaptic and postsynaptic dysfunction or immaturity of the neuromuscular junction. Our results were consistent with a previous study [58] showing a higher decrement in patients with SMA Type 2 than in Type 3, significant in the proximal *trapezius* but not in the distal *ADM*, consistent with a predominant proximal disease signature. We further showed that this pathological decrement in sitter patients with SMA Type 2 is also detectable in *anconeus* and *tibialis anterior*.

Muscle MRI is an emerging imaging biomarker for SMA disease monitoring. This is the first cross-sectional MRI imaging study of both upper and lower limbs muscles in a large SMA Type 2 and 3 cohort. Overall, our data showed a large fat infiltration and loss of functional tissue in arm and forearm of non-sitter patients with SMA Type 2 with the lowest C-CSA and highest fat fraction. On the contrary, the less affected ambulant patients with SMA Type 3 displayed the highest C-CSA and lowest fat fraction in arm and forearm, although fat infiltration was still higher than in healthy controls [38]. Mean T2 and abnormal T2 in the *Triceps* significantly discriminated the four functional groups, and were lower in patients with SMA Type 2, which confirms an increased disease activity in the more proximal *Extensor*. Similarly, water T2 heterogeneity values in the forearm *Extensor* were higher in non-sitter patients with SMA Type 2, indicating a water infiltration in this muscle, parallel to the fat infiltration. Finally, although collected on a very small sample, thigh and leg muscle imaging results showed a tendency of high C-CSA, mean T2 and abnormal T2 but low fat fraction and water T2 heterogeneity values in ambulant patients, with variations depending on proximal/distal level and studied muscles. Hence, this trend also indicates an increased contractile compartment and a lower fat and water infiltration in lower limbs muscles. A recent study also described reduced tissue mass and density in ambulant patient thigh muscle [12]. Altogether, upper and lower limb muscle imaging results showed that the functional muscle compartment impairment as shown by structural changes in muscle composition is related to increased disease severity. Correlating muscle imaging variables and motor function, the current results demonstrate a strong negative correlation between MFM and fat fraction in arm, forearm and leg muscles and with abnormal T2 in the *Biceps Femoris*, *Semi Membranosus* and *Tibialis Anterior* muscles, but an absent or weak correlation with CSA and C-CSA. These results broadened those of a recent publication in ambulant SMA Type 3 [12] and demonstrated that the intramuscular structural changes translate well into functional clinical changes.

Genetic analysis indicated as expected, that most patients with SMA Type 2 had two to three *SMN2* copies, whereas most Type 3 have three and four copies, which is concordant with former publications [8, 9]. The broad clinical phenotype of patients with three copies suggest that other factors account for clinical severity. *SMN2* mRNA levels were previously reported as strongly upregulated in SMA patients when compared to healthy controls [7]. In the present study, neither *SMN2* nor *SMND7* mRNA levels were related with clinical severity nor with motor function, as previously described [7, 12, 59]. Lastly, we confirmed that SMN protein expression also greatly overlaps between SMA types, with high variability irrespective of the sitter or ambulant statuses [7, 60] and was not correlated with MFM [12].

Limitations of the present study included potential confounding factors such as age (the non-ambulant Type 3 patients are older whereas the sitter patients with SMA Type 2 are younger) as well as standard of care variations between sites and countries. Fatigue is also a significant clinical aspect in patients with SMA that may interfere with some study evaluations, especially in patients older than 6 years, who performed a substantial number of successive tests.

Another limitation resides in the limited number of patients per group/subgroup. Although this study was multicenter and included up to 81 patients, subdivision by age, SMA type, ambulant or sitter status restricted the number of patients per group and did not allow subgroup analysis (comparison between SMA 3a and 3b, study of the effect of joint contractures, scoliosis, scoliosis angle and arthrodesis on motor function, etc.).

This study enrolled mainly committed patients and families, seen on a regular basis at the clinical sites. Sites reported very few refusals to participate, but these were not tracked. We didn’t experience a potential enrollment bias due to the opportunity to enroll in therapeutic trials as none were available at the beginning of this study. The enrollment number was higher than initially expected in this challenging and vulnerable population. Given that there was no direct benefit for patients in our study, this highlighted the enthusiasm of patients and families to embrace clinical research opportunities and to contribute their time and effort.

In conclusion, our cross-sectional results demonstrate that these outcome measures and biomarkers (pulmonary function and strength tests, upper limb function and abilities, compound muscle action potentials and muscle imaging) were able to differentiate non-sitter patients with SMA Type 2, sitter patients with SMA Type 2, non-ambulant patients with SMA Type 3 and ambulant patients. The majority of the measures also demonstrated a good correlation with the MFM score. The incomplete cross correlations between these tools suggest that including these additional measures in a therapeutic trial could provide a more comprehensive functional, anatomical and physiological evaluation of patients with SMA.

The objective of the longitudinal data analysis will be to identify if these outcome measures can capture even more discrete changes over time and show possible differences in disease progression between the three functional patient groups.

## Supporting information

S1 Checklist (PDF)

S1 Protocol (PDF)

S1 Statistical Analysis Plan (PDF)

S1 Table Baseline characteristics and medical history of enrolled patients. Abbreviations: bpm: breath per minute, BPAP: Bi-level positive airway pressure, CPAP: Continuous Positive Airway Pressure, IPV: Intrapulmonary Percussive Ventilation, NPV: Negative Pressure Ventilation, IPPV: Intermittent Positive Pressure Ventilation, AFO: Ankle and Foot Orthosis;
Values are median (IQR) and overall population size is n = 81 unless otherwise indicated;
* 0.001 < p ≤ 0.05; ** p ≤ 0.001;
^□^ Application conditions of the Chi-square test not fully verified (theoretical effectives ≤ 5, too small effectives); _a, b, c_ Subscript letters represent Post-hoc tests results. In a row, a same subscript letter indicates a subset of categories (non-sitter SMA type 2, sitter SMA type 2, non-ambulant SMA type 3 and ambulant SMA type 3) which column proportions do not differ significantly from each other at level 0.05.
(DOCX)

S2 Table Psychomotor development of patients with SMA type 2 and 3. Values are effectives per motor skill;
* 0.001 < p ≤ 0.05; ** p ≤ 0.001;
^□^ Application conditions of the Chi-square test not fully verified (theoretical effectives ≤ 5, too small effectives);
_a, b, c_ Subscript letters represent Post-hoc tests results. In a row, a same subscript letter indicates a subset of categories (non-sitter SMA type 2, sitter SMA type 2, non-ambulant SMA type 3 and ambulant SMA type 3) which do not differ significantly from each other at level 0.05.
(DOCX)

S1 Fig MFM scores in patients with SMA type 2 and 3. (TIF)
