# Supplementary material for: Prospective and longitudinal natural history study of patients with Type 2 and 3 spinal muscular atrophy: Baseline data NatHis-SMA study
Source: PLoS One. 2018 Jul 26;13(7):e0201004. doi: 10.1371/journal.pone.0201004 (PMC6062049; doi:10.1371/journal.pone.0201004)
Supplement: S1 Protocol — (PDF) [file pone.0201004.s002.pdf]

CLINICAL STUDY PROTOCOL

Prospective Study of the Natural History of patients with type 2 and 3 Spinal Muscular Atrophy

NatHis-SMA

VERSION n°8.0 – 24th of April 2017

Sponsor: Association Institut de Myologie
Service des Essais Cliniques et des Bases de données
GH Pitié Salpêtrière
Bâtiment Babinski
47-83 boulevard de l'hôpital
75651 Paris Cedex 13 - FRANCE
Phone: +33 1 42 16 37 86 / Fax: +33 1 42 16 37 93

Coordinating Investigator: Dr. Laurent Servais
I-Motion
Hôpital Armand Trousseau
26 Avenue du Dr Arnold Netter
75571 Paris - FRANCE
Phone: +33 1 71 73 80 39 / Fax: +33 1 44 73 65 83

Sponsor Project Referent: Mélanie ANNOUSSAMY
Email: m.annoussamy@institut-myologie.org
Phone: +33 1 71 73 83 93 / Fax: +33 1 44 73 65 83

APPROVAL PAGE OF THE CLINICAL RESEARCH PROTOCOL

Biomedical Research C*ode:* NatHis-SMA

ANSM Record Number: **IDRCB-2014-A01263-44** ***(For France only)***

Title: **"Prospective Study of the Natural History of patients with type 2 and 3 Spinal Muscular Atrophy – NatHis-SMA"**

Version N° 7.0: 24/04/2017 – Amendment #7

**Coordinating Investigator:**<br><br>**Dr Laurent Servais**<br><br>**Association Institut de Myologie**<br>Service Essais Cliniques et Bases de données<br>GH Pitié Salpêtrière - Bâtiment Babinski<br>47-83 boulevard de l'hôpital<br>75651 Paris Cedex 13 - France	Date:<br><br>Signature:
**Sponsor:**<br><br>**Marianne Perreau-Saussine**<br><br>**Association Institut de Myologie**<br>GH Pitié Salpêtrière - Bâtiment Babinski<br>47-83 boulevard de l'hôpital<br>75651 Paris Cedex 13 - France	Date:<br><br>Signature:

NB: This version correspond to the protocol and appendix sent to the Ethic Committee and the Health Authority respectively to notice and request permission.

If another version is then written following changes must repeat the circuit signatures to be always up to date versions of the active protocol

INVESTIGATOR AGREEMENT

I declare that this clinical protocol was critically reviewed and is approved by the Sponsor.

I agree to conduct this study in compliance with procedures outlined in this document according to International Conference on Harmonization (ICH) Good Clinical Practice (GCP) guidelines, the Declaration of Helsinki, and applicable regulatory requirements. This study will not be initiated without the approval of the Institutional Review Board (IRB) or Independent Ethics Committee (IEC) and the competent authority, if applicable.

I understand that any substantial changes to the protocol must be approved in writing by the IRB/IEC and the competent authority, if applicable, before it can be implemented except where necessary to eliminate immediate harm to the patient. I will provide copies of the protocol and access to all information furnished by AIM to study personnel under my supervision and will discuss this material with them to ensure they are fully informed about the study. I understand that the study may be terminated or enrollment suspended at any time by AIM with or without cause, or by me if it becomes necessary to protect the best interests of the study patients.

Investigator's signature   Date (dd/mmm/yyyy)

Investigator's name (Print)

TABLE OF CONTENT

APPROVAL PAGE OF THE CLINICAL RESEARCH PROTOCOL 2

INVESTIGATOR AGREEMENT 3

TABLE OF CONTENT 4

SYNOPSIS 7

List of Abbreviations 9

STUDY DESIGN 10

EVALUATIONS OF THE STUDY 11

SCHEDULE OF ASSESSMENTS 12

1. INTRODUCTION 13

1.1 LITERATURE REVIEW 13

1.1.1. Spinal muscular atrophy (SMA) 13

1.1.2. Therapeutics in Spinal muscular atrophy 13

1.2 STUDY JUSTIFICATION 13

2. STUDY OBJECTIVES 14

2.1. PRIMARY OBJECTIVES 14

2.2. SECONDARY OBJECTIVES 14

3. METHODOLOGY 14

3.1. STUDY DESIGN 14

3.2. STUDY DURATION 14

3.3. EXPERIMENTAL PLAN 14

3.4. REQUIRED SUBJECT NUMBER 14

3.5. RISK-BENEFIT BALANCE 15

4. POPULATION STUDIED 15

4.1. INCLUSION CRITERIA 15

4.2. EXCLUSION CRITERIA 16

4.3. METHOD OF RECRUITMENT 16

5. STUDY ENDPOINTS 16

6. CONDUCT OF THE STUDY FOR ONE PATIENT 17

6.1. VISITS CALENDAR 17

6.1.1. Inclusion Visit 17

6.1.2. Follow up Visit 18

6.2. DESCRIPTIONS OF THE EVALUATIONS 19

6.2.1. Evaluations Order 19

6.2.2. Clinical Evaluation 20

6.2.3. SMA Natural history/Disease Progression Questionnaire 20

6.2.4. Pulmonary Function Tests 20

6.2.5. Muscular testing *22*
Strength Assessments 22
Motor Function Assessment 23
6.2.6. Timed tests *25*
6.2.7. Video recording *25*
6.2.8. Activity monitoring *25*
6.2.9. NMR Imaging (MRI) *25*
6.2.10. Electrophysiology measurement *27*
6.2.11. Quality of Life Assessment *27*
6.2.12. Biological Analysis *28*
Clinical Genotyping: 28
Dynamic Biomarkers: 28
Regulatory aspects in blood sampling: 28
6.3. END OF THE STUDY AND PREMATURE DISCONTINUATION 29
7. DATA MANAGEMENT AND STATISTICAL ANALYSIS 29
7.1. DATA MANAGEMENT 29
7.2. STATISTICAL ANALYSIS 30
8. CHANGES IN THE CONDUCT OF THE STUDY 30
8.1. PROTOCOL AMENDMENTS 30
8.2. PREMATURE STUDY TERMINATION 31
9. ETHICAL CONSIDERATIONS 31
9.1. INDEPENDENT ETHICS COMMITTEE/INSTITUTIONAL REVIEW BOARD 31
9.2. INFORMED CONSENT 31
9.3. DATA PRIVACY 32
9.4. DATA SAFETY MONITORING BOARD 32
9.5. STEERING COMMITTEE 32
10. ASSESSMENT OF SAFETY 33
10.1. DEFINITIONS 33
10.2. MANAGEMENT OF SERIOUS ADVERSE EVENT 33
11. REGULATORY CONSIDERATIONS 34
11.1. REGULATORY CONSIDERATIONS 34
11.2. REGULATORY APPROVAL / AUTHORIZATION 34
11.3. INVESTIGATORS’ OBLIGATIONS 34
11.4. INSURANCE 35
11.5. FINANCIAL DISCLOSER 35
12. QUALITY CONTROL AND QUALITY ASSURANCE 35
12.1. PERIODIC MONITORING 35
12.2. AUDIT AND INSPECTION 35
13. DATA HANDLING AND RECORD KEEPING 36
13.1. SOURCE DATA AND DOCUMENTS 36
13.2. CASE REPORT FORMS 36

14. CLINICAL STUDY REPORT AND PUBLICATION 37
14.1. CLINICAL STUDY REPORT 37
14.2. CONFIDENTIALITY OF STUDY DATA 37
14.3. PUBLICATION POLICY 37
15. ARCHIVING 38
15.1. INVESTIGATOR SITE FILE 38
15.2. TRIAL MASTER FILE 38
BIBLIOGRAPHY 39
APPENDIX 1. SMA QUESTIONNAIRE 41
APPENDIX 2. MUSCLE STRENGTH ASSESSMENT 46
MYOGRIP 46
MYOPINCH 46
APPENDIX 3. MOTOR FUNCTION ASSESSMENT 47
MOVIPLATE 47
MOTOR FUNCTION MEASURE (MFM) 48
ACTIVE-SEATED 56
APPENDIX 4. ACTIVITY ASSESSMENT 57
ACTIMYO 57
APPENDIX 5. MRI EXAMINATIONS SAFETY SHEET 58
APPENDIX 6. QUALITY OF LIFE 59
PEDSQL**Erreur ! Signet non défini.**
PEDIATRIC QUALITY OF LIFE INVENTORY (PedsQL™) 4.0 Generic Core Scale and the PedsQL™ 3.0 Neuromuscular Module 59
HUI2 – MULTI-ATTRIBUTE HEALTH STATUS CLASSIFICATION SYSTEM 60
APPENDIX 7. SERIOUS ADVERSE EVENT FORM 61

SYNOPSIS

**Study Title**	Prospective Study of the Natural History of patients with type 2 and 3 Spinal Muscular Atrophy
**Coordinating Investigator**	Dr Laurent SERVAIS
**Study Centers**	Multicenter study:<br>France: Paris, Toulouse, Lille, Strasbourg, Nantes, Lyon<br>Belgium: Liège, Leuven<br>Germany: Essen
**Study Duration**	36 months
**Duration of Participation for One Patient**	24 months
**Duration of Recruitment Period**	12 months
**Study objectives**	Primary objective:<br>• To characterize the disease course in patients with type 2 and 3 SMA<br>Secondary objective:<br>• To identify prognostic variables of the disease<br>• To identify the best outcome measure for further therapeutics approaches<br>• To identify biomarkers of SMA progression
**Study Design**	Prospective, longitudinal and interventional study
**Number of Subjects**	81
**Experimental Plan**	Because of the variability of phenotypes and ages of patients who may participate to this study, we plan to adjust the assessments to the age and ambulant status of the patients.
**Selection Criteria**	Inclusion criteria:<br>o Type 2 or 3 Spinal Muscular Atrophy, genetically confirmed<br>o Age superior or equal to 2 years old up to 30 years of age included<br>o For patients 6 years and older, willing and able to comply with all protocol requirements and procedures<br>o For non-ambulant patients, able to sit in a wheelchair for at least three hours<br>o Patients over 18 years of age and parent(s)/legal guardian(s) of patients < 18 years of age must provide written informed consent prior to participating in the study and informed assent will be obtained from minors at least 7 years of age when required by regulation.<br>o For France only: Affiliated to or a beneficiary of a social security category<br>Exclusion criteria:<br>o Previously treated with an investigational drug within 6 months prior the recruitment in this study.<br>o Other condition which may significantly interfere with the assessment of the SMA and is clearly not related to the disease<br>o Current or anticipated participation in any therapeutic investigational clinical studies<br>o Patients with specific contraindication to MRI (i.e. metallic foreign body, claustrophobia, and other reasons as determined by investigators) will be allowed to participate, but MRI will not be performed.<br>o For women : pregnancy or current breastfeeding
**Study Endpoints**	Study endpoints will be adjusted according to the age and the ambulant status of the patient. The following will be assessed at baseline and changes over time will be recorded:

	o Respiratory function assessment (from interview, medical files, and standardized measurements)<br>- Respiratory Support (see APPENDIX 1) (Yes/No, type of support, no. of hours on ventilation per day, no. of infections per year, sleep apnea, and forced vital capacity<br>- Pulmonary Function Tests (PFT) (for patients older than 6 Y):<br>• Forced Vital Capacity (FVC)<br>• Peak Cough Flow (PCF)<br>• Maximum Expiratory Pressure (MEP)<br>• Maximum Inspiratory Pressure (MIP) and Sniff Nasal Inspiratory Pressure (SNIP) (both when possible; if not, MIP will be chosen)<br>o Motor function assessment (from interview, medical files, and standardized measurements)<br>- Achieved Milestones: ability to have head control, to sit , to stand up, to walk and others (see APPENDIX 1) (Yes or No, age achieved, age lost)<br>- Standardized assessment (adjusted to the age and the ambulant status):<br>• Evolution of time to rise from floor,<br>• Evolution of time to walk 10 meters,<br>• Evolution of time to climb and descend stairs,<br>• Distance walked on the 6 MWT,<br>• Scores at Moviplate<br>• Total reached volume of upper extremity function by ACTIVE-seated (% predicted value)<br>- Scales:<br>• Motor Function Measure: MFM20 or MFM32 depending on the age of the patient combined with Kinect-MFM (Paris, Liege and Lyon sites only)<br>o Activity assessment: as measured by ActiMyo, only in patients older than 6 Y<br>o Muscle strength assessment (for patients older than 6 Y):<br>- Myogrip<br>- Myopinch<br>o Electrophysiology measurement (EMG):<br>- Compound Motor Action Potential (CMAP) Amplitude<br>- Decrement search<br>o Muscle and spinal cord nuclear magnetic resonance (NMR) imaging (MRI): Muscle volume changes, intramuscular fatty infiltration progression, indices of disease activity only in 2 sites Paris and Strasbourg and only for patients older than 4 Y<br>o Quality of life assessment: PedsQL, HUI2<br>o Clinical Genotyping: Copy number of *SMN2* gene, confirmation of *SMN1* gene mutation, study of the sequence and the genomic region of *SMN* genes and genes influencing the disease severity or progression<br>o Biomarker: SMN mRNA and protein analysis, exploratory biomarkers (e.g. mRNA, DNA profiling, RNA profiling, proteomic profiling)
**Conduct of the study**	A visit for inclusion and 1 follow-up visit every 6 months
**Statistical method**	The study's data analyses are designed primarily to characterize the progression of disease in patients with SMA type 2 and type 3 over a period of 24 months with the goal of exploring suitable outcome measure(s) for future treatment studies. One interim analysis will be performed after 40 patients have completed their 6 months follow up visit and reports thereof will be prepared. A final report will summarize findings after all patients have completed their follow-up. Further details on the statistical methods that will be applied are described in the statistical analysis plan.

List of Abbreviations

CFR	Code of Federal Regulations
CMAP	Compound Motor Action Potential
DMD	Duchenne Muscular Dystrophy
ECG	Electrocardiogram
CRF	Case Report Form
EMG	Electrophysiology Measurement / Electromyography
FDA	United States Food and Drug Administration
FVC	Forced Vital Capacity
GCP	Good Clinical Practice
HUI2	Health Utilities Index Mark 2
ICF	Informed Consent Form
ICH	International Conference on Harmonization
IEC	Independent Ethics Committee
IRB	Institutional Review Board
LVEF	Left Ventricular Ejection Fraction
MEMS	Microelectromechanical Systems
MEP	Maximum Expiratory Pressure
MFM	Motor Function Measure
MIP	Maximum Inspiratory Pressure
MRI	Magnetic Resonance Imaging
MVIC	Maximal Voluntary Isometric Contractions
NCI CTCAE	National Cancer Institute Common Terminology Criteria for Adverse Events
NMR	Nuclear Magnetic Resonance
PCF	Peak Cough Flow
PFT	Pulmonary Function Tests
s	Second(s)
SMA	Spinal Muscular Atrophy
SMN1	Survival of motor neuron 1, telomeric gene
SMN2	Survival of motor neuron 2, centromeric gene
SMN	Survival motor neuron protein
SNIP	Sniff Nasal Inspiratory Pressure
6MWT	Six-Minute Walk Test
Y	Year(s)

STUDY DESIGN

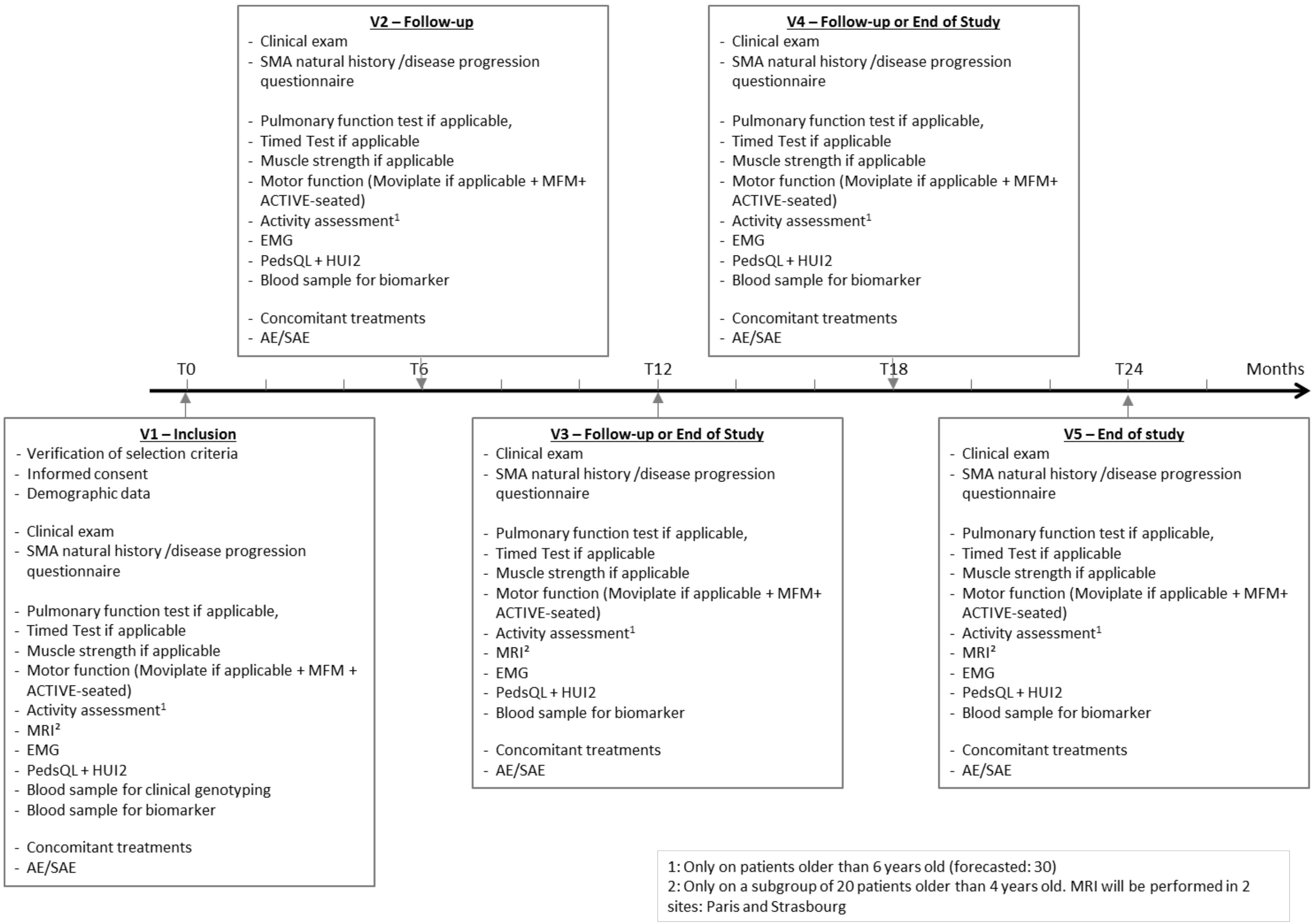

Figure 1 : Study design

EVALUATIONS OF THE STUDY

We plan to adjust the evaluations of the study according to the age and the ambulant status[1] of the patient as shown in the table below. All evaluations will be performed at each visit.

	**2 – 5 years old**	**6 – 30 years old**
**Ambulant**	- **Physical exam**<br>- **SMA Natural history/disease progression questionnaire:**<br>Achieved Milestone: Acquisition/loss (Yes/No/Never acquired + Age)<br>Respiratory function: Type of respiratory support (mechanical ventilation or noninvasive ventilation), number of hours off ventilation/day, respiratory lower tract infection /year, sleep apnoea (sleep disorder due to sleep apnoea)<br>Orthopedic status: Scoliosis, contractures, fracture, assistive device<br>Feeding status: Difficulty for feeding, need of a feeding tube<br>- **Motor function**:<br>Scale: MFM20[5]<br>- **Electrophysiology measurement (EMG):** CMAP, decrement search<br>- **NMR[2]**: Muscle volume, Fatty infiltration, indices of disease activity<br>- **Quality of Life**: Number of work days missing due to caring for a child with SMA, number of school missing due to SMA, number of family or social event missing due to SMA, PedsQL, HUI2[3]<br>- **Blood sample**: *SMN* mRNA quantification, SMN protein levels, SMA Exploratory biomarkers, Genotyping (at baseline only)	- **Physical exam**<br>- **SMA Natural history/disease progression questionnaire:**<br>Achieved Milestone: Acquisition/loss (Y/N/Never acquired + Age)<br>Respiratory function: Type of respiratory support (mechanical ventilation or noninvasive ventilation), number of hours off ventilation/day, Respiratory lower tract infection /year, Sleep apnoea (sleep disorder due to sleep apnoea)<br>Orthopedic status: Scoliosis, contractures, fracture, assistive device<br>Feeding status: difficulty for feeding, need of a feeding tube<br>- **Motor function**:<br>Standardized tests Timed tests: time to get up from floor, to walk 10 meters, to climb and descend stairs, Distance walked in 6MWT, Moviplate and ACTIVE-seated<br>Scale: MFM32[5]<br>- **Electrophysiology measurement (EMG):** CMAP, decrement search<br>- **NMR[2]**: Muscle volume, Fatty infiltration, indices of disease activity<br>- **Quality of Life**: Number of work days missing due to caring for a child with SMA, number of school missing due to SMA, number of family or social event missing due to SMA, PedsQL, HUI2[3]<br>**Blood sample**: *SMN* mRNA quantification, SMN protein levels, SMA Exploratory biomarkers, Genotyping (at baseline only)<br>- **Pulmonary Function tests :** FVC, PCF, MEP, MIP, SNIP<br>- **Muscle strength assessment**: Myogrip, Myopinch<br>- **Activity assessment[4]**: ActiMyo
**Non-Ambulant**	- **Physical exam**<br>- **SMA Natural history/disease progression questionnaire:**<br>Achieved Milestone: Acquisition/loss (Y/N/Never acquired + Age)<br>Respiratory function: Type of respiratory support (mechanical ventilation or noninvasive ventilation), number of hours off ventilation/day, Respiratory lower tract infection /year, Sleep apnoea (sleep disorder due to sleep apnoea)<br>Orthopedic status: Scoliosis, contractures, fracture, assistive device<br>Feeding status: difficulty for feeding, need of a feeding tube<br>- **Motor function**:<br>Scale: MFM20[5]<br>- **Electrophysiology measurement (EMG):** CMAP, decrement search<br>- **NMR[2]**: Muscle volume, Fatty infiltration, indices of disease activity<br>- **Quality of Life**: Number of work days missing due to caring for a child with SMA, number of school missing due to SMA, number of family or social event missing due to SMA, PedsQL, HUI2[3]<br>**Blood sample**: *SMN* mRNA quantification, SMN protein levels, SMA Exploratory biomarkers, Genotyping (at baseline only)	- **Physical exam**<br>- **SMA Natural history/disease progression questionnaire:**<br>Achieved Milestone: Acquisition/loss (Y/N/Never acquired + Age)<br>Respiratory function: Type of respiratory support (mechanical ventilation or noninvasive ventilation), number of hours off ventilation/day, Respiratory lower tract infection /year, Sleep apnoea (sleep disorder due to sleep apnoea)<br>Orthopedic status: Scoliosis, contractures, fracture, assistive device<br>Feeding status: difficulty for feeding, need of a feeding tube<br>- **Motor function**:<br>Standardized tests: Moviplate and ACTIVE-seated<br>Scale: MFM32[5]<br>- **Electrophysiology measurement (EMG):** CMAP, decrement search<br>- **NMR[2]**: Muscle volume, Fatty infiltration, indices of disease activity<br>- **Quality of Life**: Number of work days missing due to caring for a child with SMA, number of school missing due to SMA, number of family or social event missing due to SMA, PedsQL, HUI2[3]<br>**Blood sample**: *SMN* mRNA quantification, SMN protein levels, SMA Exploratory biomarkers, Genotyping (at baseline only)<br>- **Pulmonary Function tests:** FVC, PCF, MEP, MIP, SNIP<br>- **Muscle strength assessment**: Myogrip, Myopinch<br>- **Activity assessment[4]**: ActiMyo

1: A patient is defined as ambulant if able to walk 10 meters without human or technical help

2: For a subgroup of 20 patients older than 4 years old, only in 2 sites Paris and Strasbourg

3: Fertility attribute being optional and not part of the standard HUI23-15Q questionnaires, this question will be remove from the forms

4: For a subgroup of 30 patients older than 6 years old

5: Combined with Kinect-MFM only in Paris, Liège and Lyon sites

SCHEDULE OF ASSESSMENTS

	2 – 5 years old		≥ 6 – 30 years old	
	Inclusion	Follow up visit	Inclusion	Follow up visit
	M0	Every 6 months ± 28 days	M0	Every 6 months ± 28 days
**Verification of selection criteria**	X		X	
**Informed consent**	X		X	
**Demographic data**	X		X	
**Physical examination and vital signs**[1]	X	X	X	X
**SMA natural history/disease progression questionnaire:**<br>- Achieved Milestone<br>- Respiratory function<br>- Number of school and/or work days and family or social event missing due to SMA<br>- Other assessments from the medical file (feeding status, orthopedic status)	X	X	X	X
**Pulmonary function Tests:**<br>- FVC<br>- PCF<br>- MEP<br>- MIP and/or SNIP[2]			X	X
**Timed Test**[3]**:**<br>- Time to rise from floor<br>- Time to walk/run 10 meters<br>- Time to climb and descend stairs<br>- Distance walked in 6MWT			X	X
**Moviplate**			X	X
**Muscle strength:**<br>- Myogrip<br>- Myopinch			X	X
**Activity assessment**[4]			X	X
**MFM**[7]	MFM20[5]	MFM20[5]	MFM32	MFM32
**ACTIVE-seated**			X	X
**PedsQL**	X	X	X	X
**HUI2**	X	X	X	X
**MRI**[6]	X	X	X	X
**EMG**<br>- CMAP<br>- Decrement search	X	X	X	X
**Blood Sample for clinical genotyping**	X		X	
**Blood Sample for biomarker**	X	X	X	X
**Concomitant Treatments**	X	X	X	X
**AE/SAE**		X		X

[1] Weight, height, body temperature, heart rate, respiratory rate, systolic and diastolic blood pressure
[2] MIP and SNIP when possible. If not possible to perform both because of fatigue, MIP will be chosen
[3] Only for ambulant patients defined as patient being able to walk 10 meters without human or technical help
[4] Only on a subgroup of 30 patients
[5] MFM32 can be chosen for patients older than 4Y, depending on patient's abilities
[6] Only for 20 patients older than 4Y; only in 2 sites in Paris and Strasbourg – every 12 months
[7] Combined with Kinect-MFM only in Paris, Liège and Lyon sites

Table 1 : Study schedule description

1. INTRODUCTION

1.1 LITERATURE REVIEW

1.1.1. Spinal muscular atrophy (SMA)

Spinal muscular atrophy (SMA) is the second most frequent autosomal recessive disorder worldwide. Its incidence is approximately 1:10 000 (EMERY 1991; MERLINI *et al.* 1992; ARKBLAD *et al.* 2009). It is caused by homozygous absence of the *SMN1* gene on chromosome 5q13 (MELKI *et al.* 1990; LEFEBVRE *et al.* 1995) and results in degeneration of the spinal cord motor neurons. Clinical manifestations include muscle atrophy and varying degrees of weakness. SMA classification is based on clinical findings, time of onset, and maximal achieved motor milestone (MUNSAT AND DAVIES 1992; MERCURI *et al.* 2012).

The natural history of patients with SMA has changed in the past decade due to aggressive intervention that has improved survival and quality of life (SCHROTH 2009). In the severe form characterized by an onset before 6 months, SMA type I, children have impaired head control, never acquire a sitting position, and usually die before the age of two years. SMA type II, is of intermediate severity with an onset between 7 and 18 months of age. Children with SMA type 2 achieve the ability to sit independently but never stand or walk independently. Those with SMA type 3 achieve the ability to stand and walk independently, but about half of the patients lose this ability before the age of 18. (RUSSMAN *et al.* 1996; RUDNIK-SCHONEBORN *et al.* 2001). The SMA phenotype varies within each SMA type, covering a wide range of functional abilities. Co-morbidities of patients with SMA type 2 and 3 may include orthopedic, respiratory or nutrition problems (HAAKER AND FUJAK 2013).

1.1.2. Therapeutics in Spinal muscular atrophy

Presently, there is no effective therapy to treat the muscle weakness in SMA patients. Current therapeutic strategies include mainly respiratory, feeding and orthopedic management and supportive pharmacologic therapy. These therapies improve muscular function, quality of life and longevity, but do not directly target the disease mechanism. The use of salbutamol, for which some positive effects have been suggested, varies widely, from country to country and center to center.

With the progress made in the knowledge of genetics, molecular biology, vectorology and muscle physiology of myopathies, several promising experimental strategies, including gene therapy (BUJ-BELLO *et al.* 2008), are currently under investigation.

Recently, Trophos Company reported positive results in a Phase III trial for olesoxime. Patients with SMA type 2 and 3 presented a significant slower decline in MFM function and a trend for a slower decline in secondary outcome measures. However, given the need of a complementary study, olesoxime will not likely be on the market before 2016.

1.2 STUDY JUSTIFICATION

Several therapeutic strategies for treatment of SMA are under investigation. However, no effective treatment has currently received market approval. Taking into account the variability of the phenotype of SMA patients, even for very young ones, it will be useful to determine which outcome measure(s) will be the most appropriate to assess the efficacy of potential therapies and which variables are prognostic of the course of the disease.

The proposed study is a prospective study of the SMA natural history, the purpose of which is to characterize the disease course over 2 years and determine which outcome measures will be the best to assess the efficacy of potential therapies.

2. STUDY OBJECTIVES

2.1. PRIMARY OBJECTIVES

Primary objective of this study is to characterize the disease course in SMA type 2 and type 3 patients using standardized evaluations.

2.2. SECONDARY OBJECTIVES

Secondary objectives are

- To identify prognostic variables of the disease
- To identify the best outcome measure(s) for future treatment studies
- To identify biomarkers of SMA progression

3. METHODOLOGY

3.1. STUDY DESIGN

The present study is a prospective, longitudinal and interventional study of pathophysiology.

3.2. STUDY DURATION

The study duration is 3 years (36 months) with a period of recruitment of 12 months.

Therefore, the duration for participation for each patient will be 24 months.

3.3. EXPERIMENTAL PLAN

Taking into account the variability of phenotypes and the variability of the age of patients who may participate to this study, we plan to adjust the assessments to the age and the ambulant status of the patient. All patients will be evaluated every 6 months.

3.4. REQUIRED SUBJECT NUMBER

Since SMA is a rare disease, the participation to the trial will be proposed to all patients affected by SMA type 2 and type 3 and who fulfill the inclusion criteria in 6 centers in France (Paris, Toulouse, Lille, Strasbourg, Nantes and Lyon), 2 centers in Belgium (Liège and Leuven) and 1 center in Germany (Essen).

A recent one year natural history study in non-ambulant patients has demonstrated that significant loss strength can be demonstrated after 14 years in a limited number of patients (n=7) (A.M. Seferian Submitted). However, given the heterogeneity of the disease and the large age range planned in the inclusion criteria, we want to gather at least one year follow up data in 50 eligible patients. From our previous experience in natural history studies, we anticipate more than 25% drop out of the study; we thus want to include 81 patients.

3.5. RISK-BENEFIT BALANCE

There is no expected benefit for patients who participate in this trial, except a better knowledge of their pathology.

There is no risk to participate to this study. The majority of assessments are non-invasive (except blood sampling) and do not expose patients to a serious risk. Blood sampling pose the patient to a minimal risk; common side effects are mild pain at the site of venipuncture, very small hemorrhages, coloration of the skin and mild discomfort. Venipuncture will not be attempted more than two or three times by the same person; it will be passed to a more experienced practitioner in this case. There is a minimum risk of venipuncture site infection associated with blood withdrawal when proper skin hygienic measures are taken. The risk of cross infection will be managed by standard infection control precautions in particular by: A) Needles management: Where available a safety device should be used to prevent needlestick injury; needles must be disposed of immediately after use in a sharps container; B) Hand hygiene: hand washing and disinfection must be performed prior and after to undertaking the procedure.

Muscular testing and timed tests will be stopped if patients experience fatigue, pain or cramps and will be started again after a sufficient period of rest. Patient could come in two consequent days to have their assessments done, so that it will be easier for them to cope with all the assessments.

Regarding the risks related to the radiofrequency energy absorbed by the patient, the protocol will comply with the IEC-601-2-33 standard which defines the maximum acceptable dose received by a patient during NMR studies. The Specific Absorption rate (SAR) will not exceed level 1 (not more than 4 W/kg).

4. POPULATION STUDIED

4.1. INCLUSION CRITERIA

- Type 2 or 3 spinal muscular atrophy genetically confirmed
- Age superior or equal to 2 years old up to 30 years of age included
- For patients older than 6 years old, willing and able to comply with all protocol requirements and procedures.
- For non-ambulant patients, able to sit upright in a wheelchair for at least three hours
- Patients over 18 years of age and parent(s)/legal guardian(s) of patients < 18 years of age must provide written informed consent prior to participating in the study and informed assent will be obtained from minors at least 7 years of age when required by regulation.
- In France only: Affiliated to or a beneficiary of a social security category

4.2. EXCLUSION CRITERIA

- Previously treated with an investigational drug within 6 months prior the recruitment in this study.
- Other condition which may significantly interfere with the assessment of the SMA and is clearly not related to the disease
- Current or anticipated participation in any therapeutic investigational clinical studies.
- Patients with specific contraindication to MRI (i.e. metallic foreign body, claustrophobia, and others deemed to be prohibitive by the investigators) will be allowed to participate, but MRI will not be performed [cf. APPENDIX 5, page 58].
- For women : pregnancy or current breastfeeding

According to the law, patients will be allowed to withdraw their consents at any time of the study.

4.3. METHOD OF RECRUITMENT

This study will be open to all eligible patients. Patients will be identified *via* several means:

- Patients will be selected from active file of investigator sites.
- The study will be presented to the French network of child neurologists.
- The study will be listed on www.clinicaltrial.gov and presented to applicable advocacy groups.
- Self-informed patients, who meet the inclusion criteria, will also be allowed to take part into the study.

5. STUDY ENDPOINTS

The study endpoints will be defined by the age and ambulant status of the patient. An ambulant patient is defined as a patient able to walk 10 meters without human or technical help The following will be assessed at baseline and changes over time will be recorded:

- Respiratory function assessment (from interview, medical file and pulmonary function tests):
  - Type of respiratory support (Invasive/Noninvasive)
  - Number of hours off mechanical ventilation per day
  - Number of lower respiratory tract infection per year
  - Pulmonary function tests (PFT) for patients older than 6Y and not completely dependent on mechanical ventilation: forced vital capacity (FVC), peak cough flow (PCF), maximum expiratory pressure (MEP), maximum inspiratory pressure (MIP) and/or sniff nasal inspiratory pressure (SNIP)
- Motor function assessment (from interview, medical file and standardized measurements):
  - Achieved Milestone: ability to have head control (Yes/No/Never acquired, age achieved, age lost), to sit, to stand up, to walk with or without help, to climb stairs, to feed oneself and others [see APPENDIX 1, page 41]
  - Standardized assessment (adjusted to age and ambulant status): Evolution of time to get up from floor, to walk 10 meters, to climb and descend stairs, the evolution of the distance

walked at the 6 MWT, the measured values for Moviplate, and of the % predicted of total reached volume of upper extremity function by ACTIVE-seated

- Scales: Motor Function Measure (MFM) 20 or 32 depending on the age and combined with Kinect-MFM only in Paris, Liege and Lyon sites.

- Activity assessment: Evolution of activity as measured by ActiMyo (in patients older than 6 years old, forcasted: 30 patients)
- Muscle strength assessment: Evolution of measured values for Myogrip and Myopinch
- Electrophysiology measurement (EMG)
  - Evolution of the Compound Motor action Potential Amplitude (CMAP)
  - Decrement search
- Muscle nuclear magnetic resonance imaging (MRI) (on a subgroup of 20 patients older than 4 years only in 2 sites Paris and Strasbourg):
  - Muscle volume changes
  - Intramuscular fatty infiltration progression
  - Indices of disease activity
- Quality of life assessment (from questionnaire and interview):
  - Number of work days missing due to caring for a child with SMA, number of days of school or work missing due to the SMA
  - Family and community events missed due to illness or care
  - PedsQL
  - HUI2
- Orthopedic status (from medical file): Scoliosis, contractures, fractures, use of assistive device
- Biomarker: *SMN* mRNA quantification, SMN protein production, exploratory biomarkers, genotyping (mRNA, DNA and protein profiling)

6. CONDUCT OF THE STUDY FOR ONE PATIENT

6.1. VISITS CALENDAR

6.1.1. Inclusion Visit

During this visit, investigator will verify selection criteria, will answer all the questions regarding the present study and will propose to the patient/parents or legal guardian of the patient to sign the informed consent.

Upon signature of the informed consent, the screened patients will be considered as included and able to undergo baseline evaluations listed below. The time between brackets is an approximation:

- **For all patients:**
  - Clinical evaluation (60 min)
  - SMA natural history/disease progression questionnaire (15 min)

- MRI (120 min) (On a subgroup of 20 patients older than 4 years old; only in 2 sites Paris and Strasbourg)
- EMG (30 min)
- Quality of Life: PedsQL, HUI2 (30 min)
- List of concomitant treatments (5 min)
- Blood sampling (10 min)

o **According to the age:**

Between 2 and 6 years old:

Motor Function Assessment: MFM20 (45 min) (MFM32 can be chosen for patients older than 4 years old, depending on the patient's abilities – the MFM scale initially chosen, 20 or 32, cannot be changed thereafter)Above 6 years old:

- Pulmonary function tests for patients not receiving continuous mechanical ventilation (45 min)
- Muscular strength measurement: Myogrip, Myopinch (30 min)
- Motor function assessment: ACTIVE-seated (20 min), Moviplate, MFM32; and for ambulant patient: timed tests (90 min)
- In a subgroup of 30 patients, delivery of the Actimyo, explanation of its functioning (30 min)

All data will be recorded in the case report form (CRF) as soon as possible after the visit.

6.1.2. Follow up Visit

During this visit, the following evaluations will be performed according to the age of the patient:

o **For all patients:**

- Clinical evaluation (60 min)
- Checking the occurrence of adverse events or serious adverse events (5 min)
- List of concomitant treatments (5 min)
- Questionnaire of SMA follow-up (15 min)
- MRI once a year (120 min) (On a subgroup of 20 patients older than 4 years old; only in 2 sites Paris and Strasbourg)
- EMG (30 min)
- Quality of Life: PedsQL, HUI2 (30 min)
- Blood sampling

o **According to the age:**

Between 2 and 6 years old:

- Motor Function Assessment: MFM20 (45 min) (or MFM 32 for patients older than 4 years old, if chosen at the inclusion visit) combined with Kinect-MFM only in Paris, Liege and Lyon sites.

Above 6 years old:

- Pulmonary function tests for patients not receiving continuous mechanical ventilation (45 min)
- Muscular strength measurement: Myogrip, Myopinch (30 min)
- Motor function assessment: ACTIVE-seated (20 min), Moviplate, MFM32 combined with Kinect-MFM only in Paris, Liege and Lyon sites; and for ambulant patients: timed tests (90 min)
- Checking the Actimyo (15 min) in the subgroup of 30 patients,

All data will be recorded in the case report form (CRF) as soon as possible after the visit.

6.2. DESCRIPTIONS OF THE EVALUATIONS

6.2.1. Evaluations Order

Given the numerous evaluations to be performed, the patient study visits are extensive. Accordingly, these visits may be conducted either as one-day visit or over two days, whichever is preferred by the patient and possible for the clinical site. Four blocks of evaluations and recommended evaluations order within each block have been defined (Table 1). Block of evaluations should always be conducted in this order. Within each block, the assessments can however be performed in any order. Breaks are recommended at any time as appropriate for each patient. It is also recommended that for a single patient, assessments are conducted in the same order throughout the trial.

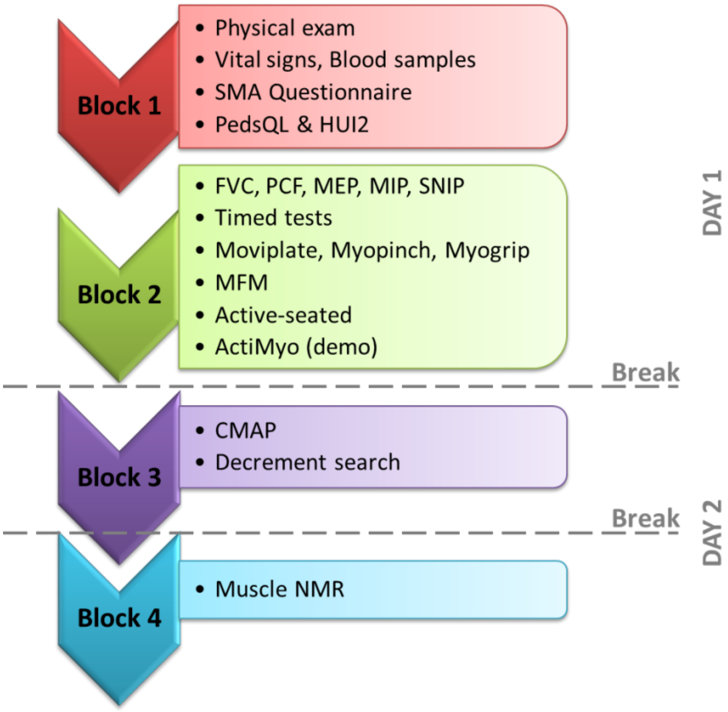

Table 1:

Defined blocks of assessments order and recommended evaluations order within each block at each visit

6.2.2. Clinical Evaluation

The clinical evaluation performed at each visit will contain:

- A complete medical history including perinatal period, family history, previous and concomitant diseases, previous hospitalization in relation with SMA and concomitant treatments
- A complete physical examination will include the following organs and systems: hair and skin; lymph nodes; eyes; ear, nose, throat; respiratory; cardiovascular; abdomen; musculoskeletal; neurological; mental status; ambulant status; brooke score
- Measurement of the patient's weight and height
- Vital signs measurements: body temperature, heart rate, respiratory rate, systolic and diastolic blood pressure. Each measure will be performed one time. The blood pressure will be measured in a seated position.

Major events notable for the disease progression that do not constitute an AE should also be noted on physical examination.

6.2.3. SMA Natural history/Disease Progression Questionnaire

The aim of this questionnaire is to collect data from the usual follow-up of these SMA patients including:

- Quality of Life: Number of work days missing due to caring for a child with SMA, Number of school or work days missed due to SMA, number of family or social events missed due to SMA
- Psychomotor development (including cognitive assessment if performed)
- Respiratory function assessment: Type of breathing support (mechanical ventilation or non-invasive ventilation), the number of hours of ventilation per day and number of respiratory lower tract infections per year, sleep apnoea and vital capacity if known
- Feeding status: feeding difficulties, need of a feeding tube
- Orthopedic status: scoliosis, contracture, fractures, use of assistive device

[APPENDIX 1, page 41]

6.2.4. Pulmonary Function Tests

Assessment of the respiratory function will be performed by a certified physiotherapist as indicated in the schedule of assessment. The measured parameters will be forced vital capacity (FVC), peak cough flow (PCF), maximum expiratory and inspiratory pressures (MEP and MIP) or sniff nasal inspiratory pressure (SNIP).

To prevent air leaks, nose will be blocked by a nose clip for every respiratory assessment.

For each parameter, attempt should be repeated at least three times making sure that the subject has recovered between attempts. If at each attempt there is an improvement in the results further tests can be conducted until the subject has achieved his best result. The best result of the different attempts will be used for statistical analysis.

- Forced Vital Capacity (FVC): it is the maximum amount of air that a person can exhale as hard, fast and as long as possible from the lungs after a maximum inspiration effort. It is equal to the inspiratory reserve volume plus the tidal volume plus the expiratory reserve volume. The vital capacity will be

evaluated by measuring the volume of air mobilized during breathing, the patient breathing normally through the mouth into a rubber mouthpiece connected to a spirometer and electronic measuring device. After breathing out normally to full expiration, at a given signal, the patient will be instructed and encourage to inspire as deep as possible with a maximal effort, and then blow (out) as forcefully, rapidly and long as possible (for at least 6 s in adults, 3 s for children).

- Peak Cough Flow (PCF): This examination assesses the ability of the person to remove bronchial secretions by coughing. It tests the ability to exhale a volume of air through the expiratory muscle strength: this is the peak expiratory flow during coughing. The peak cough flow is measured by having the patient inspire fully and then cough forcibly, as hard as possible in a mask or mouthpiece connected to the spirometer or peak flow meter.

- Maximum expiratory and inspiratory pressures (MEP and MIP): These measures assess the overall respiratory muscle strength. The maximal inspiratory pressure (MIP) reflects the strength of inspiratory muscles, while the expiratory pressure reflects the strength of the abdominal muscles and other expiratory muscles. They assess the pressure on a membrane connected to a manometer during forced inspiration or expiration. This examination can detect early respiratory muscle weakness, before the onset of a decrease in vital capacity. Because sniff is a natural effort that many patients find easier to perform than static efforts, the sniff nasal inspiratory pressure (SNIP) was recently proposed as an alternative to the MIP (PRIGENT *et al.* 2004). MIP and SNIP will be performed when possible. If not possible to perform both because of the patient's fatigue, MIP will be chosen.

    - The device used to measure the maximum expiratory pressure (MEP) includes a mouthpiece and is connected to a pressure recorder. The patient's nose is pinched and kept closed. The person is instructed to fully inhale, while holding the rubber mouthpiece firmly in the mouth, and then to blow out (exhale) as hard as possible in one go, similar to inflating a very stiff balloon. The patient should maintain expiratory pressure for at least 1.5 seconds and the largest positive pressure sustained for at least 1 second (not a transient spike) should be recorded. The patient should be allowed to rest for about one minute and then repeat the maneuver five times. Verbal and/or visual feedback will be provided after each maneuver. The goal is for the variability among measurements to be less than 10 cm H2O. Measurements will be rounded to the nearest 5 cm H2O.

    - The device used to measure the maximum inspiratory pressure (MIP) is the same as for measuring the MEP. The patient's nose is pinched and kept closed. The patient will be instructed to exhale slowly and completely and then to inhale as strong and hard as possible through the mouthpiece (as if he is trying to suck up a thick milkshake or draw aspired air). The patient should maintain inspiratory pressure for at least 1.5 seconds and the largest negative pressure sustained for at least 1 second (not a transient spike) should be recorded. It is the pressure of this inspiration that is to be measured. The durations are estimated by the individual supervising the test. Patient will be allowed to rest for about one minute and then repeat the maneuver at least three times. Verbal or visual feedback will be provided after each maneuver. The goal is for the variability among measurements to be less than 10 cm H2O. Measurements will be rounded to the nearest 5 cm H2O.

    - Sniff nasal inspiratory pressure (SNIP): After demonstration to the patient, a nasal probe is inserted into his right nostril (or left if not possible with the right one). Starting from the end-expiratory volume after a quiet breath, the patient performs short sharp sniffs (LOFASO *et al.* 2006) or inhale with as much effort as possible (Michelle Eagle DMD114349) through the probe with closed mouth and left (or right) nostril not occluded.

FVC and PCF will be measured by Vitalograph spirometer and software. MEP and MIP (and/or SNIP) will be determined using the MicroRPM device. Both Vitalograph and MicroRPM systems have been used in respiratory assessments in previous clinical trials with DMD patients. Values will be expressed in percent of predicted value.

6.2.5. Muscular testing

All muscular testing will be done by a physiotherapist trained by the Institute of Myology, Paris.

The measurement sessions will take place in a quiet and temperate room. All tests will be performed with the patients sitting on a regular chair or in their wheelchair facing a table, preferably adjustable in height with the forearm placed on the table or on the wheelchair shelf. Before each test, patients will be given a description of the task, a demonstration of the movement required and advice on the correct practice. If necessary, the correct body position will be manually guided. If standard upper limb position described by the protocol cannot be maintained because of the patient's contractures, alternative position will be allowed. The patient will be proposed to be filmed during his evaluations. If the patient agrees, this could be limited on patient's request to a tight frame focused on the hand or to filming specified sequences.

The patients will perform first the strength tests (MyoGrip and MyoPinch, see below) and then the functional tests (MoviPlate, MFM, and ACTIVE-seated, see below). Each patient will be tested for both right and left sides, except for MFM, for which one side only will be tested and ACTIVE-seated which is a different technology. All tests will start with the dominant hand. In this study, we define dominant hand as the one preferred for daily fine activities like writing. Each strength test must be repeated at least three times. If the last try is the stronger, it will be asked to try again until the last try is below than the previous test. The value retained will be the best valid trial. Each test will be repeated consecutively in side alternating at each attempt.

The patients will pass to the next device/evaluation after completing all measurements for both left and right sides. Patients will be given a three-minute rest (or more if required by patient) between each device/evaluation.

For strength assessment and MoviPlate, the test will be carried out with verbal encouragement.

The signals generated using MyoGrip, MyoPinch and MoviPlate will be recorded by dedicated software for quality control.

Strength Assessments

The patient is asked to provide maximal voluntary isometric contractions (MVIC) for about 3 s.

MyoGrip test: It's a precise dynamometric measurement of isometric grip strength [APPENDIX 2, page 46]. The maximal grip strength is directly displayed on the digital display of the tool. It measures force in Newton up to 90 kg with a resolution of 0.01 kg. The handle width will be adapted to the hand size of the patient. The patient will be evaluated in a sitting position and facing a plinth, the knee and hip will be forming a right angle, the trunk will be in an upright position.
The MyoGrip is placed on the table in order for the patient to be positioned as close as possible to the following position:

- elbow bent,
- forearm in neutral pro-supination on the table,

- wrist in slight extension,
- fingers in flexion

In case of contractures preventing comfortable standard position, positioning can be adapted accordingly.

Shoulder of the tested limb will be in neutral rotation and slight flexion and the elbow of the tested hand will be lying on the plinth. The other hand will rest as much as possible on the contralateral thigh.

Patient has to squeeze as strong as possible the MyoGrip handle.

MyoPinch test: The MyoPinch dynamometer [APPENDIX 2, page 46] measure key or tip pinch using a high precision load cell (nominal scale: 15 kg; resolution: 0.001 kg). The load cell is equipped with two steel blades 2 mm afar and presenting an overall thickness of 7 mm. The patient must perform a key pinch on the two blades. The transducer signal is processed by an electronic board, which displays the maximal strength of the patients.
The patient should be positioned as close as possible to the following position:

- elbow bent,
- forearm in neutral pro-supination on the table,
- wrist in slight extension,
- fingers slightly flexed

In case of contractures preventing comfortable standard position, positioning can be adapted accordingly.
Patient has to pinch the thin MyoPinch extremity as hard as possible between the thumb and the second phalanx of the index.
Patients will be evaluated in a sitting position. The evaluator will hold the device to allow the patient to pinch it.

Motor Function Assessment

MoviPlate: The MoviPlate is a device that was designed to measure the ability to produce repeated hand movements between two cylindrical targets aligned in the sagittal plane.(SERVAIS *et al.* 2013) [APPENDIX 3, page 47]. The device is made of a platform with one lower proximal target and one upper distal target (2-cm-higher than the lower target). The MoviPlate is composed of this platform connected with a support adjustable in length for the patient to place his forearm. The patient is seated and asked to press alternately the two targets as many times as possible during 30 seconds. Load cells are placed under both targets. Their detection threshold can be adjusted to the patients' strength by setting up directly on the MoviPlate platform. Only back-and-forth taps are counted and displayed by the device.

The Motor Function Measure (MFM): The MFM is a scale to enable an objective assessment of the motor abilities of patients with neuromuscular diseases whatever the motor deficiency. Three dimensions have been identified with factorial analysis in the validation study: D1 standing position and transfers, D2 axial and proximal motor function and D3 distal motor function. It can be used for ambulant and non-ambulant patient. A short version (20 items versus 32) has been adapted for children between 2 and 6 years old. (BERARD *et al.* 2005; DE LATTRE *et al.* 2013). MFM32 can be chosen at inclusion visit for patients older than 4 years old, depending on the patient's abilities – the MFM scale initially chosen, 20 or 32, cannot be changed thereafter [APPENDIX 3, page 48]

Kinect-MFM (Paris, Liege and Lyon sites only)

The KiMe2 (Kinect Medical Measurement) software is being developed by the GSCOP laboratory and the "Service de médecine physique et de reeducation pédiatrique l'Escale" (Dr Carole Vuillerot's site, Hospices

civils de Lyon, France). It analyzes patient's posture and movements during the MFM assessment. A Kinect will be positioned in front of the patient, and will record the following items: 9, 10, 11, 12, 13, 14, 15, 16, 24, 25, 26, 27, 31, and 32. The MFM assessment should be performed as usual by the therapist. The software calculated score will be compared to the therapist assessed score.

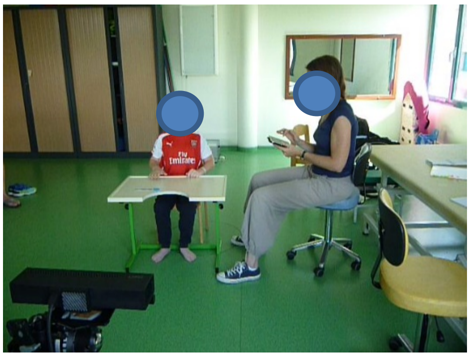

ACTIVE-seated (Ability Captured Through Interactive Video Evaluation-seated) utilizes the Microsoft Kinect controller-free gaming device interface for Windows to measure upper extremity (UE) functional reach volume, while motivating the subject to perform to his maximum ability. The seated skeletal tracking option tracks the head, trunk and arms and identifies one point on the head and sternum, one point on each shoulder and 3 on each arm, thereby enabling measurement of the maximal UE excursion in the X, Y and Z planes to quantify the subject's functional reaching volume (FRV). FRV relates to independence in self-care and other daily activities that directly impact quality of life as these activities are dependent on the function of the trunk, shoulder and elbow muscles to support and maximize reaching abilities.

Testing procedure: Patients will be positioned sitting upright with both hands on a height-adjustable table in front of a screen and Kinect device. The evaluator will provide verbal directions and demonstrate to reach as far as possible in all directions with the UE. Once the trial is started the patients will be encouraged to move his arms as far as possible in 4 directions (left and right, vertically of the table or overhead, and forward toward the camera). A series of boxes will appear on the screen as a visual representation of reach to enhance motivation. If the patient is able to perform antigravity movement with the biceps to get his/her hands off the table, a second box (with a different color than box before) will appear that encompasses the area up to shoulder height. If the patient reaches above the shoulder threshold, a final box (with a different color than the last two) will appear to quantify overhead reaching area. The evaluators will have to encourage patients to safely lean as far as possible to each side and forward to obtain maximal scores on ACTIVE-seated. Compensatory movements are allowed when playing ACTIVE-seated as these are representative of movements used in daily life to maintain independence with functional activities.

Each trial last up to 60 s and will allow patients to complete several trials to obtain their best performance while minimizing fatigue. Subjects will complete a series of 3 trials and on each subsequent trial the previous performance will be highlighted on the screen to encourage reaching farther to attain a higher "reach score".

At the end of each trial, the volume of each box will be automatically recorded in cubic meters for analysis and summed to create the total FRV value that will be reported in cubic meters. Raw FRV will be converted into percent predicted FRV (ppFRV) based on estimated height of patient (Ulnar length will be used) to compensate development parameters as size and growth of UE and/or trunk. Calculated ppFRV is a scaled score that can be compared over time within and across subjects.

6.2.6. Timed tests

Six-Minute Walk Test (6MWT): The 6MWT is an objective evaluation of functional exercise capacity which measures how far a person can walk in six minutes. It is a global measure of multiple body systems including cardiopulmonary, vascular and neuromuscular. 6MWT will be performed as described by McDonald in 2010 (McDonald *et al.* 2010).

10 meter timed walk/run test: The test provides a quantitative measure of how fast a patient can travel a distance of 10 m.

Time to rise from floor test: The test provides a quantitative measure of how fast the patient gets up from the floor from a supine position.

Time for climbing stairs test: The test provides a quantitative measure of how fast the patient can climb and descend 4 standardized stairs.

6.2.7. Video recording

The functional test such as MoviPlate as well as timed test could be video-recorded after signed consent to ensure quality of data collection and to constitute a visual record of subject ability. If the patient or his representative refuses the video recording, this does not constitute an exclusion criterion, and the video recording will not be realized.

6.2.8. Activity monitoring

ActiMyo is a movement Holter monitor, composed of a tri-axial accelerometer, a gyroscope and a magnetometer, which is being developed in France at the Institute of Myology in collaboration with SYSNAV enterprise. The measuring principle is based on the use of micro-electromechanical systems (MEMS) inertial sensors and magnetometers operated through magneto-inertial equations. The system is sufficiently light (38gr and 43x33x20mm) and non-intrusive (completely wireless) to be worn as a watch and should not limit movement. It allows continuous daily recording of linear accelerations, angular velocity and the magnetic field, all in three-dimensional space. From these data, a variable representing upper limb activity level is computed. ActiMyo can be used by ambulant and non-ambulant patients, and has already been used in a pilot study in very weak patients. This device will be worn on the dominant wrist of patients and the second device will be fixed on the wheelchair. It will measure physical activity of upper limbs. [APPENDIX 4, page 57].

The accelerometer will be given at the inclusion visit. The recording will be performed continuously over the duration of the participation to the study. The data can be transferred via an Internet cloud, if the patient has Internet access. Otherwise, the USB drive from de device has to be changed every 3 months. Therefore, the device has to be sent to the Institute of Myology after 3 months of use. USB key will be changed and device sent back to the patient for 3 months of recording before his next visit to his center where USB key will be changed another time.

Data analysis will take place at the Institute of Myology.

6.2.9. NMR Imaging (MRI)

MRI assessments will be performed in centers where available, i.e. Paris and Strasbourg.

In this protocol, the aims of the imaging investigation are to track over time

- The muscle volume changes
- The intramuscular fatty infiltration progression
- Indices of disease activity

in SMA patients starting at the age of 4.

Imaging examination will be repeated every 12 months on the dominant upper arm and on the two lower limbs for all patients. MRI assessments will be performed without any sedation.

Muscle and spinal cord characterization will be performed by NMR imaging at 3T.

To separate fat and water contributions to the NMR signal, 3point Dixon gradient-echo imaging will be performed with spatial resolution of 1x1x5mm^3 in the triceps and biceps at arm level (mid-humerus) and flexor and extensor groups at forearm level (proximal third of radius-cubitus). Dixon imaging will also be performed in quadriceps, biceps femoris, semi-tendinosus, semi-membranous at the thigh level (mid-thigh), and triceps suralis, tibialis anterior and peroneus lateralis at the leg level (proximal third). Selection of echo times for this method is dependent on the precise field strength of the scanner: they will be chosen to acquire 3 images where the water and fat contributions are respectively in phase, out of phase and in phase. Percentage of fat signal maps will be generated to quantify the extent of muscle fatty degenerative changes. The out of phase images will be used to measure muscle volumes using an interactive segmentation software developed at the Institute of Myology.

Disease activity will be evaluated using water T2 mapping in moderately infiltrated muscle (% fat signal <60% and >30%) on targeted regions (arm, forearm, thighs and legs). The following muscles or muscle groups will be evaluated in all patients: triceps and biceps at the arm level (mid-humerus) and flexor and extensor groups at the forearm level (proximal third of radius-cubitus). T2 mapping will also be performed in quadriceps, biceps femoris, semi-tendinosus, semi-membranous at the thigh level (mid-thigh), and triceps suralis, tibialis anterior and peroneus lateralis at the leg level (proximal third). 2D multi-slices multi-spin echo images (at least a train of 17 echoes) will be acquired with a spatial resolution of 1.4x1.4x10 mm^3 (4 min per target region). B1 maps will also be acquired with a XFL sequence to consider the effects of B1 inhomogeneities (30s per target region). Parametric maps of muscle water T2 will be derived from these two sequences by a tri-exponential fit of multi-echo signal.

Imaging of the spinal cord (cervical level) will be performed by acquiring two additional MRI sequences: one of these sequences is used for imaging grey-white contrast, the other for imaging the nerve roots. Both these sequences are commercially available sequences used in clinical routine and introduce no SAR issues. Due to an improved imaging protocol, there will be no impact on the total acquisition time.

Participating centers will be trained on-site by a sub-contracting SME, CRIS. This SME has an application specialist permanently based at the Institute of Myology. He is particularly qualified in teaching such protocols, training technologists on-site and performing validation tests and quality controls.

Site validation will include scanning of standardized phantoms and a normal volunteer scan.

Great care will be taken to guarantee reproducible patient positioning. An adjustable leg holder will be provided to sites, if they do not have any.

Central analysis of imaging data will be performed at the Institute of Myology, using dedicated image processing tools that were developed at the NMR laboratory.

6.2.10. Electrophysiology measurement

Assessment of the electrical activity produced by skeletal muscles will be performed as indicated in the schedule of assessment. The measured parameters will be the Compound Motor action Potential Amplitude (CMAP) and research of decrement.

Compound Motor Action Potential (CMAP): The CMAP idealizes the summation of a group of almost simultaneous action potentials from several muscle fibers in the same area. These are usually evoked by stimulation of the motor nerve.

Decrement search: Decrement in CMAP amplitude in response to repetitive nerve stimulation

6.2.11. Quality of Life Assessment

Pediatric Quality of Life Inventory™ (PedsQL™) is a modular instrument designed to measure health-related quality of life and disease-specific symptoms in children and adolescents ages 2-18. The instrument integrates seamlessly generic core scales and disease-specific modules into a single measurement system. The PedsQL™ Generic Core Scale includes 23 items that can be completed in around 4 min and considers developmentally different populations (ages 2-26+; Parent-Report for Toddler for ages 2-4 years; Parent-Report for Young Child for ages 5-7 and Young Adult Self-Report for all other ages will be used). It covers physical, emotional, social and school functioning aspects to assess and summarizes these scales into three main summary scores, total scale score, physical health summary score and psychosocial health summary score.

PedsQL™ Neuromuscular Module encompasses three scales, 17 items related to disease process, 3 items related to communication and 5 items related to family financial and social support (ages 2-18: Parent-Report for Toddler for ages 2-4 years; Parent-Report for Young Child for ages 5-7 and Adolescent Child Self-Report for all other ages will be used).

PedsQL™ assessments will be performed in an interviewer mode if the patient is not able to physically complete the questionnaires, regardless of their age. PedsQL™ has been used and validated in the SMA population (IANNACCONE AND AMERICAN SPINAL MUSCULAR ATROPHY RANDOMIZED TRIALS 2002; IANNACCONE *et al.* 2009; DUNAWAY *et al.* 2010) that captures information on the signs of SMA via its parents version for ages 2-4 years neuromuscular specific module and patient's perspective via its self-reported module. [APPENDIX 6, page **Erreur ! Signet non défini.**]

Health Utilities Inc (HUI2): It specializes in preference-based (utility) measures of health-related quality of life for use in i) describing treatment processes and outcomes in clinical studies; ii) economic evaluations of health care programs; iii) the measurement and monitoring of population health. The HUI2 is the only preference based multi-attribute health related quality of life instrument specifically developed for use with children. It consists of seven dimensions (sensation, mobility, emotion, cognition, self-care, pain and fertility), each of which has between three and five levels. The levels range from "normal functioning for age" to "extreme disability". [APPENDIX 6, page 60]

The HUI2 is a rather generic pediatric quality of life questionnaire that allows measuring the health detriment of SMA patients, allowing also the comparison to healthy individuals and other diseases. HTA agencies (such as NICE, PBAC, SMC) specifically require this questionnaire to assess cost effectiveness of new treatments in pediatric population. On the other hand health authority agencies do acknowledge that this is a generic questionnaire that can be used both in babies and younger children. The use of proxies (parents or Drs.) is also acceptable as babies are not able to value their own health.

The fertility attribute is optional and not part of the standard HUI23-15Q questionnaire, this question will be removed from the forms.

6.2.12. Biological Analysis

All biological analysis will be performed in central labs designated by Roche. For sampling procedures, storage conditions, and shipment instructions, see the separate laboratory manual. Samples will be stored at Roche sample storage facilities and will be destroyed no longer than 5 years after the date of final lock of the clinical database and maybe used for additional exploratory analysis/assay development with respect to SMA severity or progression

Clinical Genotyping:

DNA extraction: 3 ml whole blood sample will be taken from every subject at the inclusion visit in K3 EDTA tube. The DNA will be used to determine the copy number of *SMN2*. The DNA may also be used to study the sequence and genomic region of SMN, genes influencing the disease severity or progression of SMA, to confirm the *SMN1* mutation or deletion and study of exploratory biomarkers related to SMA.

Dynamic Biomarkers:

All the following samples should be taken from every subject at each visit, at the same time of day and under the same food condition (fasting or fed).

RNA isolation: 2.5 ml of whole blood will be collected in PAXgene vacutainers at each visit to determine the relative amounts of *SMN1* and *SMN2* and splice forms thereof. In addition, housekeeping genes for the quantitative analysis of RNA will be measured and mRNA may be measured to study pathways related to SMA disease progression and severity.

SMN protein analysis: 2 ml of whole blood will be collected in p800 tubes (Becton-Dickson) to analyze the SMN protein in the blood of the patients.

Exploratory biomarker related to SMA (e.g. muscle damage, IGF pathway or exploratory profiling technologies to better characterize the disease progression and severity): 6 ml of whole blood will be collected in a plain tube without EDTA

And additional 3 ml whole blood sample will be taken once at V3, V4 or V5 visit in K3 EDTA tube for DNA extraction. The DNA will be used to longitudinally study exploratory biomarkers.

Regulatory aspects in blood sampling:

According to the European Commission[1], in pediatric population, the trial-related blood loss should not exceed 1% of total volume of blood at any single time. The total volume of blood is estimated at 80 to 90 ml/kg body weight; 1% is 0.8 to 0.9 ml blood per kg body weight. The Table 2 summarizes volume of blood

[1] Ethical considerations for clinical trials on medicinal products with the paediatric population - Recommendations of the ad hoc group for the development of implementing guidelines for Directive 2001/20/EC relating to good clinical practice in the conduct of clinical trials on medicinal products for human use - 2008

sampling and the order of priority if blood volume exceeds the maximum amount to be drawn. For children below than 19 kg, the sampling for exploratory biomarkers will not be performed.

Table 2 : Blood sampling volume (ml)

Prioritization	Blood sampling	Inclusion	M6	M12	M18	M24
**1**	RNA Isolation	2.5	2.5	2.5	2.5	2.5
**2**	DNA Extraction	3.0				
**3**	SMN protein analysis	2.0	2.0	2.0	2.0	2.0
**4**	Exploratory biomarkers	6.0	6.0	6.0	6.0	6.0
**5**	DNA Extraction for exploratory biomarkers*			3.0*	3.0*	3.0*
	Total	13.5	10.5	13.5*	13.5*	13.5*

* Sample will be taken once, at V3, V4 or V5

6.3. END OF THE STUDY AND PREMATURE DISCONTINUATION

Patients will end the study after 24 months of follow up.

If desired, a patient may, at any time, stop its participation. It will not change the quality of care or his relationship with his doctor. Unless otherwise specified by the patient, data collected to date for patients who discontinue early will be part of the analysis.

For any discontinuation, the investigator will obtain all the required details and document the date of and the reason for the discontinuation in the CRF. This should be reported including the following reasons:

- Refusal of the patient to continue its participation in the trial
- Lost to follow-up

If a participant does not attend the follow up visits, the investigator in charge will contact him to establish his state at this part of the study. If unable to contact the patient, it will be considered lost to follow-up of the date of last visit.

Lastly, as soon as a therapeutic clinical trial or an open access program begins, patients could discontinue the natural history in order to be able to be included in the therapeutic study or the open-access program.

7. DATA MANAGEMENT AND STATISTICAL ANALYSIS

7.1. DATA MANAGEMENT

AIM or its designee will be responsible for:

- Database creation and validation

- Data entry, editing and quality checking of the data; A comprehensive validation check program will verify the data. Discrepancies will be generated automatically in the system at the point of entry or added manually for resolution
- Data extraction and transfer
- AIM will produce a Data Handling Manual that describes the quality checking to be performed on the data.
- System backups for data stored by AIM and records retention for the study data will be consistent with the AIM’s standard procedures
- CRF review and data validation

Prior to finalizing and locking the database, the appropriate medical and statistical personnel at AIM or its designee will make all decisions regarding inclusion/exclusion of data. Any and all exclusions will be documented in patient listings, summary tabulations and any other clinical trial results reported.

7.2. STATISTICAL ANALYSIS

The study's data analyses are designed primarily to characterize the progression of disease in patients with SMA type 2 and type 3 over a period of 24 months with the goal of exploring suitable outcome measure(s) for future treatment studies. One interim analysis will be performed after 40 patients have completed their 6 months follow up visit and reports thereof will be prepared. A final report will summarize findings after all patients have completed their follow-up.

The time course, the within and between variability, and changes from baseline of the primary, secondary and exploratory endpoints will be analyzed and displayed graphically. Furthermore, multivariate methods such as covariance-structure analysis will be applied to detect dependencies, relation and possible patterns between various outcome measures. The study also seeks to identify prognostic variables of disease progression. Analyses will be performed both overall and by age cohort (2-5 years, and 6-30 years) and by ambulatory status or by choosing ‘age’ and ‘ambulatory status’ as covariates, among others. Further details of the appropriate variable selection method, its analysis and additional statistical methods that will be used are described in the statistical analysis plan.

8. CHANGES IN THE CONDUCT OF THE STUDY

8.1. PROTOCOL AMENDMENTS

Changes to this protocol will be effected through amendments issued after mutual agreement of the Principal Investigator(s) and AIM. The Coordinating Investigator and AIM will sign the amendments. In Europe, when applicable, amendments are submitted to Health Authorities, the Independent Ethics Committee/Institutional Review Board (IEC/IRB) and any other committees by AIM, its representative or the Principal Investigator according to local regulations.

Authorization / approval will be required before implementation of any substantial modification to the protocol which could significantly affect the safety of patients, the scope of the investigation, the scientific

quality of the study or any other aspect of the study. Other changes will be provided when required by local laws to IEC/IRB and any other committees for information only.

An amendment intended to eliminate an apparent immediate hazard to patients may be implemented immediately. Such amendment must be notified as soon as possible to Regulatory Authorities IEC/IRB and any other committees as locally required for authorization / approval.

8.2. PREMATURE STUDY TERMINATION

The Coordinating Investigator and AIM reserve the right to terminate the study at any time. Should this become necessary, the procedures will be agreed upon after consultation between the parts. If the study needs to be terminated, AIM and the Principal Investigators will ensure that adequate consideration is given to the protection of the patients. AIM will notify the Health Authorities and the IEC/IRB and any other committees of the premature study termination according to local regulations.

Should the study be prematurely stopped or put “on-hold” upon Health Authorities’ decision, AIM will inform immediately the Principal Investigators and the other authorities in written including measures to be implemented.

Any Deviations, Violations, Potential serious breaches, urgent safety measures will be recorded.

The sponsor will be notified immediately of any case where the above definition applies during the trial conduct phase.

9. ETHICAL CONSIDERATIONS

9.1. INDEPENDENT ETHICS COMMITTEE/INSTITUTIONAL REVIEW BOARD

Before starting the study, the protocol, the written patient information sheet and informed consent form, and any other document specifically requested must be reviewed and approved by an IEC/IRB complying with the requirements of relevant local law.

Before enrollment of patients, AIM, its representative and/or the Principal Investigators must obtain from the IEC/IRB:

- a written authorization / approval,
- the list of members having participated in the meeting including their qualification.

In addition, IEC/IRB written approval must be obtained by AIM, its representative and/or the Principal Investigators for protocol amendment as described in §8.1.

9.2. INFORMED CONSENT

The Principal Investigators or his / her delegate will obtain a voluntary written consent from both parents or legal guardian(s) of each patient after an appropriate explanation of the aims, methods, anticipated benefits, risks and any other aspect of the study relevant to the patient’s decision to participate. Consent forms and all verbal study related information must be in a language fully comprehensible to the parents or legal guardian(s). Special information will be adjusted to patients below 10 years of age.

Patients and their parents or legal guardians will be informed that they are free not to participate in the study and that they may withdraw consent to participate at any time. They will be told which alternative treatments are available if they refuse to take part and that such refusal will not prejudice future treatment.

Patients and their parents or legal guardians will be informed that their records, including medical history, may be examined by competent authorities and authorized persons but that personal information will be treated as strictly confidential and will not be publicly available.

A written “Parents or legal guardian(s) information sheet” will be given to parents or legal guardian(s) of each patient to complete the verbal information. An adjusted “patient information sheet” will be also given to each patient. This written form should be reviewed orally with the patient and their parents or legal guardian(s). Patients or their parents or legal guardian(s) must be given ample opportunity to inquire about details of the study.

The information sheets will explain that the data collected for this study will be stored in a computer database, with confidentiality maintained in accordance with national data legislation.

Informed consent shall be documented by the use of a written consent form approved by the IEC/IRB and signed and dated by the Principal Investigators or his / her delegate and both parents or legal guardian(s) of the patients and by the patient himself when possible before any exposure to a study-related procedure, including screening tests for eligibility.

Any new version of the informed consent form (ICF) will be signed by all ongoing patients.

A copy of each signed informed consent form must be given to the patient. The originals are filed at the study site in the Investigator Site File.

9.3. DATA PRIVACY

Every effort to protect patient privacy will be made throughout the duration of the study. For all study data collected and documented on the CRFs, patients will be identified by a unique patient identification number only. Only investigational site personnel, AIM and Roche, or its designee, will have access to study records. All study records (including, but not limited to, CRFs) will be maintained in a secure location with restricted access. The results from this study may be presented at scientific meetings and/or published in scientific journals. Identifiers that could potentially identify a patient will not be used in any such meetings or published articles.

The Investigators should keep a patient identification log showing codes, names and addresses of all patients consented. A copy of this log without names and addresses will be filed at AIM after study completion.

9.4. DATA SAFETY MONITORING BOARD

Not applicable

9.5. STEERING COMMITTEE

A steering committee will be constituted by AIM and will include members among the investigators, AIM's teams, Roche and external experts with a maximum of 8 people. The steering committee will be consulted on trial execution issues including subject withdrawal, additional treatment authorization, severity of adverse

events and their relationship with the protocol. The steering committee has an advisory role and can give advice to the Sponsor and the Principal Investigator at any time during the study.

10. ASSESSMENT OF SAFETY

10.1. DEFINITIONS

The adverse event severity grading scale for the NCI CTCAE (v4.03) will be used to assess adverse event severity. Table 3 will be used for assessing severity for adverse events that are not specifically listed in the NCI CTCAE.

Table 3 : Adverse Event Severity Grading Scale

Grade	Severity
1	Mild; asymptomatic or mild symptoms; clinical or diagnostic observations only; or intervention not indicated
2	Moderate; minimal, local, or non-invasive intervention indicated; or limiting age-appropriate instrumental activities of daily living [a]
3	Severe or medically significant, but not immediately life-threatening; hospitalization or prolongation of hospitalization indicated; disabling; or limiting self-care activities of daily living [b,c]
4	Life-threatening consequences or urgent intervention indicated [d]
5	Death related to adverse event [d]

Note:
NCI CTCAE = National Cancer Institute Common Terminology Criteria for Adverse Events.
Note: Based on the NCI CTCAE (v4.03), which can be found at: http://evs.nci.nih.gov/ftp1/CTCAE/CTCAE_4.03_2010-06-14_QuickReference_8.5x11.pdf

[a] Instrumental activities of daily living refer to preparing meals, shopping for groceries or clothes, using the telephone, managing money, etc.
[b] Examples of self-care activities of daily living include bathing, dressing and undressing, feeding one's self, using the toilet, and taking medications, as performed by patients who are not bedridden.
[c] If an event is assessed as a "significant medical event," it must be reported as a serious adverse event.
[d] Grade 4 and 5 events must be reported as serious adverse events.

Adverse event: Any noxious event in a patient included in a biomedical research with or without any relationship with the research.

Serious adverse event: Any untoward medical occurrence that results in death, is life threatening, requires in patient hospitalization or prolongation of existing hospitalization, results in persistent or significant disability/incapacity, is a congenital abnormality/birth defect, is an important medical event that may not be immediately life threatening or result in death or hospitalization, but may jeopardize the patient and may require medical or surgical intervention to prevent one of the outcomes listed above.

New event: Any new safety data may result to a reevaluation of the benefit/risk ratio of the research or may modify the conduct of the research.

10.2. MANAGEMENT OF SERIOUS ADVERSE EVENT

Being a biomedical research whose foreseeable risk added by the research is negligible; no serious adverse effects are expected during the search because on muscle assessments, risks are those related to the

implementation of forced movement (fatigue, muscle pain, cramps). Muscle evaluations will be stopped in the event of occurrence of pain or cramp. Tests could resume after a sufficient period of rest. No serious adverse events are expected due to regular blood sampling as precautionary measures to de-risk this intervention will be implemented (technical and hygienic measures and amounts of blood taken).

Investigators will seek information on adverse events at each patient contact. All AEs, whether reported by the patient or noted by study personnel, will be recorded in the patient's medical record. AEs will then be reported on the AE CRF as follows:

After informed consent has been obtained all adverse events, regardless of relationship to the disease, will be reported until the last follow-up of the last patient in the study

As soon as the investigator is aware, she/he shall report serious adverse events to the sponsor [APPENDIX 7, page 61]. The evolution of each event will be followed until recovery, stabilization, or until it is clearly established by mutual agreement that the event is not related to the current study. Resolution of AEs (with dates) should be documented on the AE CRF form and in the patient's medical record to facilitate source data verification If, after follow-up, return to baseline status or stabilization cannot be established, an explanation should be recorded on the AE CRF form.

11. REGULATORY CONSIDERATIONS

11.1. REGULATORY CONSIDERATIONS

This study will be conducted in accordance with:

- The updated Declaration of Helsinki adopted by the World Medical Association,
- The ICH (International Conference on Harmonization) Good Clinical Practice (GCP) guidelines, and
- The local regulatory requirements.

11.2. REGULATORY APPROVAL / AUTHORIZATION

The regulatory authorization / approval for conducting the study will be obtained from Regulatory Authorities and other national committees in accordance with local regulatory requirements. All approvals must be obtained before a patient is exposed to a study-related procedure, including baseline screening tests for eligibility.

11.3. INVESTIGATORS' OBLIGATIONS

Before the study starts, the Investigators shall provide AIM / or his representative with their signed and updated curriculum vitae and complete a list giving the names, functions and authorized activities of all persons who will exercise any kind of responsibility in carrying out of the study.

The Investigators ensure the quality of the study through strict observance of the protocol, Good Clinical Practice and local regulations. Investigators must ensure that the study has been authorized / approved by all Regulatory Authorities, IEC/IRB and any other committees prior to enrolling patients and on an ongoing basis as locally required.

Investigators are required to obtain written informed consent from each patient before any exposure to a study-related procedure, including baseline screening tests for eligibility. During the study, Investigators must obtain signature of any new version of the ICF by all ongoing patients.

11.4. INSURANCE

AIM certifies having taken out a civil liability insurance policy covering liability with regard to the participants in this study.

11.5. FINANCIAL DISCLOSER

AIM certifies that the present study is totally funded due to a collaboration between AIM and Institut Roche de Recherche et Médecine Translationnelle (Signature of the Letter of Intent signed by AIM and Institut Roche de Recherche et Médecine Translationnelle on going)

12. QUALITY CONTROL AND QUALITY ASSURANCE

12.1. PERIODIC MONITORING

This trial will be monitored. The monitor will contact and visit the Investigators periodically to evaluate study progress, protocol and GCP compliance (including verification of informed consents conformity, following of adverse event (study related or not) and review of the quality, completeness and timeliness of all data in each case report form). For this study, the average frequency of the monitoring visits will be determined according to recruitment rate of each center and the age of enrolled patient (for example, monitoring visits will be more frequent if the patients enrolled are 2-5 years old) with the first visit occurring as soon as possible after the first patient inclusion. Intervals may be adjusted according to patient accruals, protocol changes or site performance.

The Investigators and any study staff member will co-operate with the monitor to ensure that any problems are resolved.

12.2. AUDIT AND INSPECTION

After appropriate notification, the Investigators will make all study-related source data and documents available to a quality assurance auditor mandated by AIM, or to domestic or foreign regulatory inspectors. The main purposes of an audit or inspection are to confirm that the rights and well-being of the patients have been adequately protected, and that all data relevant for the evaluation of the course of the disease have been processed and reported in compliance with GCP and applicable regulatory requirements.

13. DATA HANDLING AND RECORD KEEPING

13.1. SOURCE DATA AND DOCUMENTS

Source data are all information available in original source document or certified copies of source document of any clinical findings, observations, or other activities that are necessary for the reconstruction and evaluation of the study.

The Investigators will record at least the following information in the source documents for all consented patients: patient name, date of birth, sex, medical history, reference to the study, visit dates, concomitant medications, evaluation criteria, nature of adverse events with dates of start and end.

Localization of each source data will be identified on the dedicated form.

When computerized systems are used in the original recording of data, the following criteria should be met:

- documented evidence that the computer system has been validated,
- the system provides adequate security to ensure that only authorized persons can enter/change data and allows audit trail of entries/changes,
- existence of procedure for manual data entry in case of system break down,
- if no electronic signature is in place, the Investigators agree to print out data periodically and to sign the printouts, and
- when possible, compliance with 21 CFR part 11[2].

The Investigators will permit study-related monitoring, audit(s), and regulatory inspection(s), with a direct access to all the required source documents each time it is necessary provided that patient confidentiality is protected.

13.2. CASE REPORT FORMS

All relevant data abstracted from the medical records of qualifying patients will be transcribed from source documents and entered in the CRF designed by AIM. Data generated during the study will also be captured in the CRF. The data will then be double data entered into a validated clinical database. Paper CRFs will be completed by Investigational study site personnel and transmitted to AIM for data entry. CRF completion guidelines will be provided to the Investigational sites by AIM prior to CRF finalization.

All data must be entered in English.

The CRFs should always reflect the latest observations on the patients participating in the study. Therefore, the CRFs are to be completed as soon as possible during or immediately after the patient's visit. To avoid inter observer variability; every effort should be made to ensure that the same individual who made the initial baseline determinations completes all evaluations. The Investigators must verify that all data entries in the CRFs are accurate and correct. If some assessments are not done, or if certain information is not

[2] Title 21 CFR Part 11 of the Code of Federal Regulations deals with the Food and Drug Administration (FDA) guidelines on electronic records and electronic signatures in the United States. Part 11, as it is commonly called, defines the criteria under which electronic records and electronic signatures are considered to be trustworthy, reliable and equivalent to paper records (Title 21 CFR Part 11 Section 11.1 (a)).

available, not applicable, or unknown, the Investigators should indicate this in the CRF. The Investigators will be required to sign the clinical data.

The Monitors will review the CRFs, and evaluate them for completeness and consistency. The CRF will be compared with the source documents to ensure that there are no discrepancies. The extent of source data verification performed by the Monitors is 100 %.

All entries, corrections and alterations are to be made by the Investigator or his/her delegate. The Monitors cannot enter data in the CRFs. Once clinical data of the CRF have been submitted to the central server, corrections to the data fields will be audit trailed, meaning that the reason for change, the name of the person who performed the change, together with time and date will be logged. Roles and rights of the site personnel responsible for entering the clinical data into the CRF will be determined in advance.

If additional corrections are needed, the Monitors or Data Manager(s) will raise a query. The appropriate investigational staff will answer queries sent to the Investigators.

Investigators will maintain copies of the CRFs at the clinical site. For patients who discontinue or terminate from the study, the CRFs will be completed as much as possible, and the reason for the discontinuance or termination clearly and concisely specified on the appropriate CRF.

14. CLINICAL STUDY REPORT AND PUBLICATION

14.1. CLINICAL STUDY REPORT

All relevant data will be reported in a study report which will be prepared by AIM and submitted for comments to Roche and Coordinating Investigator. It will be signed by AIM and Coordinating Investigator. The final report is used for regulatory purposes by AIM according to local regulations and provided to each Investigator and collaborators once finalized.

14.2. CONFIDENTIALITY OF STUDY DATA

All information that is part and will be obtained in this study is strictly confidential. All the people taking part in this research are bound to observe the confidentiality of this information. Neither the Investigators nor any person working on their behalf may disclose any of the information therein without having obtained prior written authorization from AIM.

14.3. PUBLICATION POLICY

The results of this study will be published or presented at scientific meetings. All manuscripts or abstracts will be submitted to the coordinating investigator and Roche prior to scientific journal or meeting submission for reviewing and agreement of publication. As soon as the last patient is enrolled, the Parties will endeavor publish interim results within 3 to 4 months.

The name and the order of authors will be submitted to Roche and Coordinating investigator approval.

The Parties will publish the full data promptly after the study has been completed. The Parties will assure that any publications or other public disclosures will include Institut Roche de Recherche et Médecine Translationnelle and AFM's Institute of Biotherapies (BIRD) are the funders of the study.

The identity of each patient is confidential and will be protected in any future publications.

15. ARCHIVING

15.1. INVESTIGATOR SITE FILE

In accordance with the ICH GCP standards, Investigators are responsible for on-site storage and maintenance of all records pertaining to the study for the maximum period of time required by local requirements.

No study site document may be destroyed without prior written agreement between the Investigators and AIM. AIM must be notified if the Investigators assign the study documentation to another party or moves it to another location.

If the Investigators cannot guarantee this archiving requirement on site for any or all of the documents, special arrangements must be made between the Investigators and AIM to store the documents in a sealed container off-site so they can be returned sealed to the Investigators in case of an audit/inspection.

15.2. TRIAL MASTER FILE

AIM will archive the Trial Master File (TMF) in accordance with GCP and applicable regulatory requirements, and will inform the Investigators when the archiving of the study documentation is no longer required.

BIBLIOGRAPHY

A.M. Seferian, A. M., A. Canal, V. Decostre, O. Diebate, A-G. Le Moing, T. Gidaro, N. Deconinck, F. Van Parys, W. Vereecke, S. Wittevrongel, M. Annoussamy, M. Mayer, K. Maincent, J-M. Cuisset, V. Tiffereau, S. Denis, V. Jousten, S. Quijano-Roy, T. Voit, J.-Y. Hogrel, Laurent Servais, Submitted Upper Limb Evaluation and One-Year Follow Up of Non-Ambulant Patients with Spinal Muscular Atrophy: An Observational Multicenter Trial. PLoS One.

Arkblad, E., M. Tulinius, A. K. Kroksmark, M. Henricsson and N. Darin, 2009 A population-based study of genotypic and phenotypic variability in children with spinal muscular atrophy. Acta Paediatr 98**:** 865-872.

Berard, C., C. Payan, I. Hodgkinson, J. Fermanian and M. F. M. C. S. Group, 2005 A motor function measure for neuromuscular diseases. Construction and validation study. Neuromuscul Disord 15**:** 463-470.

Buj-Bello, A., F. Fougerousse, Y. Schwab, N. Messaddeq, D. Spehner *et al.*, 2008 AAV-mediated intramuscular delivery of myotubularin corrects the myotubular myopathy phenotype in targeted murine muscle and suggests a function in plasma membrane homeostasis. Hum Mol Genet 17**:** 2132-2143.

de Lattre, C., C. Payan, C. Vuillerot, P. Rippert, D. de Castro *et al.*, 2013 Motor Function Measure: Validation of a Short Form for Young Children With Neuromuscular Diseases. Arch Phys Med Rehabil.

Dunaway, S., J. Montes, M. Montgomery, V. Battista, B. Koo *et al.*, 2010 Reliability of telephone administration of the PedsQL Generic Quality of Life Inventory and Neuromuscular Module in spinal muscular atrophy (SMA). Neuromuscul Disord 20**:** 162-165.

Emery, A. E., 1991 Population frequencies of inherited neuromuscular diseases--a world survey. Neuromuscul Disord 1**:** 19-29.

Haaker, G., and A. Fujak, 2013 Proximal spinal muscular atrophy: current orthopedic perspective. Appl Clin Genet 6**:** 113-120.

Iannaccone, S. T., and G. American Spinal Muscular Atrophy Randomized Trials, 2002 Outcome measures for pediatric spinal muscular atrophy. Arch Neurol 59**:** 1445-1450.

Iannaccone, S. T., L. S. Hynan, A. Morton, R. Buchanan, C. A. Limbers *et al.*, 2009 The PedsQL in pediatric patients with Spinal Muscular Atrophy: feasibility, reliability, and validity of the Pediatric Quality of Life Inventory Generic Core Scales and Neuromuscular Module. Neuromuscul Disord 19**:** 805-812.

Lefebvre, S., L. Burglen, S. Reboullet, O. Clermont, P. Burlet *et al.*, 1995 Identification and characterization of a spinal muscular atrophy-determining gene. Cell 80**:** 155-165.

Lofaso, F., F. Nicot, M. Lejaille, L. Falaize, A. Louis *et al.*, 2006 Sniff nasal inspiratory pressure: what is the optimal number of sniffs? Eur Respir J 27**:** 980-982.

McDonald, C. M., E. K. Henricson, J. J. Han, R. T. Abresch, A. Nicorici *et al.*, 2010 The 6-minute walk test as a new outcome measure in Duchenne muscular dystrophy. Muscle Nerve 41**:** 500-510.

Melki, J., S. Abdelhak, P. Sheth, M. F. Bachelot, P. Burlet *et al.*, 1990 Gene for chronic proximal spinal muscular atrophies maps to chromosome 5q. Nature 344**:** 767-768.

Mercuri, E., E. Bertini and S. T. Iannaccone, 2012 Childhood spinal muscular atrophy: controversies and challenges. Lancet Neurol 11**:** 443-452.

Merlini, L., S. B. Stagni, E. Marri and C. Granata, 1992 Epidemiology of neuromuscular disorders in the under-20 population in Bologna Province, Italy. Neuromuscul Disord 2**:** 197-200.

Munsat, T. L., and K. E. Davies, 1992 International SMA consortium meeting. (26-28 June 1992, Bonn, Germany). Neuromuscul Disord 2**:** 423-428.

Prigent, H., M. Lejaille, L. Falaize, A. Louis, M. Ruquet *et al.*, 2004 Assessing inspiratory muscle strength by sniff nasal inspiratory pressure. Neurocrit Care 1**:** 475-478.

Rudnik-Schoneborn, S., I. Hausmanowa-Petrusewicz, J. Borkowska and K. Zerres, 2001 The predictive value of achieved motor milestones assessed in 441 patients with infantile spinal muscular atrophy types II and III. Eur Neurol 45**:** 174-181.

Russman, B. S., C. R. Buncher, M. White, F. J. Samaha and S. T. Iannaccone, 1996 Function changes in spinal muscular atrophy II and III. The DCN/SMA Group. Neurology 47**:** 973-976.

Schroth, M. K., 2009 Special considerations in the respiratory management of spinal muscular atrophy. Pediatrics 123 Suppl 4**:** S245-249.

Servais, L., N. Deconinck, A. Moraux, M. Benali, A. Canal *et al.*, 2013 Innovative methods to assess upper limb strength and function in non-ambulant Duchenne patients. Neuromuscul Disord 23**:** 139-148.

APPENDIX 1. SMA QUESTIONNAIRE

Quality of life:

- In the last year, number of work days missing due to caring for a child with SMA: |__|__|__|
- In the last year, number of school or work days missing due to the SMA: |__|__|__|
- In the last year, number of family and community events missed due to the SMA: |__|__|__|

Psychomotor development

Milestones	Acquisition			Loss				
	Yes	No	Age (month)	Yes	No	Age	Years	Months
Ability to have head control	☐	☐	|__|__|	☐	☐	|__|__|.|__|	☐	☐
Ability to roll to one side	☐	☐	|__|__|	☐	☐	|__|__|.|__|	☐	☐
Ability to roll over completely	☐	☐	|__|__|	☐	☐	|__|__|.|__|	☐	☐
Ability to sit independently when placed	☐	☐	|__|__|	☐	☐	|__|__|.|__|	☐	☐
Ability to get in a sitting position	☐	☐	|__|__|	☐	☐	|__|__|.|__|	☐	☐
Ability to crawl	☐	☐	|__|__|	☐	☐	|__|__|.|__|	☐	☐
Ability to stand up	☐	☐	|__|__|	☐	☐	|__|__|.|__|	☐	☐
Acquisition of walking with help	☐	☐	|__|__|	☐	☐	|__|__|.|__|	☐	☐
Acquisition of autonomy walking	☐	☐	|__|__|	☐	☐	|__|__|.|__|	☐	☐
Ability to run	☐	☐	|__|__|	☐	☐	|__|__|.|__|	☐	☐
Ability to jump	☐	☐	|__|__|	☐	☐	|__|__|.|__|	☐	☐
Climbing stairs with banister	☐	☐	|__|__|	☐	☐	|__|__|.|__|	☐	☐
Climbing stairs without banister	☐	☐	|__|__|	☐	☐	|__|__|.|__|	☐	☐
Ability to bring hands to mouth	☐	☐	|__|__|	☐	☐	|__|__|.|__|	☐	☐
Ability to feed with finger	☐	☐	|__|__|	☐	☐	|__|__|.|__|	☐	☐
Ability to use utensils to eat	☐	☐	|__|__|	☐	☐	|__|__|.|__|	☐	☐
Ability to self-dress	☐	☐	|__|__|	☐	☐	|__|__|.|__|	☐	☐

**Mental retardation**	Yes: ☐ No: ☐
IQ total (formally evaluated)	Yes: ☐ No: ☐
	If yes, |__|__|.|__| Years ☐ Months ☐
	Age: |__|__|__|
	Value:
Method/Test:	____
For children: School attendance	Yes: ☐ No: ☐<br>If yes, which level: ______________________________
For adults: Employment status	☐ Employed/self-employed full-time<br>☐ Employed/self-employed part-time<br>☐ Student<br>☐ Unemployed
Education status	☐ Elementary School<br>☐ High School<br>☐ College<br>☐ Post Graduate
For teenagers & adults: Care level	☐ Self-Carer<br>☐ Caregiver Required

Respiratory function assessment

- Breathing support: Yes: ☐ No: ☐
    - If yes, Number of hours of ventilation per day: |__|__| hours

      Type of breathing support:

	Currently Using	If currently using, how often?	Used in Past but not now	Never used
Non-invasive CPAP (continuous positive airway pressure)	☐	☐ Daytime<br>☐ Night-time<br>☐ As Needed	☐	☐
Non-invasive BiPAP (bi-level positive airway pressure)	☐	☐ Daytime<br>☐ Night-time<br>☐ As Needed	☐	☐
NPV (negative pressure ventilation) aka Iron lung	☐	☐ Daytime<br>☐ Night-time<br>☐ As Needed	☐	☐
Sip and Puff	☐	☐ Daytime<br>☐ Night-time<br>☐ As Needed	☐	☐
Ventilator (with tracheostomy)	☐	☐ Daytime<br>☐ Night-time<br>☐ As Needed	☐	☐
Other (please specify)	☐	☐ Daytime<br>☐ Night-time<br>☐ As Needed	☐	☐

- Respiratory lower tract infection: Yes: ☐ No: ☐
    - If yes, mean number per year: |__|__|
- Sleep apnoea: Yes: ☐ No: ☐
- Vital capacity assessed: Yes: ☐ No: ☐
    - If yes, date of last assessment: |__|__| |__|__| |__|__|__|__|

      Value: |__|__|__| % of predicted value

Feeding status

- Difficulty for feeding: Yes: ☐ No: ☐ Unknown: ☐
  - If yes, Date of last assessment: |__|__||__|__||__|__|__|__|
    - ☐ Difficulties for sucking
      - ☐ At the present time
      - ☐ Other period, specify: ______________________________________
    - ☐ Difficulties for swallowing
    - ☐ Difficulties for chewing
    - ☐ Reflux/vomiting
    - ☐ Other: ____
- Current need of a feeding tube:Yes: ☐ No: ☐ Unknown: ☐
- Past need of a feeding tube: Yes: ☐ No: ☐ Unknown: ☐
  - If yes, Naso-Gastric tube: Yes: ☐ No: ☐

    If yes, Date of onset: |__|__||__|__||__|__|__|__|

    Date of end: |__|__||__|__||__|__|__|__|

    Percentage of oral calories intake: |__|__|__|

    Gastrostomy: Yes: ☐ No: ☐

    If yes, Date of onset: |__|__||__|__||__|__|__|__|

    Date of end: |__|__||__|__||__|__|__|__|

    Percentage of oral calories intake: |__|__|__|

Orthopaedic status

- **Scoliosis:** Yes: ☐ No: ☐
  - Arthrodesis: Yes: ☐ No: ☐

    If Yes, Date: |__|__||__|__||__|__|__|__|

    If No, Scoliosis Angle: ☐ < 30°

    ☐ 30-45°

    ☐ > 45°

- **Fracture:** Yes: ☐ No: ☐
  - If yes, specify:

Localization	Date
___________________________________	|__|__||__|__||__|__|__|__|
___________________________________	|__|__||__|__||__|__|__|__|

- **Assistive device:** Yes: ☐ No: ☐

	Day Time Only	Night Time only	All hours	Never	Used in past but not anymore	Other (please specify)
Corset	☐	☐	☐	☐	☐	☐
Lumbar belt	☐	☐	☐	☐	☐	☐
Foot/Ankle Orthotics (i.e. DFO, AFO, etc.)	☐	☐	☐	☐	☐	☐
Hand/Wrist Splint	☐	☐	☐	☐	☐	☐
Ankle Splint	☐	☐	☐	☐	☐	☐
Stander	☐	☐	☐	☐	☐	☐
Cane	☐	☐	☐	☐	☐	☐
Walker	☐	☐	☐	☐	☐	☐
Manual Wheelchair	☐	☐	☐	☐	☐	☐
Power Wheelchair	☐	☐	☐	☐	☐	☐
Communication Device, please specify: _________________________ _________________________________	☐	☐	☐	☐	☐	☐
Other (acupuncture, massage, etc.), please specify:____________________ _________________________________	☐	☐	☐	☐	☐	☐

APPENDIX 2. MUSCLE STRENGTH ASSESSMENT

MYOGRIP

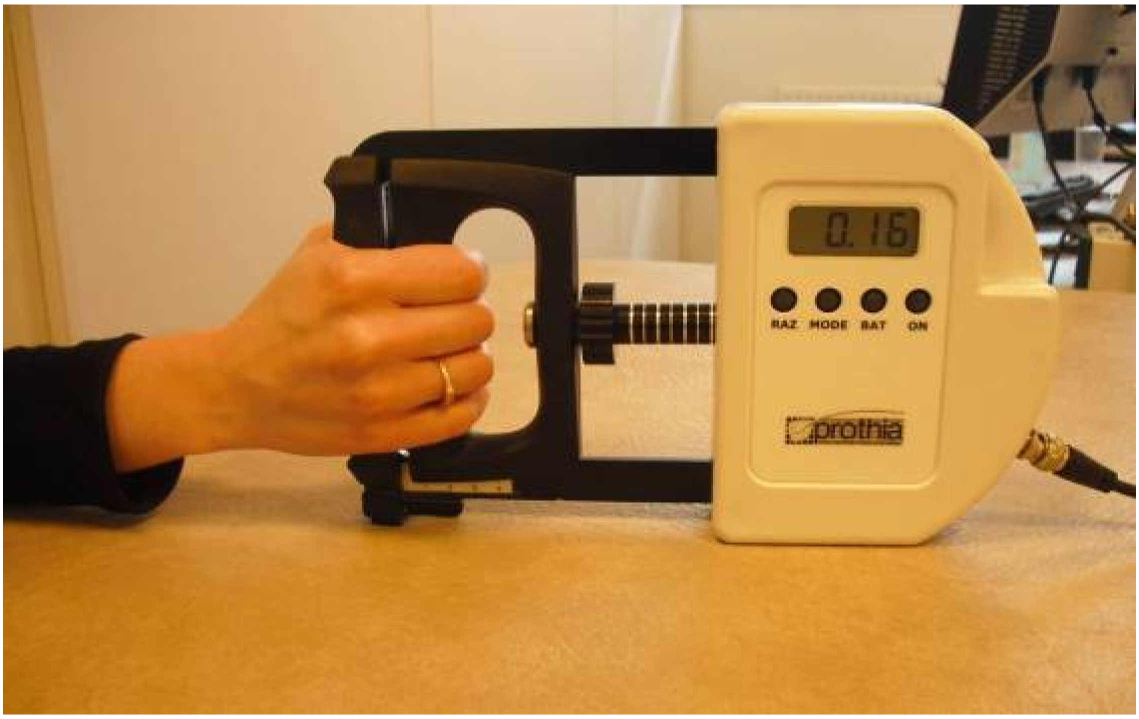

MYOPINCH

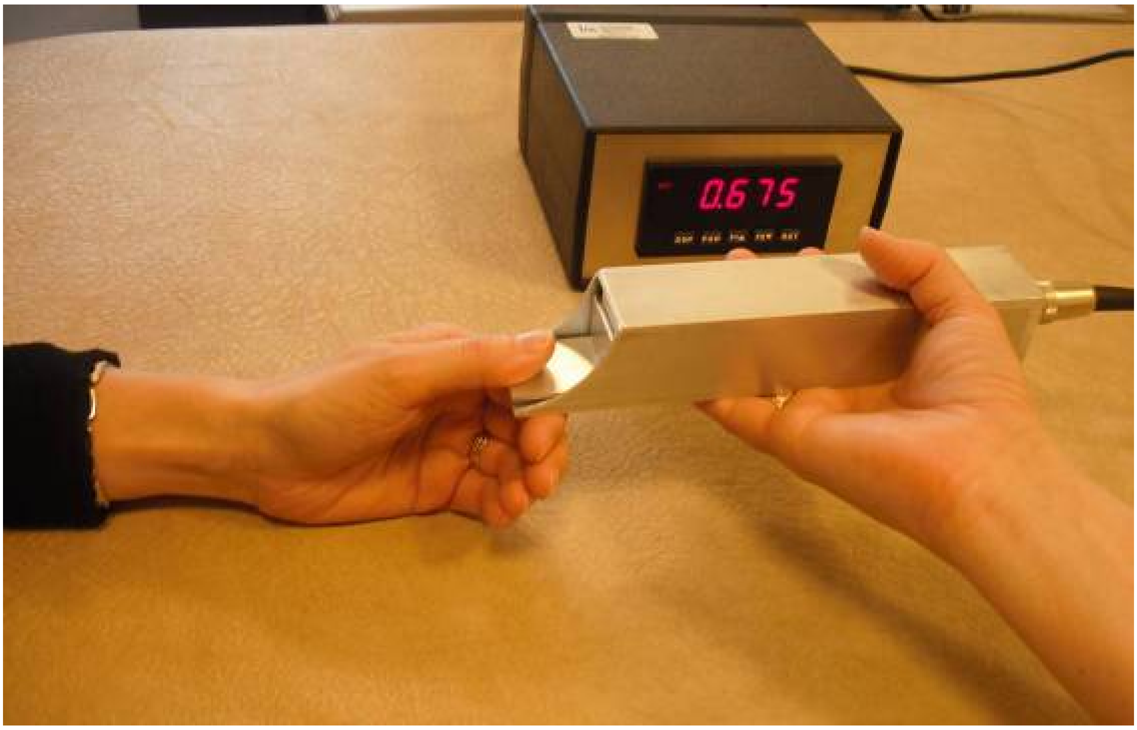

APPENDIX 3. MOTOR FUNCTION ASSESSMENT

MOVIPLATE

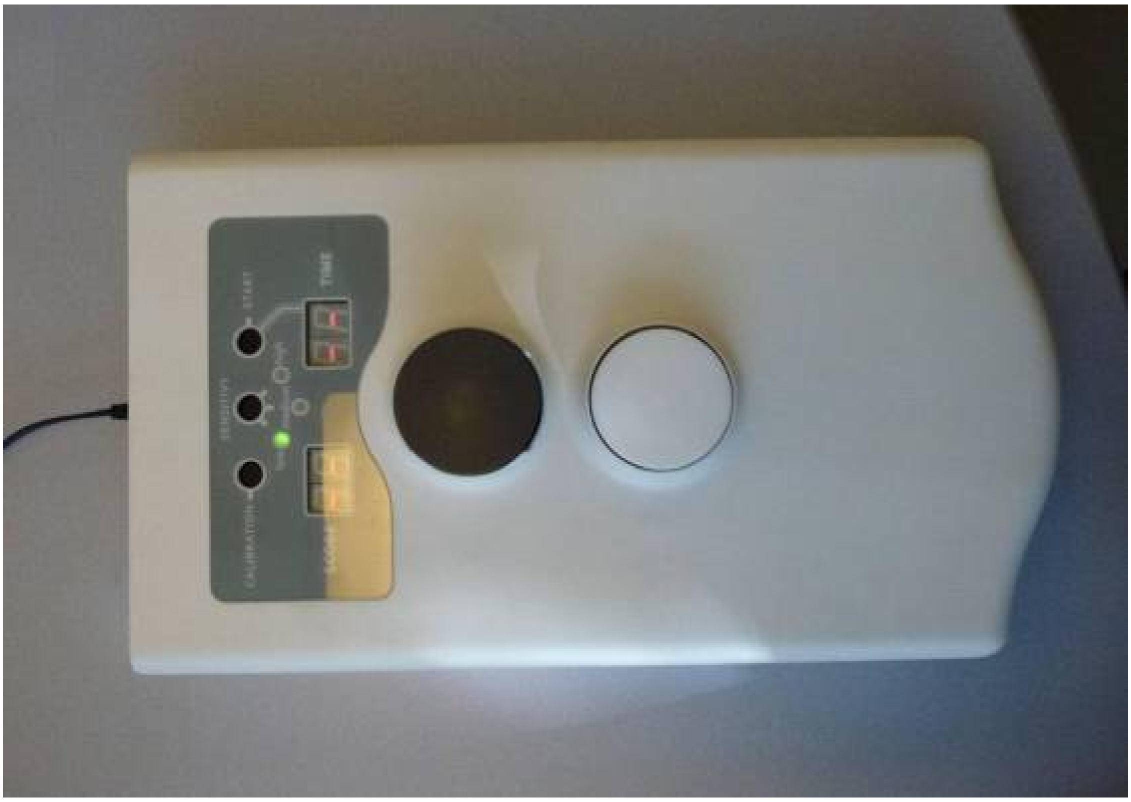

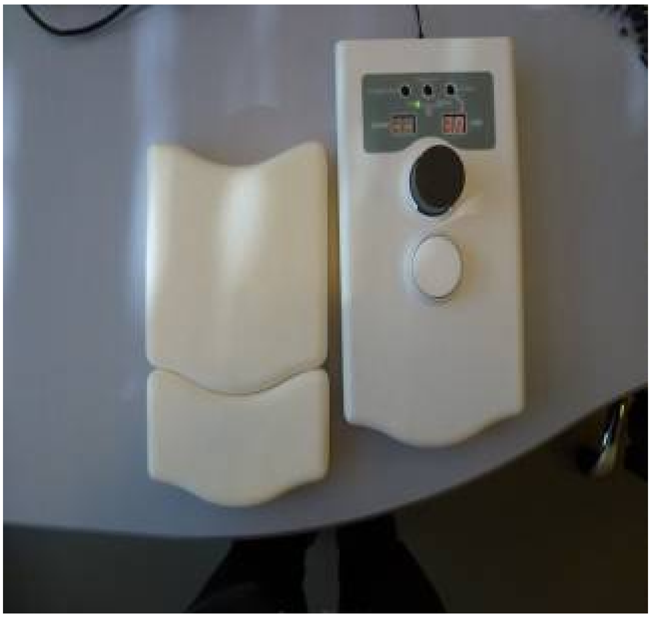

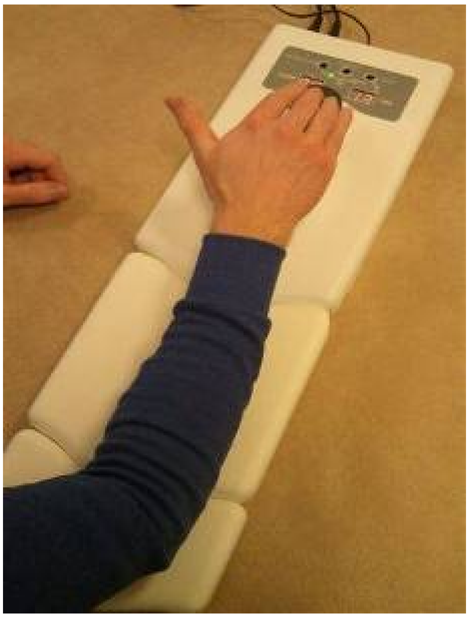

MOTOR FUNCTION MEASURE (MFM)

MOTOR FUNCTION MEASURE 20 (MFM 20)

Scoring :	D1	D2	D3
**1.** SUPINE, HEAD IN MIDLINE POSITION: holds the head for 5 seconds in midline position and turns it completely from one side to the other.<br>*Comment :* ....<br>...		0❑<br>1❑<br>2❑<br>3❑	
**3.** SUPINE: flexes the hip and knee more than 90° by raising the foot from the mat.<br>*Comment :* ....<br>... Side: right ❑ left ❑		0❑<br>1❑<br>2❑<br>3❑	
**4.** SUPINE, LEG SUPPORTED BY EXAMINER: from the plantar flexion, dorsiflexes the foot to at least 90° in relation to the leg.<br>*Comment :* ....<br>...<br>Side: right ❑ left ❑			0❑<br>1❑<br>2❑<br>3❑
**5.** SUPINE: raises the hand and moves it to the opposite shoulder.<br>*Comment :* ....<br>... Side: right ❑ left ❑		0❑<br>1❑<br>2❑<br>3❑	
**6.** SUPINE, LOWER LIMBS HALF-FLEXED, KNEECAPS AT THE ZENITH AND FEET RESTING ON THE MAT: raises the pelvis; the lumbar spine, the pelvis and the thighs are aligned and the feet slightly apart.<br>*Comment :* ....<br>...	0❑<br>1❑<br>2❑<br>3❑		
**7.** SUPINE: turns over into prone and frees both upper limbs from under the body.<br>*Comment :* ....<br>... Side: right ❑ left ❑		0❑<br>1❑<br>2❑<br>3❑	
**9.** SEATED ON THE MAT: without upper limb support, maintains the seated position for 5 seconds and is then capable of maintaining contact between the two hands for 5 seconds.<br>*Comment :* ....<br>...		0❑<br>1❑<br>2❑<br>3❑	
**10.** SEATED ON THE MAT, THE TENNIS BALL PLACED IN FRONT OF THE SUBJECT: without upper limb support, leans forward, touches the ball and sits back again.<br>*Comment :* ....<br>...		0❑<br>1❑<br>2❑<br>3❑	
**11.** SEATED ON THE MAT: stands up without upper limb support.<br>*Comment :* ....<br>...	0❑<br>1❑<br>2❑<br>3❑		
**12.** STANDING: without upper limb support, sits down on the chair with the feet slightly apart.<br>*Comment :* ....<br>...	0❑<br>1❑<br>2❑<br>3❑		

**14.** SEATED ON THE CHAIR OR IN THE WHEELCHAIR, HEAD IN FLEXION: raises the head from the flexion position, the head stays aligned throughout the movement and is maintained raised in midline position for 5 seconds.<br>*Comment :* ....<br>..		0❑<br>1❑<br>2❑<br>3❑	
**18.** SEATED ON THE CHAIR OR IN THE WHEELCHAIR, ONE FINGER PLACED IN THE CENTER OF THE FIXED CD: goes round the edge of the CD with one finger without contact of the hand on the table.<br>*Comment :* ....<br>..Side: right ❑ left ❑			0❑<br>1❑<br>2❑<br>3❑
**21.** SEATED ON THE CHAIR OR IN THE WHEELCHAIR, THE TENNIS BALL ON THE TABLE: picks up the ball, and turns the hand over completely holding the ball.<br>*Comment :* ....<br>..Side: right ❑ left ❑			0❑<br>1❑<br>2❑<br>3❑
**22.** SEATED ON THE CHAIR OR IN THE WHEELCHAIR, ONE FINGER PLACED IN THE CENTER OF THE DIAGRAM BELOW: raises the finger and places it successively on the 8 squares on the diagram without touching the lines.<br>*Comment :* ....<br>..Side: right ❑ left ❑			0❑<br>1❑<br>2❑<br>3❑
**23.** SEATED ON THE CHAIR OR IN THE WHELLCHAIR, UPPER LIMBS ALONG THE TRUNK: places the two forearms and/or the hands on the table at the same time.<br>*Comment :* ....<br>..		0❑<br>1❑<br>2❑<br>3❑	
**24.** SEATED ON THE CHAIR: stands up without upper limb support and with the feet slightly apart.<br>*Comment :* ....<br>..	0❑<br>1❑<br>2❑<br>3❑		
**25.** STANDING WITH UPPER LIMB SUPPORT ON EQUIPMENT: releases the support and maintains a standing position for 5 seconds with the feet slightly apart, the head, trunk and limbs in midline position.<br>*Comment :* ....<br>..	0❑<br>1❑<br>2❑<br>3❑		
**27.** STANDING: without support, touches the floor with one hand and stands up again.<br>*Comment :* ....<br>..	0❑<br>1❑<br>2❑<br>3❑		
**30.** STANDING WITHOUT SUPPORT: runs 10 meters.<br>*Comment :* ....<br>..	0❑<br>1❑<br>2❑<br>3❑		
**32.** STANDING WITHOUT SUPPORT: without upper limb support, manages to squat and gets up twice in a row.<br>*Comment :* ....<br>..	0❑<br>1❑<br>2❑<br>3❑		

**TOTAL**	D1=	D2=	D3=

SUMMARY OF SCORING:

DIMENSION	CALCULATION OF DIMENSION % SCORES
**D1.** Standing and transfers	Total dimension 1 = _______ x 100 = _ _ ._ _ % 8 x 3 24
**D2.** Axial and proximal motor function	Total dimension 2 = _______ x 100 = _ _ ._ _ % 8 x 3 24
**D3.** Distal motor function	Total dimension 3 = _______ x 100 = _ _ ._ _ % 4 x 3 12
**TOTAL SCORE**	Total mark = _______ x 100 = _ _ ._ _ % 20 x 3 60

Patient cooperation : none ❑ medium ❑ optimal ❑

Particularities during this MFM test : :

....

....

......

....

...

MOTOR FUNCTION MEASURE 32 (MFM 32)

**Scoring :**	**D1**	**D2**	**D3**
**1.** SUPINE, HEAD IN MIDLINE POSITION: holds the head for 5 seconds in midline position and turns it completely from one side to the other.<br>*Comment :* ....<br>..		0❑<br>1❑<br>2❑<br>3❑	
**2.** SUPINE : raises the head and maintains the raised position for 5 seconds<br>*Comment :* ....<br>..		0❑<br>1❑<br>2❑<br>3❑	
**3.** SUPINE: flexes the hip and knee more than 90° by raising the foot from the mat.<br>*Comment :* ....<br>..Side :<br>right ❑ left ❑		0❑<br>1❑<br>2❑<br>3❑	
**4.** SUPINE, LEG SUPPORTED BY EXAMINER: from the plantar flexion, dorsiflexes the foot to at least 90° in relation to the leg.<br>*Comment :* ....<br>..<br>Side : right ❑ left ❑			0❑<br>1❑<br>2❑<br>3❑
**5.** SUPINE: raises the hand and moves it to the opposite shoulder.<br>*Comment :* ....<br>..Side :<br>right ❑ left ❑		0❑<br>1❑<br>2❑<br>3❑	
**6.** SUPINE, LOWER LIMBS HALF-FLEXED, KNEECAPS AT THE ZENITH AND FEET RESTING ON THE MAT: raises the pelvis; the lumbar spine, the pelvis and the thighs are aligned and the feet slightly apart.<br>*Comment :* ....<br>..	0❑<br>1❑<br>2❑<br>3❑		
**7.** SUPINE: turns over into prone and frees both upper limbs from under the body.<br>*Comment :* ....<br>.. Side :<br>right ❑ left ❑		0❑<br>1❑<br>2❑<br>3❑	
**8.** SUPINE: without upper limb support sits up on the mat.<br>*Comment :* ....<br>..	0❑<br>1❑<br>2❑<br>3❑		

**9.** SEATED ON THE MAT: without upper limb support, maintains the seated position for 5 seconds and is then capable of maintaining contact between the two hands for 5 seconds.<br>*Comment :* ....<br>..		0❑<br>1❑<br>2❑<br>3❑	
**10.** SEATED ON THE MAT, THE TENNIS BALL PLACED IN FRONT OF THE SUBJECT: without upper limb support, leans forward, touches the ball and sits back again.<br>*Comment :* ....<br>..		0❑<br>1❑<br>2❑<br>3❑	
**11.** SEATED ON THE MAT: stands up without upper limb support.<br>*Comment :* ....<br>..	0❑<br>1❑<br>2❑<br>3❑		
**12.** STANDING: without upper limb support, sits down on the chair with the feet slightly apart.<br>*Comment :* ....<br>..	0❑<br>1❑<br>2❑<br>3❑		
**13.** SEATED ON THE CHAIR: without upper limb support nor leaning against the back of the chair, maintains the seated position for 5 seconds, with the head and trunk in midline position.<br>*Comment :* ....<br>..		0❑<br>1❑<br>2❑<br>3❑	
**14.** SEATED ON THE CHAIR OR IN THE WHEELCHAIR, HEAD IN FLEXION: raises the head from the flexion position, the head stays aligned throughout the movement and is maintained raised in midline position for 5 seconds.<br>*Comment :* ....<br>..		0❑<br>1❑<br>2❑<br>3❑	
**15.**, SEATED ON THE CHAIR OR IN THE WHEELCHAIR, FOREARMS ON THE TABLE, BUT NOT ELBOWS: places both hands on top of the head at the same time while the head and trunk remain in midline position.<br>*Comment :* ....<br>..		0❑<br>1❑<br>2❑<br>3❑	
**16.** SEATED ON THE CHAIR OR IN THE WHEELCHAIR, THE PENCIL ON THE TABLE: reaches the pencil with one hand with the elbow in full extension at the end of the movement.<br>*Comment :* ....<br>.. Side :<br>right ❑ left ❑		0❑<br>1❑<br>2❑<br>3❑	

**17.** SEATED ON THE CHAIR OR IN THE WHEELCHAIR, 10 COINS ON THE TABLE: successively picks up and holds 10 coins in one hand during the 20-second period.<br>*Comment : ....*<br>... Side :<br>right ❑ left ❑			0❑<br>1❑<br>2❑<br>3❑
**18.** SEATED ON THE CHAIR OR IN THE WHEELCHAIR, ONE FINGER PLACED IN THE CENTER OF THE FIXED CD: goes round the edge of the CD with one finger without contact of the hand on the table.<br>*Comment : ....*<br>...Side :<br>right ❑ left ❑			0❑<br>1❑<br>2❑<br>3❑
**19.** SEATED ON THE CHAIR OF IN THE WHEELCHAIR, THE PENCIL ON THE TABLE: picks up the pencil and draws a continuous series of loops inside the frame and over its full length touching the top and bottom line of the frame.<br>**Trial n°1**<br>**Trial n°2**<br>*Comment : ....*<br>...<br>Side : right ❑ left ❑			0❑<br>1❑<br>2❑<br>3❑
**20.** SEATED ON THE CHAIR PR OR IN THE WHEELCHAIR, HOLDING THE SHEET OF PAPER: tears the sheet of paper folded in 4, beginning with the fold.<br>*Comment : ....*<br>...			0❑<br>1❑<br>2❑<br>3❑
**21.** SEATED ON THE CHAIR OR IN THE WHEELCHAIR, THE TENNIS BALL ON THE TABLE: picks up the ball, and turns the hand over completely holding the ball.<br>*Comment : ....*<br>...Side :<br>right ❑ left ❑			0❑<br>1❑<br>2❑<br>3❑
**22.** SEATED ON THE CHAIR OR IN THE WHEELCHAIR, ONE FINGER PLACED IN THE CENTER OF THE DIAGRAM BELOW: raises the finger and places it successively on the 8 squares on the diagram without touching the lines.<br>*Comment : ....*<br>...Side :<br>right ❑ left ❑			0❑<br>1❑<br>2❑<br>3❑

**23.** SEATED ON THE CHAIR OR IN THE WHELLCHAIR, UPPER LIMBS ALONG THE TRUNK: places the two forearms and/or the hands on the table at the same time.<br>*Comment :* ....<br>...		0❑<br>1❑<br>2❑<br>3❑	
**24.** SEATED ON THE CHAIR: stands up without upper limb support and with the feet slightly apart.<br>*Comment :* ....<br>...	0❑<br>1❑<br>2❑<br>3❑		
**25.** STANDING WITH UPPER LIMB SUPPORT ON EQUIPMENT: releases the support and maintains a standing position for 5 seconds with the feet slightly apart, the head, trunk and limbs in midline position.<br>*Comment :* ....<br>...	0❑<br>1❑<br>2❑<br>3❑		
**26.** STANDING WITH UPPER LIMB SUPPORT ON EQUIPMENT: without upper limb support, raises the foot for 10 seconds.<br>*Comment :* ....<br>...Side :<br>right ❑ left ❑	0❑<br>1❑<br>2❑<br>3❑		
**27.** STANDING: without support, touches the floor with one hand and stands up again.<br>*Comment :* ....<br>...	0❑<br>1❑<br>2❑<br>3❑		
**28.** STANDING WITHOUT SUPPORT: takes 10 steps forward on both heels.<br>*Comment :* ....<br>...	0❑<br>1❑<br>2❑<br>3❑		
**29.** STANDING WITHOUT SUPPORT: takes 10 steps forward on a straight line.<br>*Comment :* ....<br>...	0❑<br>1❑<br>2❑<br>3❑		
**30.** STANDING WITHOUT SUPPORT: runs 10 meters.<br>*Comment :* ....<br>...	0❑<br>1❑<br>2❑<br>3❑		
**31.** STANDING ON ONE FOOT WITHOUT SUPPORT: hops 10 times place.<br>*Comment :* ....<br>...Side :<br>right ❑ left ❑	0❑<br>1❑<br>2❑<br>3❑		
**32.** STANDING WITHOUT SUPPORT: without upper limb support, manages to squat and gets up twice in a row.	0❑<br>1❑<br>2❑		

Comment : ....<br>*...*	3❑		
**TOTAL**	D1=	D2=	D3=

SUMMARY OF SCORING:

DIMENSION	CALCULATION OF DIMENSION % SCORES
**D1.** Standing and transfers	Total dimension 1 = _______ x 100 = _ _ ._ _ %<br>13 x 3 39
**D2.** Axial and proximal motor function	Total dimension 2 = _______ x 100 = _ _ ._ _ %<br>12 x 3 36
**D3.** Distal motor function	Total dimension 3 = _______ x 100 = _ _ ._ _ %<br>7 x 3 21
**TOTAL SCORE**	Total mark = _______ x 100 = _ _ ._ _ %<br>32 x 3 96

Patient cooperation : none ❑ medium ❑ optimal ❑

Particularities during this MFM test :

....

....
......

....
...

ACTIVE-SEATED

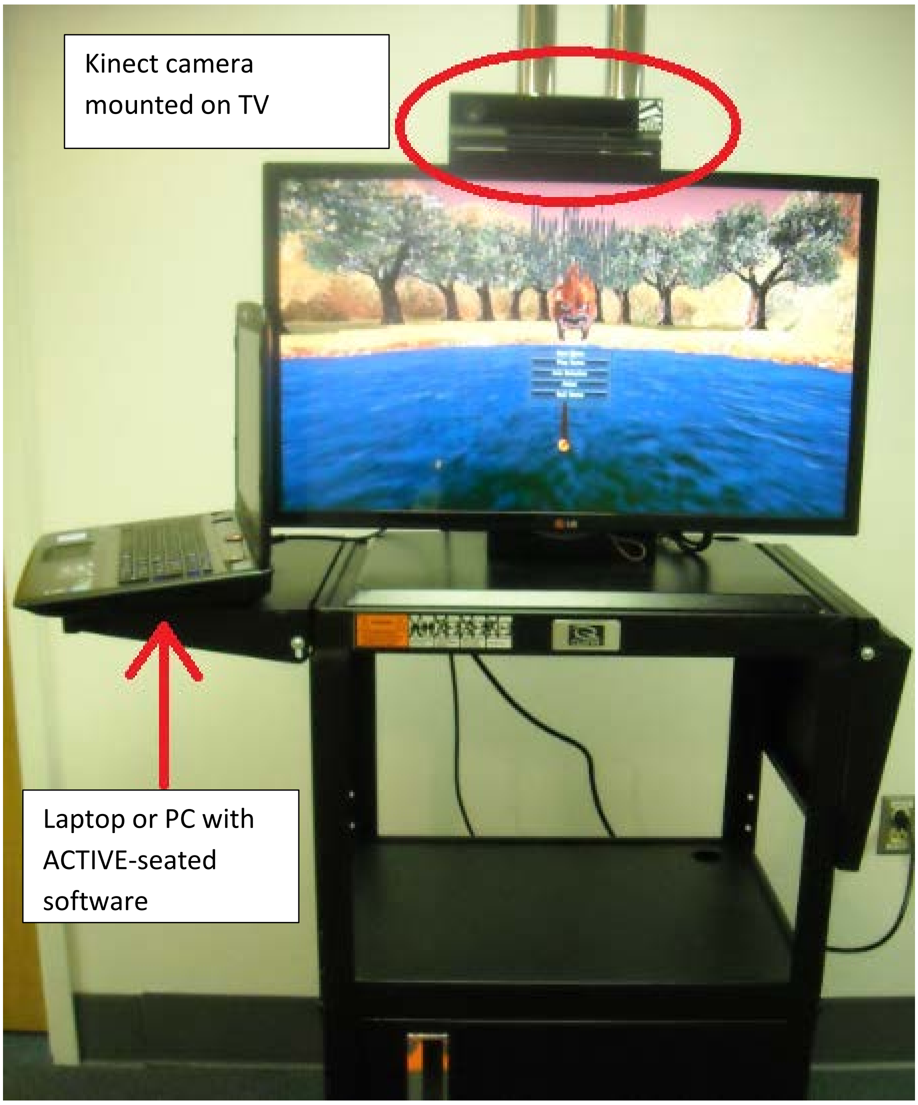

APPENDIX 4. ACTIVITY ASSESSMENT

ACTIMYO

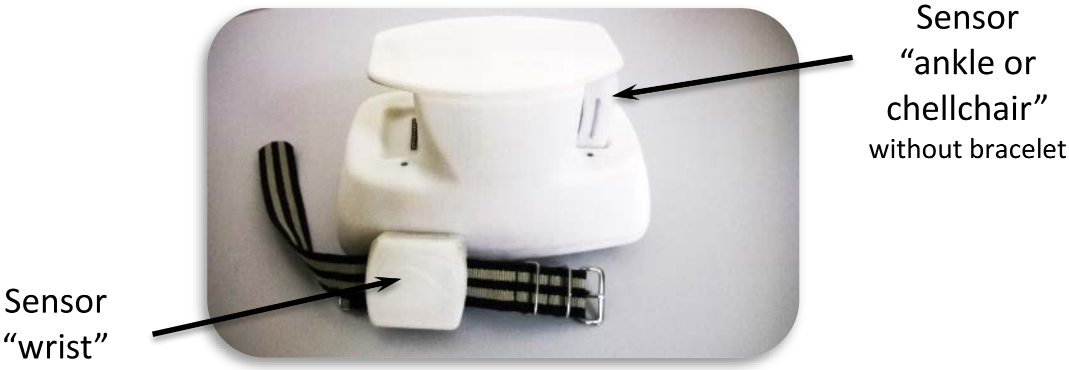

APPENDIX 5. MRI EXAMINATIONS SAFETY SHEET

NMR EXAMINATION SAFETY SHEET
(MUST be filled in before each examination)

DATE: _________

Protocol title: ______________________

Name of NMR examination: _____________________

SECOND NAME: ______________ **First name:** ___________

Date of birth: _______________

Nationality: _________________

If injection of contrast medium:

Batch:
Expiry date:
Volume injected:
Arm injected:

Gender |__| M |__| F ➔ Date of last menstrual period:

Weight |__|__| kg Height |__|__|__| cm Shoe size |__|__|

**CONTRAINDICATIONS TO NMR:**	**1st Interview**		**2nd Interview**	
Cardiac pace maker	YES	NO	YES	NO
Cerebrovascular clips	YES	NO	YES	NO
Intraocular foreign bodies	YES	NO	YES	NO
Ventricular derivation valve	YES	NO	YES	NO
Cardiac valve	YES	NO	YES	NO
Shards of metal	YES	NO	YES	NO
Prostheses (auditory, neurostimulator, etc.)	YES	NO	YES	NO
Spinal osteosynthesis materials	YES	NO	YES	NO
Pregnancy	YES	NO	YES	NO
Claustrophobia	YES	NO	YES	NO
Participation in another trial	YES	NO	YES	NO

If YES: TELL THE RESPONSIBLE DOCTOR AND/OR CANCEL THE EXAMINATION

Surgery:

ONGOING MEDICATION (name and dosage)

-
-

CLINICAL EXAMINATION

BP = / - HR = Plasma glucose:

SIGNATURE OF THE DOCTOR CONDUCTING THE EXAMINATION:

APPENDIX 6. QUALITY OF LIFE

PEDIATRIC QUALITY OF LIFE INVENTORY (PedsQL™) Review Copies :

1) PedsQL™ 4.0 Generic Core Scale

https://eprovide.mapi-trust.org/instruments/pediatric-quality-of-life-inventory/rc_pedsql-4.0-core-all_au4.0_eng-usori

2) PedsQL™ 3.0 Neuromuscular Module

https://eprovide.mapi-trust.org/instruments/pediatric-quality-of-life-inventory/rc_pedsql-3.0-neuromuscular-all_au3.0_eng-usori

All to be used PedsQL forms adapted for ages and administration mode as stated in the protocol text, PedsQL 3.0 Neuromuscular Module (Parent Report for Toddler, Parent Report for Young Child and Teen Self Report) will be provided separately in the Study Manual.

HUI2 – MULTI-ATTRIBUTE HEALTH STATUS CLASSIFICATION SYSTEM

Attribute	Level	Description*
**Sensation**	1	Able to see, hear, and speak normally for age.
	2	Requires equipment to see or hear or speak.
	3	Sees, hears, or speaks with limitations even with equipment.
	4	Blind, deaf, or mute.
**Mobility**	1	Able to walk, bend, lift, jump, and run normally for age.
	2	Walks, bends, lifts, jumps, or runs with some limitations but does not require help.
	3	Requires mechanical equipment (such as canes, crutches, braces, or wheelchair) to walk or get around independently.
	4	Requires the help of another person to walk or get around and requires mechanical equipment as well.
	5	Unable to control or use arms and legs.
**Emotion**	1	Generally happy and free from worry.
	2	Occasionally fretful, angry, irritable, anxious, depressed, or suffering “night terrors”.
	3	Often fretful, angry, irritable, anxious, depressed, or suffering “night terrors”.
	4	Almost always fretful, angry, irritable, anxious, depressed.
	5	Extremely fretful, angry, irritable, anxious, or depressed usually requiring hospitalisation or psychiatric institutional care.
**Cognition**	1	Learns and remembers school work normally for age.
	2	Learns and remembers school work more slowly than classmates as judged by parents and/or teachers.
	3	Learns and remembers very slowly and usually requires special educational assistance.
	4	Unable to learn and remember.
**Self-Care**	1	Eats, bathes, dresses, and uses the toilet normally for age.
	2	Eats, bathes, dresses, or uses the toilet independently with difficulty.
	3	Requires mechanical equipment to eat, bathe, dress, or use the toilet independently.
	4	Requires the help of another person to eat, bathe, dress, or use the toilet.
**Pain**	1	Free of pain and discomfort.
	2	Occasional pain. Discomfort relieved by non-prescription drugs or self-control activity without disruption of normal activities.
	3	Frequent pain. Discomfort relieved by oral medicines with occasional disruption of normal activities.
	4	Frequent pain; frequent disruption of normal activities. Discomfort requires prescription narcotics for relief.
	5	Severe pain. Pain not relieved by drugs and constantly disrupts normal activities.
~~**Fertility**~~	~~1~~	~~Able to have children with a fertile spouse.~~
~~2~~	~~2~~	~~Difficulty in having children with a fertile spouse.~~
	~~3~~	~~Unable to have children with a fertile spouse.~~

Source: Torrance et al. *Medical Care* 1996, Table 1, page 706.
Legend: * - Level descriptions are worded here exactly as presented to respondents of the HUI2 preference survey
Note: Fertility attribute is optional and not part of the standard HUI23-15Q questionnaire

APPENDIX 7. SERIOUS ADVERSE EVENT FORM

INSTITUT DE MYOLOGIE
INNOVER POUR GUERIR

Declaration form for a serious adverse event (SAE) arriving during a biomedical research

This form must be filled up as soon as the event is known and forwarded by

mail at the followed address : **cellule_essai@institut-myologie.org** and by fax at n° **01 44 73 65 83**,

Date of report : |__|__| |__|__| |__|__|__|__|
(dd mm yyyy)

☐ Initial report ☐ Follow-up of SAE previously reported

Title of the study :

Name and address of the centre :

Principal Investigator (name, first name) :

1. Patient information

Name: |__|__|__| Firstname : |__|__| Patient n°: |__|__|__|__|__|__| Date of inclusion : |__|__| |__|__| |__|__|__|__|

Gender : ☐ female
☐ male

Date of birth: |__|__| |__|__| |__|__|__|__|

Age : |__|__| years

Weight: |__|__|__| kg

Height : |__|__|__| cm

2. Serious Adverse Event

☐ Death

☐ Serving life-threatening

☐ Hospitalization or hospitalization exrension

from |__|__| |__|__| |__|__|__|__| to |__|__| |__|__| |__|__|__|__| ☐ *on going*

☐ Inability or invalidity

☐ Other event(s) medically significant

………

………

3. SEVERITY

☐ **1** ☐ 2 ☐ 3 ☐ 4 ☐ 5

Grade	Severity
1	Mild; asymptomatic or mild symptoms; clinical or diagnostic observations only; or intervention not indicated
2	Moderate; minimal, local, or non-invasive intervention indicated; or limiting age-appropriate instrumental activities of daily living [a]
3	Severe or medically significant, but not immediately life-threatening; hospitalization or prolongation of hospitalization indicated; disabling; or limiting self-care activities of daily living [b,c]
4	Life-threatening consequences or urgent intervention indicated [d]
5	Death related to adverse event [d]

Note: NCI CTCAE = National Cancer Institute Common Terminology Criteria for Adverse Events.
Note: Based on the NCI CTCAE (v4.03), which can be found at: http://evs.nci.nih.gov/ftp1/CTCAE/CTCAE_4.03_2010-06-14_QuickReference_8.5x11.pdf

[a] Instrumental activities of daily living refer to preparing meals, shopping for groceries or clothes, using the telephone, managing money, etc.

[b] Examples of self-care activities of daily living include bathing, dressing and undressing, feeding one's self, using the toilet, and taking medications, as performed by patients who are not bedridden.

[c] If an event is assessed as a "significant medical event," it must be reported as a serious adverse event.

[d] Grade 4 and 5 events must be reported as serious adverse events.

4. COMPLETE DESCRIPTION OF THE ADVERSE EVENT (DIAGNOSTIC RETAINED, ANATOMICAL LOCALIZATION, CRITERIA TO CONSIDER THE EVENT AS SERIOUS)

………
………
………
………
………
………
………

Date of arriving : |__|__| |__|__| |__|__|__|__|
(dd mm yyyy)

Hour of arriving: |__|__| |__|__| ☐ ND
(hh min)

→ In case of death

Date of death: |__|__| |__|__| |__|__|__|__|
(dd mm yyyy)

Reason of the death: ……………………………………………………………………

Autopsy : ☐ Yes ☐ No

5. MEDICAL HISTORY AND CONCOMITANT TREATMENT

Relevant medical history: ☐ yes ☐ no

………
………
………

Relevant associated pathology: ☐ yes ☐ no

………
………
………

6. Concomitant treatment before the arriving of the serious adverse event, Except those used to treat the adverse event

☐ yes ☐ no

Commercial Name or DCI	Route	Dose/ 24h	Start Date	On going	End Date	Indication	Causality* (1, 2, 3 or 4)
			|__|__| |__|__| |__|__|__|__|	☐	|__|__| |__|__| |__|__|__|__|		|__|
			|__|__| |__|__| |__|__|__|__|	☐	|__|__| |__|__| |__|__|__|__|		|__|
			|__|__| |__|__| |__|__|__|__|	☐	|__|__| |__|__| |__|__|__|__|		|__|
			|__|__| |__|__| |__|__|__|__|	☐	|__|__| |__|__| |__|__|__|__|		|__|

1= probable 2=possible 3=non related 4=unknown

7. COMPLEMENTARY EXAMINATION PERFORMED ☐ yes ☐ no

Nature or Biological exam performed	Date	Result (please, specify the range for biological analysis)	Clinically significant
	|__|__| |__|__| |__|__|__|__|		☐ yes ☐ no
	|__|__| |__|__| |__|__|__|__|		☐ yes ☐ no
	|__|__| |__|__| |__|__|__|__|		☐ yes ☐ no
	|__|__| |__|__| |__|__|__|__|		☐ yes ☐ no
	|__|__| |__|__| |__|__|__|__|		☐ yes ☐ no

Comments:

....
....
....

8. EVOLUTION

☐ on going ☐ other SAE ☐ resolved with sequel * ☐ resolved without sequel ☐ death ☐ unknown

***please specify** ....

Date of resolution: |__|__| |__|__| |__|__|__|__| ☐ NA
(dd mm yyyy)

Hour of resolution: |__|__| |__|__| ☐ NA
(hh min)

Comments:

....
....
....

9. ACCORDING TO THE INVESTIGATOR, THE SERIOUS ADVERSE EVENT SEEMS TO BE RELATED TO:

☐ Progression of the disease

☐ Procedures of the biomedical research
Which one: ………

☐ Concomitant treatment
Which one: ………

☐ Concomitant disease: ………

☐ Other, please specify: ……

**Name of the reporter:**<br><br>**Phone number:**	**Date and signature:**

PART RESERVED TO THE SPONSOR: DO NOT FILL

Identification number of the event: ………………………..

Date of reception by the sponsor: |__|__| |__|__| |__|__|__|__| N° IDRCB : ………………..

Date of this report: |__|__| |__|__| |__|__|__|__|

☐ Initial ☐ follow-up n° |__| / |__|

According to the sponsor, the serious adverse event seems to be mostly related to:

☐ Progression of the disease

☐ Procedure of the biomedical research

Which one:
……

☐ Concomitant treatment

Which one: ……

☐ Concomitant disease: ……………………………………………………………………………………………………

☐ Other, please specify:……………………………………………………………………………………………………

If, according to the sponsor, the event seems rather related to the investigational treatment:

☐ The adverse event is expected

☐ The adverse event is unexpected

Sponsor’s comments :

……

……

……

……

Name and title of the sponsor representative: ……………………………………………………………… ………………………………………………………………	Date et signature :
