# Supplementary material for: Prospective and longitudinal natural history study of patients with Type 2 and 3 spinal muscular atrophy: Baseline data NatHis-SMA study
Source: PLoS One. 2018 Jul 26;13(7):e0201004. doi: 10.1371/journal.pone.0201004 (PMC6062049; doi:10.1371/journal.pone.0201004)
Supplement: S1 Statistical Analysis Plan — (PDF) [file pone.0201004.s003.pdf]

NatHis-SMA – Statistical Analysis Plan

TRIAL FULL TITLE	Prospective study of the natural history of patients with type 2 and 3 Spinal Muscular Atrophy – NatHis-SMA
ANSM RECORD NUMBER	IDRCB-2014-A01263-44
SAP VERSION	1.0 correspond to the version 0.6 -22/12/2016 without change track + update of the variable list
SAP VERSION DATE	28/03/2018
TRIAL STATISTICIAN	Mélanie Annoussamy
TRIAL COORDINATING INVESTIGATOR	Laurent Servais
SAP AUTHOR	Mélanie Annoussamy

Table of Content

Table of Content 2
1. Abbreviation and Definitions 4
2. Introduction 5
2.1. Preface 5
2.2. Purpose of the analyses 5
3. Study objectives and Endpoints 5
3.1. Study Objectives 5
3.2. Endpoints 5
3.3. Derived variables 7
4. Study Methods 7
4.1. General Study Design and Plan 7
4.2. Inclusion-Exclusion Criteria and General Study Population 7
Inclusion criteria 7
Exclusion Criteria 8
4.3. Study Variables 8
5. Sample Size 10
6. General Considerations 10
6.1. Timing of Analyses 10
6.2. Analysis Populations 11
6.2.1. Full Analysis Population 11
6.2.2. Per Protocol Population 11
6.2.3. First analysis Population 11
6.3. Covariates and Subgroups 11
6.3.1. Covariates 11
6.3.2. Subgroups 11
6.4. First statistical Analyses and Data Monitoring 12
6.4.1. Purpose of the first Analyses 12
6.4.2. Planned Schedule of the first Analysis 12
6.4.3. Scope of Adaptations 12
6.4.4. Stopping Rules 12

6.4.5. Analysis Methods to Minimize Bias 12
6.4.6. Adjustment of Confidence Intervals and p-values 13
6.4.7. Documentation of the first Analyses 13
6.5. Multi-center Studies 13
6.6. Multiple Testing 13
7. Summary of Study Data 13
7.1. Subject Disposition 13
7.2. Protocol Deviations 15
7.3. Demographic and Baseline Variables 15
7.4. Concurrent Illnesses and Medical Conditions 15
7.5. Prior and Concurrent Medications 15
8. Disease Course Analyses 16
8.1. Primary Analysis 16
8.2. Secondary Analyses 16
8.3. Exploratory Analyses 18
9. Safety Analyses 18
9.1. Adverse Events 18
9.2. Deaths, Serious Adverse Events and Other Significant Adverse Events 18
9.3. Pregnancies 18
9.4. Clinical Laboratory Evaluations 18
9.5. Other Safety Measures 18
10. Other Analyses 19
11. Figures 19
12. Reporting Conventions 19
13. Technical Details 19
14. Summary of Changes to the Protocol 19
15. References 19
16. Listing of Tables, Listings, and Figures 20
Listing of Tables, Listings, and Figures 21
17. Appendix 22

1. Abbreviation and Definitions

AE	Adverse Event
CRF	Case Report Form
SAE	Serious Adverse Event
SAP	Statistical Analysis Plan
SMA	Spinal Muscular Atrophy
PFT	Pulmonary Function Test
FVC	Forced vital capacity
PCF	Peak Cough Flow
MEP	Maximum Expiratory pressure
MIP	Maximum Inspiratory pressure
SNIP	Sniff nasal inspiratory pressure
MFM	Motor Function Measure
6MWT	6 Minute Walk Test
MRI	Magnetic Resonance Imagery
PedsQL	Pediatric Quality of Life Inventory™
HUI2	Health Utilities Inc

2. Introduction

2.1. Preface

Several therapeutic strategies for SMA disease treatment are under investigation. However, no effective treatment has currently received market approval. Taking into account the variability of phenotypes in SMA patients, it will be useful to determine which outcome measure(s) will be the most appropriate to assess the efficacy of potential therapies and which biomarkers are predictive of the course of the disease.

The proposed study is a prospective study of the SMA natural history, the purpose of which is to characterize the disease course over 2 years and determine which outcome measures will be the best to assess the efficacy of potential therapies.

2.2. Purpose of the analyses

The study's data analyses are designed primarily to characterize the progression of disease in patients with SMA type 2 and type 3 over a period of approximately 12 to 24 months with the goal of exploring suitable outcome measures for future treatment studies. The time course, the within and between variability, and changes from baseline of the primary, secondary and exploratory endpoints will be analyzed and displayed graphically. Furthermore, multivariate methods such as covariance-structure analysis will be applied to detect dependencies, relations and possible patterns between various outcome measures. Analyses will be performed both overall and by age cohort (2-5 years and 6-30 years) and by ambulant status.

All analyses will be included in the clinical study report.

3. Study objectives and Endpoints

3.1. Study Objectives

Primary objective of this study is to characterize the disease course in SMA type 2 and type 3 patients using standardized evaluations.
Secondary objectives are

- To identify prognostic variables of the disease
- To identify the best outcome measure(s) for future treatment studies
- To identify biomarkers of SMA progression

3.2. Endpoints

The study endpoints will be defined by the age and ambulant status of the patient. The following will be assessed at baseline and changes over time will be recorded:

- o Respiratory function assessment (from interview, medical file and pulmonary function tests):
    - Type of respiratory support (Invasive/Noninvasive)

  - Number of hours off mechanical ventilation per day
  - Number of lower respiratory tract infection per year
  - Pulmonary function tests (PFT) for patients older than 6Y and not completely dependent on mechanical ventilation: forced vital capacity (FVC), peak cough flow (PCF), maximum expiratory pressure (MEP), maximum inspiratory pressure (MIP) and/or sniff nasal inspiratory pressure (SNIP)
- Motor function assessment (from interview, medical file and standardized measurements):
  - Achieved Milestone: ability to hold his head (Yes/No/Never acquired, date achieved, date lost), to sit without support for 10 s, to stand for 10 s, to walk with or without help, to climb stairs, to feed oneself, and others.
  - Standardized assessment (adjusted to age and ambulant status): Evolution of time to get up from floor, to walk/run 10 meters, to climb and descend stairs, the evolution of the distance walked at the 6 MWT, the measured values for Moviplate, and of the % predicted of total reached volume of upper extremity function by ACTIVE-seated
  - Scales: Motor Function Measure (MFM) 20 or 32 depending on the age.
- Activity assessment: Evolution of activity as measured by ActiMyo (in SMA type 2 older than 6 years old, forecasted: 30 patients)
- Muscle strength assessment: Evolution of measured values for Myogrip and Myopinch
- Electrophysiology measurements (EMG)
  - Evolution of the Compound Motor action Potential Amplitude (CMAP)
  - Decrement search
- nuclear magnetic resonance imaging (MRI) (on a subgroup of 20 patients older than 4 years included in Paris, Liège or Strasbourg):
  - Muscle volume changes
  - Intramuscular fatty infiltration progression
  - Indices of disease activity
  - Spinal cord characterization
- Quality of life assessment (from questionnaire and interview):
  - Number of work days missing due to caring for a child with SMA, number of days of school or work missing due to the SMA
  - Family and community events missed due to illness or care
  - PedsQL
  - HUI2
- Orthopedic status (from medical file): Scoliosis, contractures, fractures, use of assistive device
- Biomarker: *SMN* mRNA quantification, SMN protein production, exploratory biomarkers, genotyping (mRNA, DNA and protein profiling)

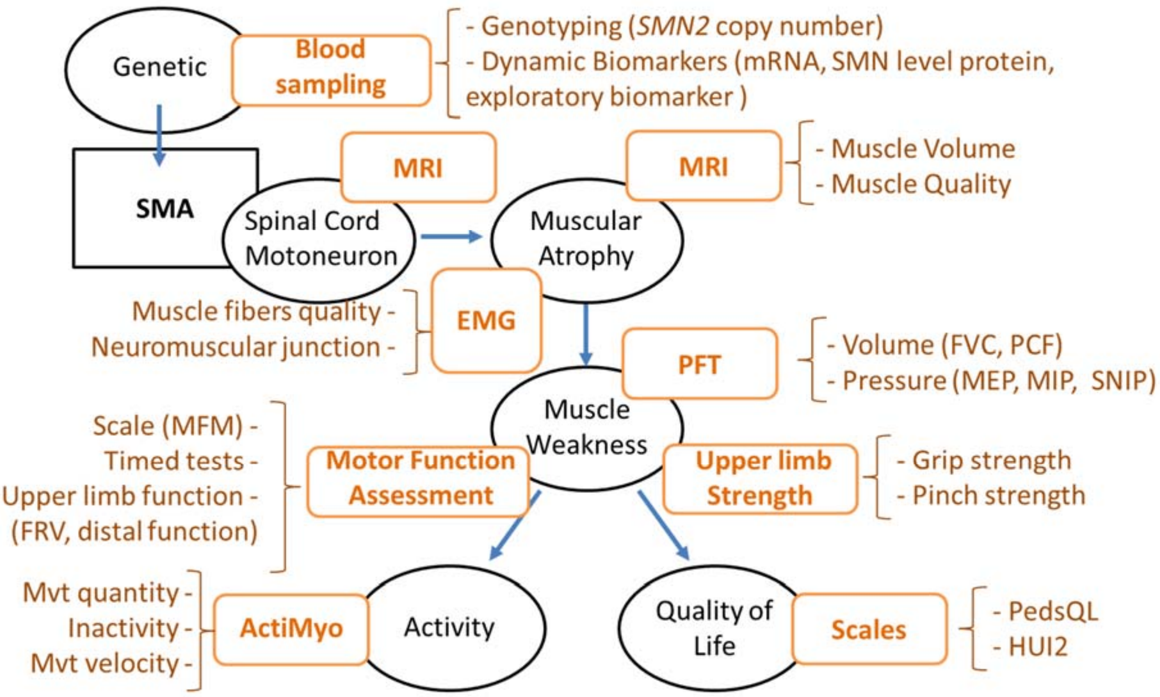

Figure 1 : Endpoints in the SMA context

3.3. Derived variables

Absolute and percent changes over time will be calculated in reference to the baseline value.

4. Study Methods

4.1. General Study Design and Plan

The present study is a prospective, longitudinal and interventional study of pathophysiology.

Initially, the study duration was 2 years (24 months) with a recruitment period of 12 months. The duration of the study have been prolonged by protocol amendment to last 3 years (36 months) in order to have 2 years (24 months) follow up for each patients.

Taking into account the variability of phenotypes and the variability of the age of patients who may participate to this study, we plan to adjust the assessments to patients' age and ambulant status. All patients will be evaluated every 6 months.

4.2. Inclusion-Exclusion Criteria and General Study Population

Inclusion criteria

- Type 2 or 3 spinal muscular atrophy genetically confirmed

- o Age superior or equal to 2 years old up to 30 years of age included
- o For patients older than 6 years old, willing and able to comply with all protocol requirements and procedures.
- o For non-ambulant patients, able to sit upright in a wheelchair for at least three hours
- o Patients over 18 years of age and parent(s)/legal guardian(s) of patients < 18 years of age must provide written informed consent prior to participating in the study and informed assent will be obtained from minors at least 7 years of age when required by regulation.
- o In France only: Affiliated to or a beneficiary of a social security category

Exclusion Criteria

- o Previously treated with an investigational drug within 6 months prior the recruitment in this study.
- o Other condition which may significantly interfere with the assessment of the SMA and is clearly not related to the disease
- o Current or anticipated participation in any therapeutic investigational clinical studies.
- o Patients with specific contraindication to MRI (i.e. metallic foreign body, claustrophobia, and others deemed to be prohibitive by the investigators) will be allowed to participate, but MRI will not be performed.
- o For women : pregnancy or current breastfeeding

4.3. Study Variables

Frequencies and timing of all observations or assessments are described in the following flow chart:

	2 – 5 years old		≥ 6 – 30 years old	
	Inclusion	Follow up visit	Inclusion	Follow up visit
	M0	Every 6 months ± 28 days	M0	Every 6 months ± 28 days
**Verification of selection criteria**	X		X	
**Informed consent**	X		X	
**Demographic data**	X		X	
**Physical examination and vital signs**[1]	X	X	X	X
**SMA natural history/disease progression questionnaire:**<br>- Achieved Milestone<br>- Respiratory function<br>- Number of school and/or work days and family or social event missing due to MTM<br>- Other assessments from the medical file (feeding status, orthopedic status)	X	X	X	X
**Pulmonary function Tests:**<br>- FVC<br>- PCF<br>- MEP<br>- MIP and/or SNIP[2]			X	X
**Timed Test**[3]**:**<br>- Time to rise from floor<br>- Time to walk/run 10 meters<br>- Time to climb and descend stairs<br>- Distance walked in 6MWT			X	X
**Moviplate**			X	X

	2 – 5 years old		≥ 6 – 30 years old	
	**Inclusion**	**Follow up visit**	**Inclusion**	**Follow up visit**
	**M0**	**Every 6 months ± 28 days**	**M0**	**Every 6 months ± 28 days**
**Muscle strength:**<br>- **Myogrip**<br>- **Myopinch**			X	X
**Activity assessment**[4]			X	X
**MFM**	MFM20	MFM20	MFM32	MFM32
**ACTIVE-seated**			X	X
**PedsQL**	X	X	X	X
**HUI2**	X	X	X	X
**MRI**[5]	X	X	X	X
**EMG**<br>- **CMAP**<br>- **Decrement search**	X	X	X	X
**Blood Sample for clinical genotyping**	X		X	
**Blood Sample for biomarker**	X	X	X	X
**Concomitant Treatments**	X	X	X	X
**AE/SAE**		X		X

Time-windows to be used for converting dates into visit numbers are the following:

V1= inclusion visit = M0

V2= FU1 = Follow-up visit #1 = M6 ± 28 days

V3= FU2 = Follow-up visit #2 = M12 ± 28 days

V4 = FU3 = Follow-up visit #3 = M18 ± 28 days

V5 = FU4 = Follow-up visit #4 = M24 ± 28 days

<u>Decision rules that will be used to classify measurements obtained outside of scheduled assessment times:</u>

- Measurements obtained ± 56 days from the visit will be classified into the visit.
- Measurements obtained outside a delay of ± 56 days from a visit will not be part of statistical analyses.
- Genotyping: If not performed at baseline, can be performed at M6
- If NMR unavailable at baseline, NRM assessment will be performed at M6 and then every year in order to have 1 year between 2 assessments for each selected patients

<u>Methods for handling multiple measurements that occur within the same assessment time window:</u>
Patients with SMA are particularly fatigable. It is possible that some evaluations will be invalidated and performed once again after a sufficient rest period. In that case, only the validated data will be entered into the database and statistically analyzed.

Regarding Myotools and PFT evaluations, on average, 3 tests are performed. The value retained for analysis will be the maximum one.

The list of all variables with precise description (type, range, …)

See table1 at the end of this document

Derived variables:

Absolute and percent changes over time will be calculated in reference to the baseline value.

Missing data:

Missing data will generally not be part of the analysis, except for some variables as height or ulna length of adult patients for which the value at the previous visit will be considered.

Reason for missing data should be available and could be an additional information per se.

Methods for combining multiple variables into a single value (i.e. PedsQL and HUI2): not appropriate. Scores will be computed and monitored before entered into the database

5. Sample Size

Since SMA is a rare disease, the participation to the trial will be proposed to all patients affected by SMA type 2 and type 3 and who fulfill the inclusion criteria in 6 centers in France (Paris, Toulouse, Lille, Strasbourg, Nantes and Lyon), 2 centers in Belgium (Liège and Leuven) and 1 center in Germany (Essen).

A recent one-year natural history study in non-ambulant patients has demonstrated that significant loss strength can be demonstrated after 14 years in a limited number of patients (n=7) (A.M. Seferian, 2015). However, given the heterogeneity of the disease and the large age range planned in the inclusion criteria, we want to gather at least one year follow up data in 50 eligible patients. From our previous experience in natural history studies, we anticipate a 25% drop out of the study; we thus want to include at least 70 patients.

6. General Considerations

6.1. Timing of Analyses

Initially, there were two analyses planned for this study:

September 2016: A first analysis will be performed on the 81 baseline data and after 40 evaluable patients have completed their 6 months follow up visit by June 2016.

November 2017: A final analysis of data set will be performed of at least 50 evaluable patients after database lock (in September 2017)

With the prolongation of the study by protocol amendment, we decide to perform 3 analyses: the first one on the baseline data (February 2017), the second one on the 1year follow up data (November 2017) and the third one at the end of the study (November 2018).

6.2. Analysis Populations

6.2.1. Full Analysis Population

All subjects who have be enrolled (i.e. who has signed an informed consent) and performed the baseline visit. We expect to include 81 patients.

6.2.2. Per Protocol Population

All subjects who have completed the study (i.e. who has performed a minimum of 3 visits) and who have not substantially deviated from the protocol (protocol deviation is defined in §7.2). As evaluations have been adjusted according to the age and to the ambulant status of the patient, the per protocol population may differ from a variable to another.

6.2.3. First analysis Population

The first analysis population will be constituted by the 81 expected patients who performed the baseline visit and the first 40 subjects who will have performed at least baseline and 6 months follow-up visits with no major protocol deviations (as defined in § 7.2).

6.3. Covariates and Subgroups

6.3.1. Covariates

As SMA is a rare disease, the present study has been designed as a multicenter study with 9 centers and around 8 subjects per centers. In order to consider all centers as a whole, a special attention has been made for the training of evaluators.

Center effect will be tested on each endpoint or centers will be taken into account as a covariate in statistical analyses.

The type of SMA (II or III), the ambulant status (ambulant or non-ambulant) and the range of age (2-5, 6-10; 11-15, 16-20, 21-25, 26-30) will obviously determine the results of evaluations and will be considered as covariates each time it is possible. « Age » may be included as a continuous covariate for certain analysis.

Gender may also influence results of evaluations and will be considered as covariate.

6.3.2. Subgroups

Analyses will be performed in the whole population but also by SMA type (II or III), ambulant status (ambulant or non-ambulant) and by age range (2-5, 6-10; 11-15, 16-20, 21-25, 26-30 or 2-5 and 6-30 according to the patients repartition and sample size in each category)

6.4. First statistical Analyses and Data Monitoring

6.4.1. Purpose of the first Analyses

A first statistical analysis will be realized on data obtained from the baseline of the 81 patients enrolled in the study and on the 40 first patients having performed the baseline and the 6 months visits. The aim of this first analysis is to characterize the study population end to get first data on variability of respiratory assessments. It will be mainly descriptive.

6.4.2. Planned Schedule of the first Analysis

The first analysis is planned in December 2016, after data of the 40 first patients having performed the baseline and the 6 months visits will be monitored, double entered and validated in the database. Data includes NMR, Actimetry and biological data.

6.4.3. Scope of Adaptations

No adaptation post analysis is forecasted. But if any, the decision will be taken by the Join Steering Committee.

The first data analysis may be performed on more than 40 patients on JSC decision if there are more than 40 baseline + data available data.

6.4.4. Stopping Rules

Not appropriate

6.4.5. Analysis Methods to Minimize Bias

Selection bias: Selection criteria have been define to enroll common SMA patients and avoid any patients with important comorbidities or specific/untypical features that makes then derive from the general SMA population.

Enrollment bias: Patients will be enrolled in 9 centers specialized in neuromuscular diseases with common standard of care.

Standardized assessments bias: A great effort has been done on physiotherapists (PT) training in order to maximize assessments standardization and minimize evaluator effect. Each PT has been trained theoretically and practically. Moreover refresh training sessions have been organized in situ for every PT.

The same clinical research associate (CRA) has performed all site initiation visits, and has trained all participating site staff to protocol and study procedures to minimizing center effect.

Quality of all collected data has been verified by regular monitoring visits in all investigational sites.

Control quality of data entered into the database is assured by performing a double data entry.

The first analysis will be performed by an AIM biostatistician in collaboration with a Roche Biostatistician.

Data and analyses results will be shared with the join steering committee of the Study, the project managers of AIM and Roche and all scientific responsible as defined in the project plan of the study.

Consequences on the study will be shared with investigators shortly after decision.

6.4.6. Adjustment of Confidence Intervals and p-values

The first analysis will be mainly descriptive. P-values and the type-I error α will not be adjusted. All p-values will be considered as purely exploratory.

6.4.7. Documentation of the first Analyses

The first analysis will lead in a first analysis report which will be preserved. Data used for the first analysis will be exported in an Excel file and conserved. All files from the statistical software (SPSS 22) will also be preserved.

6.5. Multi-center Studies

As explained in section 6.3.1, due to the rarity of the disease, the study has been designed as a multicenter study with 9 centers and around 8 subjects per centers. In order to consider all centers as a whole, a special attention has been made for the training of evaluators.

Center effect will be tested on each endpoint or centers will be take into account as a covariate in statistical analyses.

6.6. Multiple Testing

The main objectives of NatHis-SMA study will be assessed by multiple primary variables. Each variable will be explored independently and by subgroups (SMA type, Age range and ambulant status)

7. Summary of Study Data

The number and percentage of enrolled patients, of patients who completed the study and of patients who discontinued as well as reasons for early discontinuation will be presented.

All continuous variables will be summarized using the following descriptive statistics: n (non-missing sample size), mean, standard deviation, median, maximum and minimum. The count, frequency and percentages (based on the non-missing sample size) of observed levels will be reported for all categorical measures. All data will be listed globally, sorted by site, subject, ambulant status, age range and SMA type (and by visit number within subjects).

7.1. Subject Disposition

Every patient will be evaluated at baseline (V1-inclusion) and then every 6 months (±28 days) during 2 years. All patients will have 5 visits.

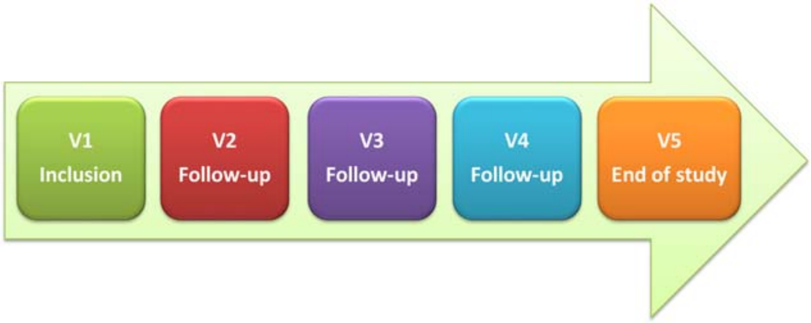

Figure 2 : Study schedule

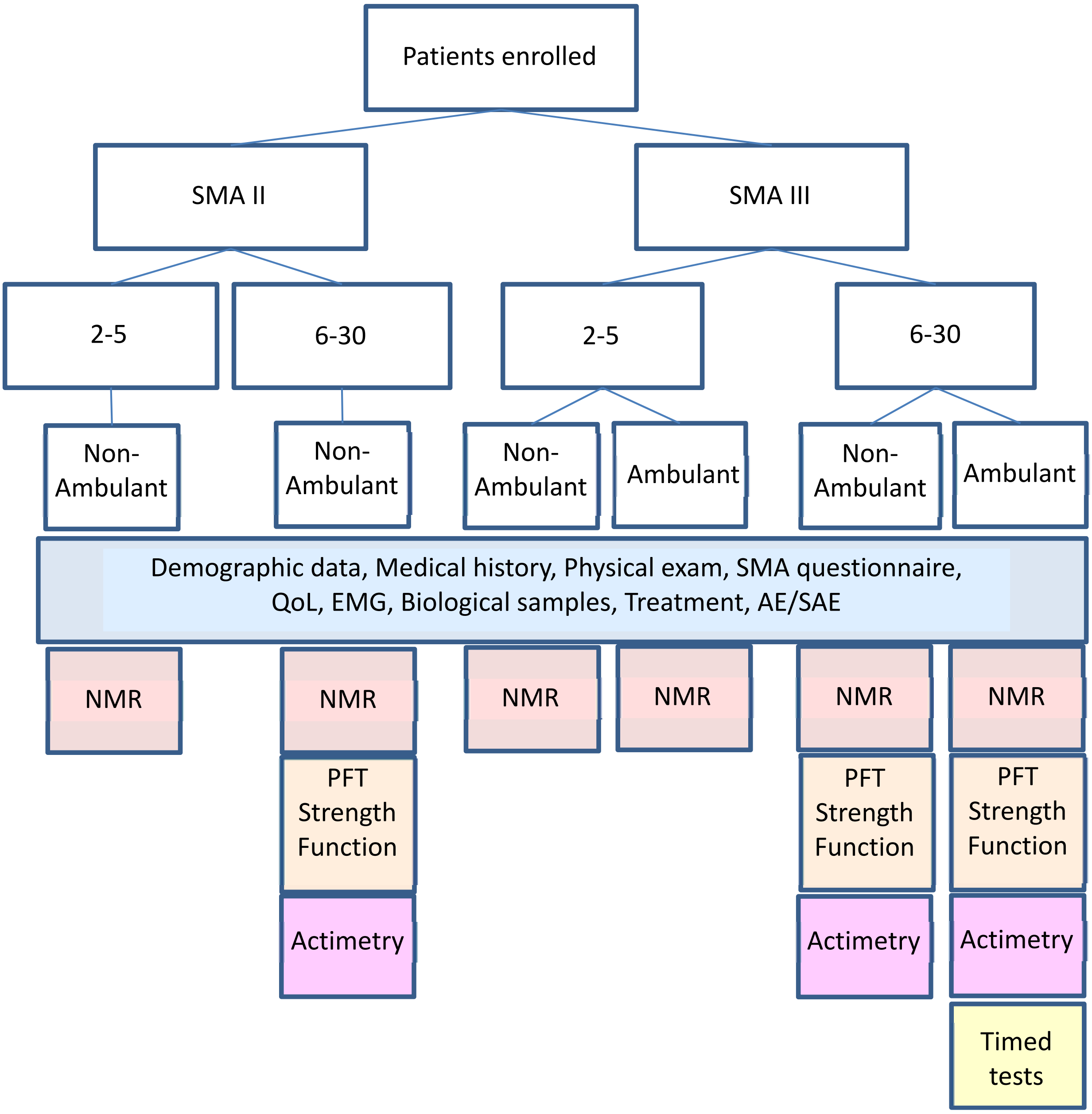

PFT performed in patients older than 6 years old
Timed tests performed in ambulant patients older than 6 years old
NMR performed in 20 subjects older than 4 years old
Actimetry performed in 30 non-ambulant patients (SMA II or SMA III)

Figure 3 : Populations for each evaluation category

A patient will be considered as having performed a study visit as soon as he has performed the physical exam i.e. if vital signs and physical exam data are completed.

Premature study discontinuation date and reasons will be collected in the End of Study section of the CRF.

The summary statistics will be produced in accordance with section 7

7.2. Protocol Deviations

Major deviations will impact the statistical analyses mainly by reducing the sample size.

Major deviations are defined as protocol modifications affecting assessments and data integrity (mistake(s) in assessment performed or assessment performed with technical or compliance issue, visit delayed out of protocol tolerance…)

Subjects performing only the baseline visit will be removed from the statistical analyses of changes over time.

7.3. Demographic and Baseline Variables

All data obtained at the first visit (inclusion visit) will be considered as a baseline data.

For data reported from medical file, it is allowed to report data until 1 year before the inclusion (Except medical and surgical history & previous hospitalization in relation with SMA, prenatal period, diagnostic data + SMA questionnaire data).

The summary statistics will be produced in accordance with section 7.

7.4. Concurrent Illnesses and Medical Conditions

It is planned to code medical conditions by using the MedDRa dictionary.

The summary statistics will be produced in accordance with section 7.

7.5. Prior and Concurrent Medications

Medical indications will be coded by the MedDRa dictionary. We will use the International Nonproprietary Name (INN) for prior and concurrent medication.

The summary statistics will be produced in accordance with section 7.

8. Disease Course Analyses

The primary objective of this study is to assess changes in disease course over a period of time of 12 and, when possible, 24 months and to describe the natural history of patients with type II and III SMA. These analyses will be mainly descriptive.

All variable of interest will be sorted by subject (within study center). Data will be summarized by visit. N, mean, Standard deviation, Maximum and minimum will summarize continuous variables, whereas counts and percentages will summarize categorical variables.

To assess a potential center effect, we will compare quantitative outcomes of interest using the non-parametric Kruskal-Wallis signed-ranks test due to samples size lower than 30. Post hoc tests based on Dunn-Bonferroni method will be added in case of significance. Potential effects of age range, SMA type and ambulant status type will be assessed by the student t-test or the non-parametric Kruskal-Wallis signed-ranks test for sample sizes lower than 30.

For assessments performed on both side of patients (grip and pinch strength, and MoviPlate score), in order to assess a potential side effect, we will compare the maximum value obtained between the dominant and non-dominant hand using the Student t-test or the non-parametric Kruskal-Wallis signed-ranks test for sample size lower than 30.

All analyses will be performed using the IBM SPSS 22 statistics software. The limit of statistical significance (p-value) will be set to 0.05.

8.1. Primary Analysis

Changes in outcome(s) of interest over the time will be displayed graphically and analyzed using repeated measures ANOVA or Friedman two-way analysis of variance for sample size lower than 30 and for qualitative variables. Adjustment with covariates (study center, age range, SMA type, ambulant status) may be performed each time it is allowed. For positive result in ordered and categorical variables, Generalized Linear Model (GLM) and particularly Generalized Estimating Equations (GEE) could be used to study the amount of change, or trends over time.

To account for dependence between values at different time points, a model of evolution of each outcome of interest will be constructed by applying generalized linear models to the data. Estimations of changes will be obtained from these models with 'outcome of interest' as the dependent variable and 'time' as an independent variable. ("Time" will be treated as ordered categorical variable as evaluations are performed every 6 months).

8.2. Secondary Analyses

One of the secondary objectives of the study is **to identify the best outcome measure(s) for future treatment studies**. It will be assessed by studying the internal responsiveness, which characterizes the ability of a measure to change over a particular pre-specified time frame. It will be determined from the primary analysis. The effect size statistics standardized response mean (SRM) which should provide

an information on the magnitude of change in the measure may also be studied. (SRM may be preferred over the paired t-test because it removes the dependence on sample size. SRM provide an estimate of change in the measure, standardized relative to the between patient variability (*variability of the difference*)). (1)

It will be the same for **identifying biomarkers of SMA progression**.

Another secondary objective of the study is **to identify Prognostic variables of the disease**.

Reasons for studying prognostic factors are:

- To learn the relative importance of several variables that might affect or be associated with disease outcome
- To improve the design of further clinical trials

Prognostic factor analysis will attempt to assess the relative importance of several predictor variables simultaneously.

First of all we will study the responsiveness of outcome of interest. Internal responsiveness will be determined by the primary analysis.

External responsiveness, which reflects the extent to which changes in a measure over a specified time frame relate to corresponding changes in a reference measure of health status, will be assessed by studying relationship between outcomes of interest and the MFM score (external indicator of change) (assumed to be the gold standard in the measurement of the changes in SMA patients (2)).

We will verify if there is an association between quantitative variables and MFM scores by assessing the Spearman's ρ and the Pearson's *r*. Both coefficients will be calculated in order to determine if the correlation is rather monotonic than linear (S>P). Nevertheless, for very small samples which probably do not met the assumption of Pearson (Constant variance and linearity), and for categorical variables, we will consider only the Spearman's rho. We may add partial correlation analyses, each time it will be possible.

The prognostic value of variables of interest will be then determined by a multiple regression model (Cox Model). The event will be a reduction of 2 points at the MFM score.

Nevertheless, as there are only four possible time points, there will be a huge number of ties which may influence the estimators by Cox's model. Moreover, the Cox model assumes right-censored data. Here, we only know that the event falls into the interval 0-6m, 6m-12m, 12m-18m etc. I.e. it as interval-censored data. Therefore with this violation of the assumptions, the Cox Model may be not appropriate.

Another possible approach proposed by Paul Jordan is the following:

The patients can be categorized into 5 classes:

Class	Occurrence of event
I	Never (24 months observed)
II	18-24 m

III	12-18 m
IV	6 – 12 m
V	0 – 6m

This classes are strictly ordered: I is the best outcome and V the worst. With these classes one could try to fit proportional-odds model :

$$logit[P(Y \leq j)] = \alpha_j \, \beta_x \;\; ; j = I, \dots, V$$

where β_x is a linear predictor (covariates).

However, this model is only applicable if all subjects have the same observation period. I.e. it cannot deal with censored data. If there are many patients who leave the study - say - after the 12m visit, one can consider to include only classes I-III. Nevertheless the proportional-odds model may be used to for a sensitivity analysis: it would be interesting to know whether the same covariates as in the Cox-model have a prognostic value for disease progression.

8.3. Exploratory Analyses

To define

9. Safety Analyses

9.1. Adverse Events

Adverse events may be considered as a part of the natural history of the disease, especially the respiratory infections. Nevertheless, the summary statistics will be produced in accordance with section 7.

When calculating the incidence of adverse events, or any sub classification by AE attribute (time period, severity…, MedDRA dictionary will be used to code AE occurrence. Subjects who experienced a given AE will be counted. Repetition of a given AE per patient will also be counted. The denominator will be the total of the sample size

9.2. Deaths, Serious Adverse Events and Other Significant Adverse Events

Not attended but if any, the summary statistics will be produced in accordance with section 7.

9.3. Pregnancies

Not attended but if any, the summary statistics will be produced in accordance with section 7.

9.4. Clinical Laboratory Evaluations

Not appropriate. There is no clinical laboratory evaluation performed for a safety reason.

9.5. Other Safety Measures

Not appropriate

10. Other Analyses

Not scheduled

11. Figures

Descriptive statistics will be illustrated by repartition histograms for qualitative variables and scatter plots for quantitative one.

12. Reporting Conventions

P-values ≥ 0.001 will be reported to 3 decimal places; p-values less than 0.001 will be reported as <0.001. The mean, standard deviation, and any other statistics other than quantiles, will be reported to one decimal place greater than the original data. Quantiles, such as median, or minimum and maximum will use the same number of decimal places as the original data. Estimated parameters as regression coefficients will be reported with 3 decimals.

13. Technical Details

Not applicable

14. Summary of Changes to the Protocol

Modifications in the protocol which may modify the statistical analyses:

- Modifications of the Active Seated software
- Spine characterization by NMR
- Modification of the study follow-up (2 years follow up per patients *ie* 4 follow-up visits for each patient)

15. References

1. Husted JA, Cook RJ, Farewell VT, Gladman DD. Methods for assessing responsiveness: a critical review and recommendations. J Clin Epidemiol. 2000;53(5):459-68.
2. Dessaud E, Andre C, Scherrer B, Berna P, Pruss R, Cuvier V, et al. Results of a phase II study to assess safety and efficacy of olesoxime (TRO19622) in 3-25 years old spinal muscular atrophy patients. Neuromuscular Disord. 2014;24(9-10):920-1.

16. Listing of Tables, Listings, and Figures

Will be later defined

Table title	Number	Population	Endpoint	Time Points or how to conglomerate	Covariates or subgroups	Summary statistics	Formal analysis	Foot notes
Summary of Baseline Variables	1	Full Analysis		Inclusion visit (V1)	SMA type<br>Age range<br>Ambulant status	N, mean, SD, Median, min, max		
	2							
	3							
	4							

17. Appendix

Table 1 : List of variables

Label	Unit	Type	value	summary of data
**Description of the population (at baseline)**	Descriptive statistics on the whole population and by SMA type + comparison between SMA type.			
SMA type		categorical	2 or 3	Percent
Age	months	quantitative	2 to 30	Mean, SD, Median, Min, Max
Age range		categorical	(2-5) or (6-30)	Percent
Gender		categorical	1 (M) or 2 (F)	Percent
Hereditary		categorical	0 (No) or 1 (Yes)	Percent
Medical and surgical history		categorical	0 (No) or 1 (Yes)	Percent
Eye/Ear/Nose/throat history		categorical	0 (No) or 1 (Yes)	Percent
Neurologic and psychiatric history		categorical	0 (No) or 1 (Yes)	Percent
Pulmonary history		categorical	0 (No) or 1 (Yes)	Percent
Cardiovascular and blood history		categorical	0 (No) or 1 (Yes)	Percent
Musculoskeletal history		categorical	0 (No) or 1 (Yes)	Percent
Hepatic-gastrointestinal history		categorical	0 (No) or 1 (Yes)	Percent
Metabolic history		categorical	0 (No) or 1 (Yes)	Percent
Genitourinary history		categorical	0 (No) or 1 (Yes)	Percent
Skin history		categorical	0 (No) or 1 (Yes)	Percent
Previous hospitalization due to SMA		categorical	0 (No) or 1 (Yes)	Percent
Selection criteria	Will not be analyzed			
**Physical Examination**	Descriptive statistics at baseline and changes over the study period + comparison between SMA type. On the whole population and by SMA type			
Ambulant status		categorical	0 (Non ambulant) or 1 (Ambulant)	Percent
Vital signs (heart rate, respiratory rate, systoloic and diastolic blood pressure)		quantitative		Mean, Median, Min, Max
Eye/Ear/Nose/throat		categorical	0 (Normal), Abnormal (1), Unknown (2)	Percent
Neurologic and psychiatric		categorical	0 (Normal), Abnormal (1), Unknown (2)	Percent
Pulmonary		categorical	0 (Normal), Abnormal (1), Unknown (2)	Percent
Cardiovascular and blood		categorical	0 (Normal), Abnormal (1), Unknown (2)	Percent
Musculoskeletal		categorical	0 (Normal), Abnormal (1), Unknown (2)	Percent
Hepatic-gastrointestinal		categorical	0 (Normal), Abnormal (1), Unknown (2)	Percent
Metabolic		categorical	0 (Normal), Abnormal (1), Unknown (2)	Percent
Genitourinary		categorical	0 (Normal), Abnormal (1), Unknown (2)	Percent
Skin		categorical	0 (Normal), Abnormal (1), Unknown (2)	Percent
Contracture		categorical	0 (No) or 1 (Yes)	Percent

Label	Unit	Type	value	summary of data
Brooke score	#	categorical	1 to 6	Percent
Physiotherapy	#	categorical	0 (No) or 1 (Yes)	Percent
Frequence physiotherapy	nb/wk	quantitative	0 to 7	Mean, Median, Min, Max
**SMA Questionnaire**	Descriptive statistics at baseline and changes over the study period. On the whole population and by SMA type			
Work days missing due to SMA child		quantitative	0 to	Mean, Median, Min, Max
School or Work days missing due to SMA		quantitative		Mean, Median, Min, Max
Events missed due to SMA		quantitative		Mean, Median, Min, Max
Milestone Acquisition		categorical	0 (No) or 1 (Yes)	Percent
Age of acquisition		quantitative		Mean, Median, Min, Max
Milestone Loss		categorical	0 (No) or 1 (Yes)	Percent
Age of loss		quantitative		Mean, Median, Min, Max
Mental retardation		categorical	0 (No) or 1 (Yes)	Percent
IQ total formaly evaluated	#	categorical	0 (No) or 1 (Yes)	Percent
IQ Age of evaluation	months	quantitative		Mean, Median, Min, Max
IQ value	#	quantitative		Mean, Median, Min, Max
School attendance (for children)		categorical	0 (No) or 1 (Yes)	Percent
Which level (for children)		categorical	0 (Maternelle) to 12 (Terminale)	Percent
Employment status (for adults)		categorical	1 (full time) to 4 (unemployed)	Percent
Educational status (for adults)		categorical	1 (elementary school) to 4 (post graduate)	Percent
Care level (for teenager and adults)		categorical	1 (self carer) or 2 (caregiver required)	Percent
Breathing support		categorical	0 (No) or 1 (Yes)	Percent
Number of hour of ventilation per day	#	quantitative		Mean, Median, Min, Max
Respiratory lower tract infection		categorical	0 (No) or 1 (Yes)	Percent
Mean number per year	#	quantitative		Mean, Median, Min, Max
Sleep apnea		categorical	0 (No) or 1 (Yes)	Percent
Vital capacity assessed		categorical	0 (No) or 1 (Yes)	Percent
Date of last assessment				
FVC value	L	quantitative	0 to 100	Mean, Median, Min, Max
Difficulty for feeding		categorical	0 (No) or 1 (Yes)	Percent
Date of last assessment				
Current need of a feeding tube		categorical	0 (No) or 1 (Yes)	Percent
Past need of a feeding tube		categorical	0 (No) or 1 (Yes)	Percent
Scoliosis		categorical	0 (No) or 1 (Yes)	Percent
Arthrodesis		categorical	0 (No) or 1 (Yes)	Percent
Date of arthrodesis changes into Age		quantitative		Mean, Median, Min, Max
Scoliosis angle		categorical	1 (<30°), 2 (30-45°) or 3 (>45°)	Percent

Label	Unit	Type	value	summary of data
Fracture		categorical	0 (No) or 1 (Yes)	Percent
Assistive device		categorical	0 (No) or 1 (Yes)	Percent
Quality of life assessments	Descriptive statistics at baseline and changes over the study period + comparison between SMA type. On the whole population and by SMA type			
PedsQL Generic Core scale Completed form		categorical	1 (2-4yo), 2 (5-7 yo) or 3 (young adult)	Percent
Score Total scale	#	quantitative		Mean, Median, Min, Max
Score Physical health summary	#	quantitative		Mean, Median, Min, Max
Psychosocial health summary	#	quantitative		Mean, Median, Min, Max
PedsQL Neuromuscular module Completed form		categorical	1 (2-4yo), 2 (5-7 yo) or 3 (adolescent child)	Percent
Neuromuscular disease I	#	quantitative		Mean, Median, Min, Max
Communication II	#	quantitative		Mean, Median, Min, Max
Family ressources III	#	quantitative		Mean, Median, Min, Max
PedsQL Total score	#	quantitative		Mean, Median, Min, Max
HUI2 overall Score	#	quantitative	-0,03 to 1 where Dead has a utility of 0 and healthy has a utility of 1	Mean, Median, Min, Max
HUI2 Sensation	#	quantitative		Mean, Median, Min, Max
HUI2 Mobility	#	quantitative		Mean, Median, Min, Max
HUI2 Cognition	#	quantitative		Mean, Median, Min, Max
HUI2 SelfCare	#	quantitative		Mean, Median, Min, Max
HUI2 Emotion	#	quantitative		Mean, Median, Min, Max
HUI2 Pain	#	quantitative		Mean, Median, Min, Max
HUI3 Overall score	#	quantitative		Mean, Median, Min, Max
HUI3 Vision	#	quantitative		Mean, Median, Min, Max
HUI3 Hearing	#	quantitative		Mean, Median, Min, Max
HUI3 Speech	#	quantitative		Mean, Median, Min, Max
HUI3 Emotion	#	quantitative		Mean, Median, Min, Max
HUI3 Pain	#	quantitative		Mean, Median, Min, Max
HUI3 Ambulation	#	quantitative		Mean, Median, Min, Max
HUI3 Dexterity	#	quantitative		Mean, Median, Min, Max
HUI3 Cognition	#	quantitative		Mean, Median, Min, Max
Electrophysiology measurements	Descriptive statistics at baseline and changes over the study period + comparison between SMA type. On the whole population and by SMA type			
CMAP - Accessory-trapezius	mV	quantitative		Mean, Median, Min, Max
CMAP - Radial-anconeus	mV	quantitative		Mean, Median, Min, Max
CMAP - Ulnar-ADM	mV	quantitative		Mean, Median, Min, Max
CMAP - Peroneal-tibialis anterior	mV	quantitative		Mean, Median, Min, Max
5th/1st CMAP - Accessory-trapezius	%	quantitative		Mean, Median, Min, Max

Label	Unit	Type	value	summary of data
5th/1st CMAP - Radial-anconeus	%	quantitative		Mean, Median, Min, Max
5th/1st CMAP - Ulnar-ADM	%	quantitative		Mean, Median, Min, Max
5th/1st CMAP - Peroneal-tibialis anterior	%	quantitative		Mean, Median, Min, Max
CMAP tot area - Accessory-trapezius	mV.ms	quantitative		Mean, Median, Min, Max
CMAP tot area - Radial-anconeus	mV.ms	quantitative		Mean, Median, Min, Max
CMAP tot area - Ulnar-ADM	mV.ms	quantitative		Mean, Median, Min, Max
CMAP tot area - Peroneal-tibialis anterior	mV.ms	quantitative		Mean, Median, Min, Max
5th/1st CMAP area ratio - Accessory-trapezius	%	quantitative		Mean, Median, Min, Max
5th/1st CMAP area ratio - Radial-anconeus	%	quantitative		Mean, Median, Min, Max
5th/1st CMAP area ratio - Ulnar-ADM	%	quantitative		Mean, Median, Min, Max
5th/1st CMAP area ratio - Peroneal-tibialis anterior	%	quantitative		Mean, Median, Min, Max
Pulmonary function tests	Descriptive statistics at baseline and changes over the study period + comparison between SMA type. On the whole subgroup 6-30 yo and by SMA type			
FVC	L	quantitative		Mean, Median, Min, Max
% of predicted FVC	%	quantitative		Mean, Median, Min, Max
PCF	L/sec	quantitative		Mean, Median, Min, Max
MEP	cmH2O	quantitative		Mean, Median, Min, Max
MIP	cmH2O	quantitative		Mean, Median, Min, Max
SNIP	cmH2O	quantitative		Mean, Median, Min, Max
Timed Tests	Descriptive statistics at baseline and changes over the study period on the whole subgroup 6-30 yo + ambulant (ie SMA3)			
6MWT	m	quantitative		Mean, Median, Min, Max
10 meters timed walk/run	sec	quantitative		Mean, Median, Min, Max
Time to rise from floor test	sec	quantitative		Mean, Median, Min, Max
Time for climbing stairs	sec	quantitative		Mean, Median, Min, Max
Time for descending stairs	sec	quantitative		Mean, Median, Min, Max
Strength Assessments	Descriptive statistics at baseline and changes over the study period + comparison between SMA type. On the whole subgroup 6-30 yo and by SMA type			
Maximal Grip strength	kg	quantitative	0 to 90 kg (resolution of 0,01 kg)	Mean, Median, Min, Max
Maximal Pinch strength	kg	quantitative	0 to 10 kg (resolution of 0,001 kg)	Mean, Median, Min, Max
Function assessment	Descriptive statistics at baseline and changes over the study period + comparison between SMA type. On the whole subgroup 6-30 yo and by SMA type			
Score MoviPlate	#	quantitative		Mean, Median, Min, Max
Functional Reaching volume (FVR)	m3	quantitative		Mean, Median, Min, Max
Percent predicted FRV	%	quantitative		Mean, Median, Min, Max
velocity of movements	m/sec	quantitative		Mean, Median, Min, Max
acceleration of movement	m/sec²	quantitative		Mean, Median, Min, Max
movement patterns	2D graphical representation			

Label	Unit	Type	value	summary of data
MFM score total	#	quantitative		Mean, Median, Min, Max
MFM score D1	#	quantitative		Mean, Median, Min, Max
MFM score D2	#	quantitative		Mean, Median, Min, Max
MFM score D3	#	quantitative		Mean, Median, Min, Max
ActiMyo	Descriptive statistics at baseline and changes over the study period on a subgroup of 30 patients 6-30 yo + non-ambulant (ie SMA2)			
Norm of angular velocity of the wrist	d°/sec	quantitative		Mean, Median, Min, Max
Temporal derivate of elevation angle of forearm	d°/sec	quantitative		Mean, Median, Min, Max
Quantity of movements	#	quantitative		Mean, Median, Min, Max
Mean of movement duration	msec	quantitative		Mean, Median, Min, Max
time of inactivity during the day	min	quantitative		Mean, Median, Min, Max
Muscle and Spine cord NMR	Descriptive statistics at baseline and changes over the study period + comparison between SMA type. On a subgroup of 20 patients older than 4 yo and by SMA type			
Triceps Cross-Section	cm²	quantitative		Mean, Median, Min, Max
Triceps Muscle Tissue Cross-Section	cm²	quantitative		Mean, Median, Min, Max
Triceps Muscle Volume	cm3	quantitative		Mean, Median, Min, Max
Triceps Fat Percentage	%	quantitative		Mean, Median, Min, Max
Triceps T2 mean	msec	quantitative		Mean, Median, Min, Max
Triceps Percentage of Voxels with abnormal T2	%	quantitative		Mean, Median, Min, Max
Triceps water T2 heterogeneity	CV	quantitative		Mean, Median, Min, Max
Biceps Cross-Section	cm²	quantitative		Mean, Median, Min, Max
Biceps Muscle Tissue Cross-Section	cm²	quantitative		Mean, Median, Min, Max
Biceps Muscle Volume	cm3	quantitative		Mean, Median, Min, Max
Biceps Fat Percentage	%	quantitative		Mean, Median, Min, Max
Biceps T2 mean	msec	quantitative		Mean, Median, Min, Max
Biceps Percentage of Voxels with abnormal T2	%	quantitative		Mean, Median, Min, Max
Biceps water T2 heterogeneity	CV	quantitative		Mean, Median, Min, Max
Flexor Cross-Section	cm²	quantitative		Mean, Median, Min, Max
Flexor Muscle Tissue Cross-Section	cm²	quantitative		Mean, Median, Min, Max
Flexor Muscle Volume	cm3	quantitative		Mean, Median, Min, Max
Flexor Fat Percentage	%	quantitative		Mean, Median, Min, Max
Flexor T2 mean	msec	quantitative		Mean, Median, Min, Max
Flexor Percentage of Voxels with abnormal T2	%	quantitative		Mean, Median, Min, Max
Flexor water T2 heterogeneity	CV	quantitative		Mean, Median, Min, Max
Extensor Cross-Section	cm²	quantitative		Mean, Median, Min, Max
Extensor Muscle Tissue Cross-Section	cm²	quantitative		Mean, Median, Min, Max
Extensor Muscle Volume	cm3	quantitative		Mean, Median, Min, Max

Label	Unit	Type	value	summary of data
Extensor Fat Percentage	%	quantitative		Mean, Median, Min, Max
Extensor T2 mean	msec	quantitative		Mean, Median, Min, Max
Extensor Percentage of Voxels with abnormal T2	%	quantitative		Mean, Median, Min, Max
Extensor water T2 heterogeneity	CV	quantitative		Mean, Median, Min, Max
Quadriceps Cross-Section	cm²	quantitative		Mean, Median, Min, Max
Quadriceps Muscle Tissue Cross-Section	cm²	quantitative		Mean, Median, Min, Max
Quadriceps Muscle Volume	cm3	quantitative		Mean, Median, Min, Max
Quadriceps Fat Percentage	%	quantitative		Mean, Median, Min, Max
Quadriceps T2 mean	msec	quantitative		Mean, Median, Min, Max
Quadriceps Percentage of Voxels with abnormal T2	%	quantitative		Mean, Median, Min, Max
Quadriceps water T2 heterogeneity	CV	quantitative		Mean, Median, Min, Max
Biceps femoris Cross-Section	cm²	quantitative		Mean, Median, Min, Max
Biceps femoris Muscle Tissue Cross-Section	cm²	quantitative		Mean, Median, Min, Max
Biceps femoris Muscle Volume	cm3	quantitative		Mean, Median, Min, Max
Biceps femoris Fat Percentage	%	quantitative		Mean, Median, Min, Max
Biceps femoris T2 mean	msec	quantitative		Mean, Median, Min, Max
Biceps femoris Percentage of Voxels with abnormal T2	%	quantitative		Mean, Median, Min, Max
Biceps femoris water T2 heterogeneity	CV	quantitative		Mean, Median, Min, Max
Semi-Tendinosus Cross-Section	cm²	quantitative		Mean, Median, Min, Max
Semi-Tendinosus Muscle Tissue Cross-Section	cm²	quantitative		Mean, Median, Min, Max
Semi-Tendinosus Muscle Volume	cm3	quantitative		Mean, Median, Min, Max
Semi-Tendinosus Fat Percentage	%	quantitative		Mean, Median, Min, Max
Semi-Tendinosus T2 mean	msec	quantitative		Mean, Median, Min, Max
Semi-Tendinosus Percentage of Voxels with abnormal T2	%	quantitative		Mean, Median, Min, Max
Semi-Tendinosus water T2 heterogeneity	CV	quantitative		Mean, Median, Min, Max
Semi-Membranosus Cross-Section	cm²	quantitative		Mean, Median, Min, Max
Semi-Membranosus Muscle Tissue Cross-Section	cm²	quantitative		Mean, Median, Min, Max
Semi-Membranosus Muscle Volume	cm3	quantitative		Mean, Median, Min, Max
Semi-Membranosus Fat Percentage	%	quantitative		Mean, Median, Min, Max
Semi-Membranosus T2 mean	msec	quantitative		Mean, Median, Min, Max
Semi-Membranosus Percentage of Voxels with abnormal T2	%	quantitative		Mean, Median, Min, Max
Semi-Membranosus water T2 heterogeneity	CV	quantitative		Mean, Median, Min, Max
Triceps Suralis Cross-Section	cm²	quantitative		Mean, Median, Min, Max
Triceps Suralis Muscle Tissue Cross-Section	cm²	quantitative		Mean, Median, Min, Max
Triceps Suralis Muscle Volume	cm3	quantitative		Mean, Median, Min, Max

Label	Unit	Type	value	summary of data
Triceps Suralis Fat Percentage	%	quantitative		Mean, Median, Min, Max
Triceps Suralis T2 mean	msec	quantitative		Mean, Median, Min, Max
Triceps Suralis Percentage of Voxels with abnormal T2	%	quantitative		Mean, Median, Min, Max
Triceps Suralis water T2 heterogeneity	CV	quantitative		Mean, Median, Min, Max
Tibilialis Anterior Cross-Section	cm²	quantitative		Mean, Median, Min, Max
Tibilialis Anterior Muscle Tissue Cross-Section	cm²	quantitative		Mean, Median, Min, Max
Tibilialis Anterior Muscle Volume	cm3	quantitative		Mean, Median, Min, Max
Tibilialis Anterior Fat Percentage	%	quantitative		Mean, Median, Min, Max
Tibilialis Anterior T2 mean	msec	quantitative		Mean, Median, Min, Max
Tibilialis Anterior Percentage of Voxels with abnormal T2	%	quantitative		Mean, Median, Min, Max
Tibilialis Anterior water T2 heterogeneity	CV	quantitative		Mean, Median, Min, Max
Peroneus Lateralis Cross-Section	cm²	quantitative		Mean, Median, Min, Max
Peroneus Lateralis Muscle Tissue Cross-Section	cm²	quantitative		Mean, Median, Min, Max
Peroneus Lateralis Muscle Volume	cm3	quantitative		Mean, Median, Min, Max
Peroneus Lateralis Fat Percentage	%	quantitative		Mean, Median, Min, Max
Peroneus Lateralis T2 mean	msec	quantitative		Mean, Median, Min, Max
Peroneus Lateralis Percentage of Voxels with abnormal T2	%	quantitative		Mean, Median, Min, Max
Peroneus Lateralis water T2 heterogeneity	CV	quantitative		Mean, Median, Min, Max
Blood sample collection	Descriptive statistics at baseline and changes over the study period + comparison between SMA type. On the whole population and by SMA type			
Expression levels of different SMN isoforms		quantitative		Mean, Median, Min, Max
SMN2 copy number	#	quantitative	2 to 4?	Mean, Median, Min, Max
SMN1 mRNA level		quantitative		Mean, Median, Min, Max
SMNd7 mRNA level		quantitative		Mean, Median, Min, Max
SMN protein level		quantitative		Mean, Median, Min, Max
Exploratory biomarkers (muscle damage, IGF pathway or exploratory profiling technology				

Table 2: List of BDD variables (date: 06/11/2017)

Identifiant	Libellé	Type
act_activity_time	Activity (% of time)	FLOAT
act_comment	If not, please comment	VARCHAR
act_completed	Test performed	VARCHAR
act_date	Assessment date	DATE
act_evaluator	Evaluator	INT
calc_act_evaluator	Evaluator name	VARCHAR
act_nacc_95thpercentile	nAcc 95th percentile (G)	FLOAT
act_nacc_mean	nAcc Mean (G)	FLOAT
act_ngyr_95thpercentile	nGyr 95th percentile (°/sec)	FLOAT
act_ngyr_mean	Norm of the angular velocity of the wrist value (°/sec)	FLOAT
act_power_95thpercentile	Power 95th percentile (W/Kg)	FLOAT
act_power_mean	Power Mean (W/Kg)	FLOAT
act_quest_validated	Questionnaire validated	VARCHAR
act_record_days	Recording duration (days)	INT
act_record_end	Recording Period: end date	DATE
act_record_hours	Recording duration (hours)	INT
act_record_start	Recording Period: start date	DATE
act_wrist_side	Wrist Sensor Side	VARCHAR
act_zacc_95thpercentile	zAcc 95th percentile (G)	FLOAT
act_zacc_mean	zAcc Mean (G)	FLOAT
ae_dateonset	Date of Onset	DATE
ae_dateresolv	Date Resolved	DATE
ae_event	Event	INT
calc_ae_event	Event	VARCHAR
ae_final	Final Outcome	VARCHAR
ae_hospend	Hospitalization end date	DATE
ae_hospongoing	Ongoing	VARCHAR
ae_hospstart	Hospitalization start date	DATE
ae_imputability	Imputability	VARCHAR
ae_imputability_detail	If other, please specify	VARCHAR
ae_intensity	Intensity	VARCHAR
ae_ongoing	Ongoing	VARCHAR
ae_sae	Is it an SAE	VARCHAR
ae_sae_associatedpatho	Relevant Associated pathology	VARCHAR
ae_sae_associatedpatho_detail	Description	TEXT
ae_sae_comment	SAE detail	VARCHAR
ae_sae_completedescription	SAE Complete description	TEXT
ae_sae_concottt	Concomitant treatments	VARCHAR
ae_sae_deathautopsy	Autopsy	VARCHAR
ae_sae_deathdate	Date of Death	DATE
ae_sae_deathreason	Reason for Death	VARCHAR
ae_sae_evolution	SAE Evolution	VARCHAR

Identifiant	Libellé	Type
ae_sae_evolution_comment	Comment	TEXT
ae_sae_evolution_recoverydate	Date of Recovery	DATE
ae_sae_evolution_recoverytime	Time of Recovery	TIME
ae_sae_evolution_sequelae	Specify Sequelae	VARCHAR
ae_sae_expected	According to the sponsor, SAE is expected	VARCHAR
ae_sae_intensity	SAE Intensity	VARCHAR
ae_sae_medhist	Relevant Medical History	VARCHAR
ae_sae_medhist_detail	Description	TEXT
ae_sae_onsetdate	Date of Onset	DATE
ae_sae_onsettime	Time of Onset	TIME
ae_sae_other	If other, please specify	VARCHAR
ae_sae_relation	According to the investigator, SAE seems to be related to	VARCHAR
ae_sae_relation_disease	Specify intercurrent disease	VARCHAR
ae_sae_relation_disease_sponsor	Specify intercurrent disease	VARCHAR
ae_sae_relation_other	If other, please specify	VARCHAR
ae_sae_relation_other_sponsor	If other, please specify	VARCHAR
ae_sae_relation_sponsor	According to the sponsor, SAE seems to be related to	VARCHAR
ae_sae_relation_study	Specify study process	VARCHAR
ae_sae_relation_study_sponsor	Specify study process	VARCHAR
ae_sae_relation_treatment	Specify concomitant treatment	VARCHAR
ae_sae_relation_treatment_sponsor	Specify concomitant treatment	VARCHAR
ae_sae_sponsorcomment	Sponsor Comments	TEXT
ae_sae_supexams	Supplementary Exams	VARCHAR
ae_sae_supexams_comment	Comment	TEXT
ae_sae_supexams1_date	Date	DATE
ae_sae_supexams1_detail	Exam	VARCHAR
ae_sae_supexams1_results	Results	VARCHAR
ae_sae_supexams1_sign	Significant	VARCHAR
ae_sae_supexams2_date	Date	DATE
ae_sae_supexams2_detail	Exam	VARCHAR
ae_sae_supexams2_results	Results	VARCHAR
ae_sae_supexams2_sign	Significant	VARCHAR
ae_sae_supexams3_date	Date	DATE
ae_sae_supexams3_detail	Exam	VARCHAR
ae_sae_supexams3_results	Results	VARCHAR
ae_sae_supexams3_sign	Significant	VARCHAR
ae_sae_supexams4_date	Date	DATE
ae_sae_supexams4_detail	Exam	VARCHAR
ae_sae_supexams4_results	Results	VARCHAR
ae_sae_supexams4_sign	Significant	VARCHAR
ae_sae_supexams5_date	Date	DATE
ae_sae_supexams5_detail	Exam	VARCHAR
ae_sae_supexams5_results	Results	VARCHAR
ae_sae_supexams5_sign	Significant	VARCHAR
ae_sae_supexams6_date	Date	DATE

Identifiant	Libellé	Type
ae_sae_supexams6_detail	Exam	VARCHAR
ae_sae_supexams6_results	Results	VARCHAR
ae_sae_supexams6_sign	Significant	VARCHAR
ae_sae_supexams7_date	Date	DATE
ae_sae_supexams7_detail	Exam	VARCHAR
ae_sae_supexams7_results	Results	VARCHAR
ae_sae_supexams7_sign	Significant	VARCHAR
ae_sae_supexams8_date	Date	DATE
ae_sae_supexams8_detail	Exam	VARCHAR
ae_sae_supexams8_results	Results	VARCHAR
ae_sae_supexams8_sign	Significant	VARCHAR
ae_suppexam	Supplementary exams	VARCHAR
ae_suppexamcomment	Specify	VARCHAR
ae_treatment	AE Treatment	VARCHAR
bio_date	Collection Date	DATE
bio_edta_performed	EDTA tube collected	VARCHAR
bio_edta_performed_reason	If no, please comment	VARCHAR
bio_p700_performed	P700 tube collected	VARCHAR
bio_p700_performed_reason	If no, please comment	VARCHAR
bio_pax_performed	PaxGene tube collected	VARCHAR
bio_pax_performed_reason	If no, please comment	VARCHAR
bio_suppdna_performed	Supplementary DNA tube collected	VARCHAR
bio_supp_dna_performed_reason	If no, please specify the reason why tube not collected	VARCHAR
bio_performed	Blood samples performed	VARCHAR
bio_quest_validated	Questionnaire validated	VARCHAR
bio_reason	If not, please comment	VARCHAR
bio_s1copy_analysisdate	Analysis Date	DATE
bio_s1copy_analysismethod	Analysis Method	VARCHAR
bio_s1copy_comments	Comments	VARCHAR
bio_s1copy_labname	Lab Name	VARCHAR
bio_s1copy_result	mRNA level	FLOAT
bio_s1rna_analysisdate	Analysis Date	DATE
bio_s1rna_analysismethod	Analysis Method	VARCHAR
bio_s1rna_comments	Comments	VARCHAR
bio_s1rna_labname	Lab Name	VARCHAR
bio_s1rna_result	mRNA level	FLOAT
bio_s2copy_analysisdate	Analysis Date	DATE
bio_s2copy_analysismethod	Analysis Method	VARCHAR
bio_s2copy_comments	Comments	VARCHAR
bio_s2copy_labname	Lab Name	VARCHAR
bio_s2copy_result	Copy Number	INT
bio_s2rna_analysisdate	Analysis Date	DATE
bio_s2rna_analysismethod	Analysis Method	VARCHAR
bio_s2rna_comments	Comments	VARCHAR
bio_s2rna_labname	Lab Name	VARCHAR

Identifiant	Libellé	Type
bio_s2rna_result	mRNA level	FLOAT
bio_sd7rna_analysisdate	Analysis Date	DATE
bio_sd7rna_analysismethod	Analysis Method	VARCHAR
bio_sd7rna_comments	Comments	VARCHAR
bio_sd7rna_labname	Lab Name	VARCHAR
bio_sd7rna_result	mRNA Level	FLOAT
bio_serum_performed	Serum tube collected	VARCHAR
bio_serum_performed_reason	If no, please comment	VARCHAR
bio_sprotein_analysisdate	Analysis Date	DATE
bio_sprotein_analysismethod	Analysis Method	VARCHAR
bio_sprotein_comments	Comments	VARCHAR
bio_sprotein_labname	Lab Name	VARCHAR
bio_sprotein_result	SMN Protein (pg/mL)	FLOAT
bio_srgrna_analysisdate	Analysis Date	DATE
bio_srgrna_analysismethod	Analysis Method	VARCHAR
bio_srgrna_comments	Comments	VARCHAR
bio_srgrna_labname	Lab Name	VARCHAR
bio_srgrna_result	mRNA level	FLOAT
ds_identification	ID	VARCHAR
ds_identification	ID	VARCHAR
emg_acc_1cmap_amp	Amplitude (mV)	FLOAT
emg_acc_1cmap_area	Area (mV.msec)	FLOAT
emg_acc_5_1cmap_amp	Amplitude (%)	FLOAT
emg_acc_5_1cmap_area	Area (%)	FLOAT
emg_acc_5cmap_amp	Amplitude (mV)	FLOAT
emg_acc_5cmap_area	Area (mV.msec)	FLOAT
emg_acc_dist	Distance (mm)	FLOAT
emg_acc_temp	Temperature (°C)	FLOAT
emg_acc_trap_motor	Distal Motor Latency (msec)	FLOAT
emg_cmap	CMAP and decrement search performed	VARCHAR
emg_date	Assessment Date	DATE
emg_peroneal_1cmap_amp	Amplitude (mV)	FLOAT
emg_peroneal_1cmap_area	Area (mV.msec)	FLOAT
emg_peroneal_5_1cmap_amp	Amplitude (%)	FLOAT
emg_peroneal_5_1cmap_area	Area (%)	FLOAT
emg_peroneal_5cmap_amp	Amplitude (mV)	FLOAT
emg_peroneal_5cmap_area	Area (mV.msec)	FLOAT
emg_peroneal_dist	Distance (mm)	FLOAT
emg_peroneal_motor	Distal Motor Latency (msec)	FLOAT
emg_peroneal_temp	Temperature (°C)	FLOAT
emg_quest_validated	Questionnaire validated	VARCHAR
emg_radial_1cmap_amp	Amplitude (mV)	FLOAT
emg_radial_1cmap_area	Area (mV.msec)	FLOAT
emg_radial_5_1cmap_amp	Amplitude (%)	FLOAT
emg_radial_5_1cmap_area	Area (%)	FLOAT

Identifiant	Libellé	Type
emg_radial_5cmap_amp	Amplitude (mV)	FLOAT
emg_radial_5cmap_area	Area (mV.msec)	FLOAT
emg_radial_dist	Distance (mm)	FLOAT
emg_radial_motor	Distal Motor Latency (msec)	FLOAT
emg_radial_temp	Temperature (°C)	FLOAT
emg_reason	If not, please comment	VARCHAR
emg_side	Tested Side	VARCHAR
emg_ulnar_1cmap_amp	Amplitude (mV)	FLOAT
emg_ulnar_1cmap_area	Area (mV.msec)	VARCHAR
emg_ulnar_5_1cmap_amp	Amplitude (%)	FLOAT
emg_ulnar_5_1cmap_area	Area (%)	FLOAT
emg_ulnar_5cmap_amp	Amplitude (mV)	FLOAT
emg_ulnar_5cmap_area	Area (mV.msec)	FLOAT
emg_ulnar_dist	Distance (mm)	FLOAT
emg_ulnar_motor	Distal Motor Latency (msec)	FLOAT
emg_ulnar_temp	Temperature (°C)	FLOAT
fct_active_area1	% Area	FLOAT
fct_active_area2	% Area	FLOAT
fct_active_area3	% Area	FLOAT
fct_active_completed	Test performed	VARCHAR
fct_active_completed_reason	If not, please comment	VARCHAR
fct_active_completed_reason_other	If other, please specify	VARCHAR
fct_active_software	Software version	VARCHAR
fct_active_date	Assessment Date	DATE
fct_active_evaluator	Evaluator	INT
calc_fct_active_evaluator	Evaluator	VARCHAR
fct_active_surface1	Surface Area	FLOAT
fct_active_surface2	Surface Area	FLOAT
fct_active_surface3	Surface Area	FLOAT
fct_active_totalvol_pred1	% Volume	FLOAT
fct_active_totalvol_pred2	% Volume	FLOAT
fct_active_totalvol_pred3	% Volume	FLOAT
fct_active_totalvol1	Total Volume	FLOAT
fct_active_totalvol2	Total Volume	FLOAT
fct_active_totalvol3	Total Volume	FLOAT
fct_active_trunk_fwd1	Trunk Forward	FLOAT
fct_active_trunk_fwd2	Trunk Forward	FLOAT
fct_active_trunk_fwd3	Trunk Forward	FLOAT
fct_active_trunk_left1	Trunk Left	FLOAT
fct_active_trunk_left2	Trunk Left	FLOAT
fct_active_trunk_left3	Trunk Left	FLOAT
fct_active_trunk_right1	Trunk Right	FLOAT
fct_active_trunk_right2	Trunk Right	FLOAT
fct_active_trunk_right3	Trunk Right	FLOAT
fct_active_ulnalength_average	Ulna Length (cm)	CALC

Identifiant	Libellé	Type
fct_date	Assessment Date	DATE
fct_dominant_hand	Dominant Hand	VARCHAR
fct_mfm_1	Item #1	INT
fct_mfm_10	Item #10	INT
fct_mfm_11	Item #11	INT
fct_mfm_12	Item #12	INT
fct_mfm_13	Item #13	INT
fct_mfm_14	Item #14	INT
fct_mfm_15	Item #15	INT
fct_mfm_16	Item #16	INT
fct_mfm_17	Item #17	INT
fct_mfm_18	Item #18	INT
fct_mfm_19	Item #19	INT
fct_mfm_2	Item #2	INT
fct_mfm_20	Item #20	INT
fct_mfm_21	Item #21	INT
fct_mfm_22	Item #22	INT
fct_mfm_23	Item #23	INT
fct_mfm_24	Item #24	INT
fct_mfm_25	Item #25	INT
fct_mfm_26	Item #26	INT
fct_mfm_27	Item #27	INT
fct_mfm_28	Item #28	INT
fct_mfm_29	Item #29	INT
fct_mfm_3	Item #3	INT
fct_mfm_30	Item #30	INT
fct_mfm_31	Item #31	INT
fct_mfm_32	Item #32	INT
fct_mfm_4	Item #4	INT
fct_mfm_5	Item #5	INT
fct_mfm_6	Item #6	INT
fct_mfm_7	Item #7	INT
fct_mfm_8	Item #8	INT
fct_mfm_9	Item #9	INT
fct_mfm_comments	Particularities during MFM test	VARCHAR
fct_mfm_completed	MFM Test completed	VARCHAR
fct_mfm_completed_reason	If not, please comment	VARCHAR
fct_mfm_completed_reason_other	If other, please specify	VARCHAR
fct_mfm_cooperation	Patient Cooperation	VARCHAR
fct_mfm_d1_mfm20	D1 Score (%)	CALC
fct_mfm_d1_mfm32	D1 Score (%)	CALC
fct_mfm_d2_mfm20	D2 Score (%)	CALC
fct_mfm_d2_mfm32	D2 Score (%)	CALC
fct_mfm_d3_mfm20	D3 Score (%)	CALC
fct_mfm_d3_mfm32	D3 Score (%)	CALC

Identifiant	Libellé	Type
fct_mfm_date	Assessment Date	DATE
fct_mfm_evaluator	Evaluator Name	INT
calc_fct_mfm_evaluator	Evaluator	VARCHAR
fct_mfm_total_mfm20	Total Score (%)	CALC
fct_mfm_total_mfm32	Total Score (%)	CALC
fct_mfm_type	MFM Test completed	VARCHAR
fct_mvp_comments	Comments	VARCHAR
fct_mvp_completed	Moviplate test performed	VARCHAR
fct_mvp_completed_reason	If not, please comment	VARCHAR
fct_mvp_completed_reason_other	If other, please specify	VARCHAR
fct_mvp_date	Assessment Date	DATE
fct_mvp_evaluator	Evaluator Name	INT
calc_fct_mvp_evaluator	Evaluator	VARCHAR
fct_mvp_trial1_dom_result	Dominant Side Result Trial #1	INT
fct_mvp_trial1_dom_valid	Valid or Invalid	VARCHAR
fct_mvp_trial1_nondom_result	Non Dominant Side Result Trial #1	INT
fct_mvp_trial1_nondom_valid	Valid or Invalid	VARCHAR
fct_mvp_trial2_dom_result	Dominant Side Result Trial #2	INT
fct_mvp_trial2_dom_valid	Valid or Invalid	VARCHAR
fct_mvp_trial2_nondom_result	Non Dominant Side Result Trial #2	INT
fct_mvp_trial2_nondom_valid	Valid or Invalid	VARCHAR
fct_mvp_trial3_dom_result	Dominant Side Result Trial #3	INT
fct_mvp_trial3_dom_valid	Valid or Invalid	VARCHAR
fct_mvp_trial3_nondom_result	Non Dominant Side Result Trial #3	INT
fct_mvp_trial3_nondom_valid	Valid or Invalid	VARCHAR
fct_quest_validated	Questionnaire validated	VARCHAR
nmr_arm_side	Arm Tested Side	VARCHAR
nmr_biceps_csa	Biceps Cross-Section (mm2)	FLOAT
nmr_biceps_csamuscle	Biceps Muscle Tissue Cross-Section (mm2)	FLOAT
nmr_biceps_ff	Biceps Fat Fraction (%)	FLOAT
nmr_biceps_t2abnn	Biceps Fraction of Voxels with abnormal T2 (%)	FLOAT
nmr_biceps_t2cv	Biceps water T2 heterogeneity (CV)	FLOAT
nmr_biceps_t2mean	Biceps T2 mean (ms)	FLOAT
nmr_bicepsfem_ff	Biceps femoris Fat Fraction (%)	FLOAT
nmr_bicepsfem_t2abn	Biceps femoris Fraction of Voxels with abnormal T2 (%)	FLOAT
nmr_bicepsfem_t2cv	Biceps femoris water T2 heterogeneity (CV)	FLOAT
nmr_bicepsfem_t2mean	Biceps femoris T2 mean (ms)	FLOAT
nmr_completed	NMR performed	VARCHAR
nmr_date	Assessment Date	DATE
nmr_extensor_csa	Extensor Cross-Section (mm2)	FLOAT
nmr_extensor_csamuscle	Extensor Muscle Tissue Cross-Section (mm2)	FLOAT
nmr_extensor_ff	Extensor Fat Fraction (%)	FLOAT
nmr_extensor_t2abn	Extensor Fraction of Voxels with abnormal T2 (%)	FLOAT
nmr_extensor_t2cv	Extensor water T2 heterogeneity (CV)	FLOAT
nmr_extensor_t2mean	Extensor T2 mean (ms)	FLOAT

Identifiant	Libellé	Type
nmr_extensorim_csa	Leg extensor Cross-Section (mm2)	FLOAT
nmr_extensorim_csamuscle	Leg extensor Tissue Cross-Section (mm2)	FLOAT
nmr_flexor_csa	Flexor Cross-Section (mm2)	FLOAT
nmr_flexor_csamuscle	Flexor Muscle Tissue Cross-Section (mm2)	FLOAT
nmr_flexor_ff	Flexor Fat Fraction (%)	FLOAT
nmr_flexor_t2abn	Flexor Fraction of Voxels with abnormal T2 (%)	FLOAT
nmr_flexor_t2cv	Flexor water T2 heterogeneity (CV)	FLOAT
nmr_flexor_t2mean	Flexor T2 mean (ms)	FLOAT
nmr_hamstring_csa	Hamstring Cross-Section (mm2)	FLOAT
nmr_hamstring_csamuscle	Hamstring Tissue Cross-Section (mm2)	FLOAT
nmr_performed_reason	If not, please comment	VARCHAR
nmr_peroneuslat_csa	Peroneus Lateralis Cross-Section (mm2)	FLOAT
nmr_peroneuslat_csamuscle	Peroneus Lateralis Muscle Tissue Cross-Section (mm2)	FLOAT
nmr_peroneuslat_ff	Peroneus Lateralis Fat Fraction (%)	FLOAT
nmr_peroneuslat_t2abn	Peroneus Lateralis Fraction of Voxels with abnormal T2 (%)	FLOAT
nmr_peroneuslat_t2cv	Peroneus Lateralis water T2 heterogeneity (CV)	FLOAT
nmr_peroneuslat_t2mean	Peroneus Lateralis T2 mean (ms)	FLOAT
nmr_quadriceps_csa	Quadriceps Cross-Section (mm2)	FLOAT
nmr_quadriceps_csamuscle	Quadriceps Muscle Tissue Cross-Section (mm2)	FLOAT
nmr_quadriceps_ff	Quadriceps Fat Fraction (%)	FLOAT
nmr_quadriceps_t2abn	Quadriceps Fraction of Voxels with abnormal T2 (%)	FLOAT
nmr_quadriceps_t2cv	Quadriceps water T2 heterogeneity (CV)	FLOAT
nmr_quadriceps_t2mean	Quadriceps T2 mean (ms)	FLOAT
nmr_semimemb_ff	Semi-Membranosus Fat Fraction (%)	FLOAT
nmr_semimemb_t2abn	Semi-Membranosus Fraction of Voxels with abnormal T2 (%)	FLOAT
nmr_semimemb_t2cv	Semi-Membranosus water T2 heterogeneity (CV)	FLOAT
nmr_semimemb_t2mean	Semi-Membranosus T2 mean (ms)	FLOAT
nmr_semitend_ff	Semi-Tendinosus Fat Fraction (%)	FLOAT
nmr_semitend_t2abn	Semi-Tendinosus Fraction of Voxels with abnormal T2 (%)	FLOAT
nmr_semitend_t2cv	Semi-Tendinosus water T2 heterogeneity (CV)	FLOAT
nmr_semitend_t2mean	Semi-Tendinosus T2 mean (ms)	FLOAT
nmr_tibialisant_ff	Tibilialis Anterior Fat Fraction (%)	FLOAT
nmr_tibialisant_t2abn	Tibilialis Anterior Fraction of Voxels with abnormal T2 (%)	FLOAT
nmr_tibialisant_t2cv	Tibilialis Anterior water T2 heterogeneity (CV)	FLOAT
nmr_tibialisant_t2mean	Tibilialis Anterior T2 mean (ms)	FLOAT
nmr_triceps_csa	Triceps Cross-Section (mm2)	FLOAT
nmr_triceps_csamuscle	Triceps Muscle Tissue Cross-Section (mm2)	FLOAT
nmr_triceps_ff	Triceps Fat Fraction (%)	FLOAT
nmr_triceps_t2abnn	Triceps Fraction of Voxels with abnormal T2 (%)	FLOAT
nmr_triceps_t2cv	Triceps water T2 heterogeneity (CV)	FLOAT
nmr_triceps_t2mean	Triceps T2 mean (ms)	FLOAT
nmr_tricepssur_csa	Triceps Suralis Cross-Section (mm2)	FLOAT
nmr_tricepssur_csamuscle	Triceps Suralis Muscle Tissue Cross-Section (mm2)	FLOAT
nmr_tricepssur_ff	Triceps Suralis Fat Fraction (%)	FLOAT
nmr_tricepssur_t2abn	Triceps Suralis Fraction of Voxels with abnormal T2 (%)	FLOAT

Identifiant	Libellé	Type
nmr_tricepssur_t2cv	Triceps Suralis water T2 heterogeneity (CV)	FLOAT
nmr_tricepssur_t2mean	Triceps Suralis T2 mean (ms)	FLOAT
nmr_spine_ratio_diameters	Ratio of L-R and A-P diameters	FLOAT
nmr_spine_ratio_segments	Ratio of P to A segments	FLOAT
p_atcd_agesymptome	Age of first symptoms	INT
p_atcd_birth	Birth at (weeks of amenorrhea)	INT
p_atcd_cardio	Cardiovascular and blood	VARCHAR
p_atcd_cardio1	Event 1	VARCHAR
p_atcd_cardio1date	Date	DATE
p_atcd_cardio2	Event 2	VARCHAR
p_atcd_cardio2date	Date	DATE
p_atcd_cardio3	Event 3	VARCHAR
p_atcd_cardio3date	Date	DATE
p_atcd_dategenconfirm	Date of Genetic confirmation	DATE
p_atcd_delivery	Type of delivery	VARCHAR
p_atcd_diag	Date of Diagnosis	DATE
p_atcd_eent	Eye/Ear/Nose/Throat	VARCHAR
p_atcd_eent1	Event 1	VARCHAR
p_atcd_eent10	Event 10	VARCHAR
p_atcd_eent10date	Date	DATE
p_atcd_eent1date	Date	DATE
p_atcd_eent2	Event 2	VARCHAR
p_atcd_eent2date	Date	DATE
p_atcd_eent3	Event 3	VARCHAR
p_atcd_eent3date	Date	DATE
p_atcd_eent4	Event 4	VARCHAR
p_atcd_eent4date	Date	DATE
p_atcd_eent5	Event 5	VARCHAR
p_atcd_eent5date	Date	DATE
p_atcd_eent6	Event 6	VARCHAR
p_atcd_eent6date	Date	DATE
p_atcd_eent7	Event 7	VARCHAR
p_atcd_eent7date	Date	DATE
p_atcd_eent8	Event 8	VARCHAR
p_atcd_eent8date	Date	DATE
p_atcd_eent9	Event 9	VARCHAR
p_atcd_eent9data	Date	DATE
p_atcd_family	Other member of the family affected by the disease	VARCHAR
p_atcd_familycomment	Which member	VARCHAR
p_atcd_genconfirm	Genetic confirmation	VARCHAR
p_atcd_genconfirmesult	Results	VARCHAR
p_atcd_genito	Genitourinary	VARCHAR
p_atcd_genito1	Event 1	VARCHAR
p_atcd_genito1date	Date	DATE
p_atcd_genito2	Event 2	VARCHAR

Identifiant	Libellé	Type
p_atcd_genito2date	Date	DATE
p_atcd_genito3	Event 3	VARCHAR
p_atcd_genito3date	Date	DATE
p_atcd_head	Head circumference (cm)	FLOAT
p_atcd_hepato	Hepatic-gastrointestinal	VARCHAR
p_atcd_hepato1	Event 1	VARCHAR
p_atcd_hepato1date	Date	DATE
p_atcd_hepato2	Event 2	VARCHAR
p_atcd_hepato2date	Date	DATE
p_atcd_hepato3	Event 3	VARCHAR
p_atcd_hepato3date	Date	DATE
p_atcd_hepato4	Event 4	VARCHAR
p_atcd_hepato4date	Date	DATE
p_atcd_hospitsma	Previous hospitalizations in relation with SMA	VARCHAR
p_atcd_hospitsma1	Hospitalization	VARCHAR
p_atcd_hospitsma10	Hospitalization	VARCHAR
p_atcd_hospitsma10date	Date	DATE
p_atcd_hospitsma10dur	Duration (days)	INT
p_atcd_hospitsma11	Hospitalization	VARCHAR
p_atcd_hospitsma11date	Date	DATE
p_atcd_hospitsma11dur	Duration (days)	INT
p_atcd_hospitsma12	Hospitalization	VARCHAR
p_atcd_hospitsma12date	Date	DATE
p_atcd_hospitsma12dur	Duration (days)	INT
p_atcd_hospitsma13	Hospitalization	VARCHAR
p_atcd_hospitsma13date	Date	DATE
p_atcd_hospitsma13dur	Duration (days)	INT
p_atcd_hospitsma14	Hospitalization	VARCHAR
p_atcd_hospitsma14date	Date	DATE
p_atcd_hospitsma14dur	Duration (days)	INT
p_atcd_hospitsma15	Hospitalization	VARCHAR
p_atcd_hospitsma15date	Date	DATE
p_atcd_hospitsma15dur	Duration (days)	INT
p_atcd_hospitsma16	Hospitalization	VARCHAR
p_atcd_hospitsma16date	Date	DATE
p_atcd_hospitsma16dur	Duration (days)	INT
p_atcd_hospitsma17	Hospitalization	VARCHAR
p_atcd_hospitsma17date	Date	DATE
p_atcd_hospitsma17dur	Duration (days)	INT
p_atcd_hospitsma18	Hospitalization	VARCHAR
p_atcd_hospitsma18date	Date	DATE
p_atcd_hospitsma18dur	Duration (days)	INT
p_atcd_hospitsma19	Hospitalization	VARCHAR
p_atcd_hospitsma19date	Date	DATE
p_atcd_hospitsma19dur	Duration (days)	INT

Identifiant	Libellé	Type
p_atcd_hospitsma1date	Date	DATE
p_atcd_hospitsma1dur	Duration (days)	INT
p_atcd_hospitsma2	Hospitalization	VARCHAR
p_atcd_hospitsma20	Hospitalization	VARCHAR
p_atcd_hospitsma20date	Date	DATE
p_atcd_hospitsma20dur	Duration (days)	INT
p_atcd_hospitsma21	Hospitalization	VARCHAR
p_atcd_hospitsma21date	Date	DATE
p_atcd_hospitsma21dur	Duration (days)	INT
p_atcd_hospitsma22	Hospitalization	VARCHAR
p_atcd_hospitsma22date	Date	DATE
p_atcd_hospitsma22dur	Duration (days)	INT
p_atcd_hospitsma23	Hospitalization	VARCHAR
p_atcd_hospitsma23date	Date	DATE
p_atcd_hospitsma23dur	Duration (days)	INT
p_atcd_hospitsma24	Hospitalization	VARCHAR
p_atcd_hospitsma24date	Date	DATE
p_atcd_hospitsma24dur	Duration (days)	INT
p_atcd_hospitsma25	Hospitalization	VARCHAR
p_atcd_hospitsma25date	Date	DATE
p_atcd_hospitsma25dur	Duration (days)	INT
p_atcd_hospitsma26	Hospitalization	VARCHAR
p_atcd_hospitsma26date	Date	DATE
p_atcd_hospitsma26dur	Duration (days)	INT
p_atcd_hospitsma27	Hospitalization	VARCHAR
p_atcd_hospitsma27date	Date	DATE
p_atcd_hospitsma27dur	Duration (days)	INT
p_atcd_hospitsma2date	Date	DATE
p_atcd_hospitsma2dur	Duration (days)	INT
p_atcd_hospitsma3	Hospitalization	VARCHAR
p_atcd_hospitsma3date	Date	DATE
p_atcd_hospitsma3dur	Duration (days)	INT
p_atcd_hospitsma4	Hospitalization	VARCHAR
p_atcd_hospitsma4date	Date	DATE
p_atcd_hospitsma4dur	Duration (days)	INT
p_atcd_hospitsma5	Hospitalization	VARCHAR
p_atcd_hospitsma5date	Date	DATE
p_atcd_hospitsma5dur	Duration (days)	INT
p_atcd_hospitsma6	Hospitalization	VARCHAR
p_atcd_hospitsma6date	Date	DATE
p_atcd_hospitsma6dur	Duration (days)	INT
p_atcd_hospitsma7	Hospitalization	VARCHAR
p_atcd_hospitsma7date	Date	DATE
p_atcd_hospitsma7dur	Duration (days)	INT
p_atcd_hospitsma8	Hospitalization	VARCHAR

Identifiant	Libellé	Type
p_atcd_hospitsma8date	Date	DATE
p_atcd_hospitsma8dur	Duration (days)	INT
p_atcd_hospitsma9	Hospitalization	VARCHAR
p_atcd_hospitsma9date	Date	DATE
p_atcd_hospitsma9dur	Duration (days)	INT
p_atcd_lab	Laboratory	VARCHAR
p_atcd_length	Length at birth (cm)	FLOAT
p_atcd_metabo	Metabolic	VARCHAR
p_atcd_metabo1	Event 1	VARCHAR
p_atcd_metabo1date	Date	DATE
p_atcd_metabo2	Event 2	VARCHAR
p_atcd_metabo2date	Date	DATE
p_atcd_metabo3	Event 3	VARCHAR
p_atcd_metabo3date	Date	DATE
p_atcd_musculo	Musculoskeletal	VARCHAR
p_atcd_musculo1	Event 1	VARCHAR
p_atcd_musculo1date	Date	DATE
p_atcd_musculo2	Event 2	VARCHAR
p_atcd_musculo2date	Date	DATE
p_atcd_musculo3	Event 3	VARCHAR
p_atcd_musculo3date	Date	DATE
p_atcd_musculo4	Event 4	VARCHAR
p_atcd_musculo4date	Date	DATE
p_atcd_musculo5	Event 5	VARCHAR
p_atcd_musculo5date	Date	DATE
p_atcd_musculo6	Event 5	VARCHAR
p_atcd_musculo6date	Date	DATE
p_atcd_musculo7	Event 5	VARCHAR
p_atcd_musculo7date	Date	DATE
p_atcd_musculo8	Event 5	VARCHAR
p_atcd_musculo8date	Date	DATE
p_atcd_musculo9	Event 5	VARCHAR
p_atcd_musculo9date	Date	DATE
p_atcd_musculo10	Event 5	VARCHAR
p_atcd_musculo10date	Date	DATE
p_atcd_neuropsy	Neurologic and Psychiatric	VARCHAR
p_atcd_neuropsy1	Event 1	VARCHAR
p_atcd_neuropsy1date	Date	DATE
p_atcd_neuropsy2	Event 2	VARCHAR
p_atcd_neuropsy2date	Date	DATE
p_atcd_neuropsy3	Event 3	VARCHAR
p_atcd_neuropsy3date	Date	DATE
p_atcd_pulmo	Pulmonary	VARCHAR
p_atcd_pulmo1	Event 1	VARCHAR
p_atcd_pulmo1date	Date	DATE

Identifiant	Libellé	Type
p_atcd_pulmo2	Event 2	VARCHAR
p_atcd_pulmo2date	Date	DATE
p_atcd_pulmo3	Event 3	VARCHAR
p_atcd_pulmo3date	Date	DATE
p_atcd_pulmo4	Event 4	VARCHAR
p_atcd_pulmo4date	Date	DATE
p_atcd_pulmo5	Event 5	VARCHAR
p_atcd_pulmo5date	Date	DATE
p_atcd_pulmo6	Event 6	VARCHAR
p_atcd_pulmo6date	Date	DATE
p_atcd_pulmo7	Event 7	VARCHAR
p_atcd_pulmo7date	Date	DATE
p_atcd_pulmo8	Event 8	VARCHAR
p_atcd_pulmo8date	Date	DATE
p_atcd_pulmo9	Event 9	VARCHAR
p_atcd_pulmo9date	Date	DATE
p_atcd_skin	Skin	VARCHAR
p_atcd_skin1	Event 1	VARCHAR
p_atcd_skin1date	Date	DATE
p_atcd_skin2	Event 2	VARCHAR
p_atcd_skin2date	Date	DATE
p_atcd_skin3	Event 3	VARCHAR
p_atcd_skin3date	Date	DATE
p_atcd_smatype	SMA Type	VARCHAR
p_atcd_smn2	SMN2 copy number	INT
p_atcd_weight	Birth weight (g)	INT
p_cnt	Site Name	VARCHAR
p_consent	Date of signature	DATE
p_dob	Date of birth	DATE
p_dominant	Dominant Side	VARCHAR
p_eos_compliance	Compliance with the protocol	VARCHAR
p_eos_compliancecomment	Comment	VARCHAR
p_eos_eos	Did the subject complete the study	VARCHAR
p_eos_eosdate	Date of last contact	DATE
p_eos_premastop	Premature stop	VARCHAR
p_eos_premastopreason	Specify	VARCHAR
p_eos_premastopreasonmore	More information	VARCHAR
p_gender	Gender	VARCHAR
p_id	Patient ID	VARCHAR
p_quest_validated	Questionnaire validated	VARCHAR
p_selec_ci1	Inclusion criteria #1	VARCHAR
p_selec_ci2	Inclusion criteria #2	VARCHAR
p_selec_ci3	Inclusion criteria #3	VARCHAR
p_selec_ci4	Inclusion criteria #4	VARCHAR
p_selec_ci5	Inclusion criteria #5	VARCHAR

Identifiant	Libellé	Type
p_selec_ci6	Inclusion criteria #6	VARCHAR
p_selec_cni1	Non-inclusion criteria #1	VARCHAR
p_selec_cni2	Non-inclusion criteria #2	VARCHAR
p_selec_cni3	Non-inclusion criteria #3	VARCHAR
p_selec_cni4	Non-inclusion criteria #4	VARCHAR
p_selec_cni5	Non-inclusion criteria #5	VARCHAR
pe_ae	Occurrence of an AE/SAE	VARCHAR
pe_ambulant	Ambulant	VARCHAR
pe_brooke	Brooke Score (1-6)	INT
pe_cardio	Cardiovascular and blood	VARCHAR
pe_cardio_comment	Comment	VARCHAR
pe_diastolicbp	Diastolic blood pressure	INT
pe_eeent_comment	Comment	VARCHAR
pe_eent	Eye/Ear/Nose/Throat	VARCHAR
pe_genito	Genitourinary	VARCHAR
pe_genito_comment	Comment	VARCHAR
pe_heartrate	Heart Rate (bpm)	INT
pe_height	Height (cm)	FLOAT
pe_height_measure	Measured or reported	VARCHAR
pe_hepato	Hepatic-gastrointestinal	VARCHAR
pe_hepato_comment	Comment	VARCHAR
pe_lelbowext	Elbow Extension	VARCHAR
pe_lelbowflex	Elbow Flexion	VARCHAR
pe_lfingerext	Finger Extension	VARCHAR
pe_lfingerflex	Finger Flexion	VARCHAR
pe_lwristext	Wrist Extension	VARCHAR
pe_lwristflex	Wrist Flexion	VARCHAR
pe_metabo	Metabolic	VARCHAR
pe_metabo_comment	Comment	VARCHAR
pe_musculo	Musculoskeletal	VARCHAR
pe_musculo_comment	Comment	VARCHAR
pe_neuropsy	Neurologic and Psychiatric	VARCHAR
pe_np_comment	Comment	VARCHAR
pe_physio	Physiotherapy	VARCHAR
pe_physio_times	How many times a week	INT
pe_pulmo	Pulmonary	VARCHAR
pe_pulmo_comment	Comment	VARCHAR
pe_quest_validated	Questionnaire validated	VARCHAR
pe_relbowext	Elbow Extension	VARCHAR
pe_relboxflex	Elbow Flexion	VARCHAR
pe_respiratoryrate	Respiratory Rate (breaths per minute)	INT
pe_rfingerext	Finger Extension	VARCHAR
pe_rfingerflex	Finger Flexion	VARCHAR
pe_rwristext	Wrist Extension	VARCHAR
pe_rwristflex	Wrist Flexion	VARCHAR

Identifiant	Libellé	Type
pe_skin	Skin	VARCHAR
pe_skin_comment	Comment	VARCHAR
pe_systolicbp	Systolic blood pressure	INT
pe_temperature	Body Temperature (°C)	FLOAT
pe_ttt	Ongoing treatment(s)	VARCHAR
pe_weight	Weight (kg)	FLOAT
pe_weight_measure	Measured or reported	VARCHAR
pft_completed	Test Completed	VARCHAR
pft_completed_reason	If not, please comment	VARCHAR
pft_completed_reason_other	If other, please specify	VARCHAR
pft_date	Assessment Date	DATE
pft_evaluator	Evaluator Name	INT
calc_pft_evaluator	Evaluator Name	VARCHAR
pft_fvc_attempt1	Best FVC attempt #1	FLOAT
pft_fvc_attempt2	Best FVC attempt #2	FLOAT
pft_fvc_attempt3	Best FVC attempt #3	FLOAT
pft_fvc_bestfvc	% predicted for FVC	INT
pft_fvc_comment	Comments	VARCHAR
pft_fvc_completed	Test Completed	VARCHAR
pft_fvc_reason	If not, please comment	VARCHAR
pft_fvc_reason_other	If other, please specify	VARCHAR
pft_mep_attempt1	MEP #1 (cmH2O)	INT
pft_mep_attempt2	MEP #2 (cmH2O)	INT
pft_mep_attempt3	MEP #3 (cmH2O)	INT
pft_mep_comment	Comments	VARCHAR
pft_mep_completed	Test Completed	VARCHAR
pft_mep_reason	If not, please comment	VARCHAR
pft_mep_reason_other	If other, please specify	VARCHAR
pft_mip_attempt1	MIP #1 (cmH2O)	INT
pft_mip_attempt2	MIP #2 (cmH2O)	INT
pft_mip_attempt3	MIP #3 (cmH2O)	INT
pft_mip_comment	Comments	VARCHAR
pft_mip_completed	Test Completed	VARCHAR
pft_mip_reason	If not, please comment	VARCHAR
pft_mip_reason_other	If other, please specify	VARCHAR
pft_pcf_attempt1	Best PCF attempt #1	FLOAT
pft_pcf_attempt2	Best PCF attempt #2	FLOAT
pft_pcf_attempt3	Best PCF attempt #3	FLOAT
pft_pcf_comment	Comments	VARCHAR
pft_pcf_completed	Test Completed	VARCHAR
pft_pcf_reason	If not, please comment	VARCHAR
pft_pcf_reason_other	If other, please specify	VARCHAR
pft_quest_validated	Questionnaire validated	VARCHAR
pft_snip_attempt1	SNIP #1 (cmH2O)	INT
pft_snip_attempt2	SNIP #2 (cmH2O)	INT

Identifiant	Libellé	Type
pft_snip_attempt3	SNIP #3 (cmH2O)	INT
pft_snip_comment	Comments	VARCHAR
pft_snip_completed	Test Completed	VARCHAR
pft_snip_reason	If not, please comment	VARCHAR
pft_snip_reason_other	If other, please specify	VARCHAR
qol_gendate	Date of questionnaire completion	DATE
qol_genperformed	PedsQL Generic Core Scale performed	VARCHAR
qol_genphysical	Physical health summary score	FLOAT
qol_genpsychosocial	Psychosocial health summary score	FLOAT
qol_genreason	Reason	VARCHAR
qol_gentotal	Total Scale score	FLOAT
qol_gentype	Form Type	VARCHAR
qol_hui_completed	Questionnaire validated	VARCHAR
qol_hui_date	Date of questionnaire completion	DATE
qol_hui_reason	Reason	VARCHAR
qol_hui2_cognition_multi	Score	FLOAT
qol_hui2_cognition_single	Score	FLOAT
qol_hui2_emotion_multi	Score	FLOAT
qol_hui2_emotion_single	Score	FLOAT
qol_hui2_mobility_multi	Score	FLOAT
qol_hui2_mobility_single	Score	FLOAT
qol_hui2_overallscore	HUI2 Overall Score	CALC
qol_hui2_pain_multi	Score	FLOAT
qol_hui2_pain_single	Score	FLOAT
qol_hui2_selfcare_multi	Score	FLOAT
qol_hui2_selfcare_single	Score	FLOAT
qol_hui2_sensation_multi	Score	FLOAT
qol_hui2_sensation_single	Score	FLOAT
qol_hui3_ambulation_multi	Score	FLOAT
qol_hui3_ambulation_single	Score	FLOAT
qol_hui3_cognition_multi	Score	FLOAT
qol_hui3_cognition_single	Score	FLOAT
qol_hui3_dexterity_multi	Score	FLOAT
qol_hui3_dexterity_single	Score	FLOAT
qol_hui3_emotion_multi	Score	FLOAT
qol_hui3_emotion_single	Score	FLOAT
qol_hui3_hearing_multi	Score	FLOAT
qol_hui3_hearing_single	Score	FLOAT
qol_hui3_overallscore	HUI3 Overall Score	CALC
qol_hui3_pain_multi	Score	FLOAT
qol_hui3_pain_single	Score	FLOAT
qol_hui3_speech_multi	Score	FLOAT
qol_hui3_speech_single	Score	FLOAT
qol_hui3_vision_multi	Score	FLOAT
qol_hui3_vision_single	Score	FLOAT

Identifiant	Libellé	Type
qol_neurocommu	Communication II score	FLOAT
qol_neurodate	Date of questionnaire completion	DATE
qol_neurodisease	Neuromuscular disease I score	FLOAT
qol_neurofamily	Family resources II score	FLOAT
qol_neuroperformed	PedsQL Neuromuscular Module performed	VARCHAR
qol_neuroreason	Reason	VARCHAR
qol_neurototal	Total score	FLOAT
qol_neurotype	Form Type	VARCHAR
qol_quest_validated	Questionnaire validated	VARCHAR
sma_acq_auto	Acquisition	VARCHAR
sma_acq_auto_age	Age (month)	INT
sma_acq_bring	Acquisition	VARCHAR
sma_acq_bring_age	Age (month)	INT
sma_acq_climb	Acquisition	VARCHAR
sma_acq_climb_age	Age (month)	INT
sma_acq_climbwithout	Acquisition	VARCHAR
sma_acq_climbwithout_age	Age (month)	INT
sma_acq_crawl	Acquisition	VARCHAR
sma_acq_crawl_age	Age (month)	INT
sma_acq_dress	Acquisition	VARCHAR
sma_acq_dress_age	Age (month)	INT
sma_acq_feed	Acquisition	VARCHAR
sma_acq_feed_age	Age (month)	INT
sma_acq_get	Acquisition	VARCHAR
sma_acq_get_age	Age (month)	INT
sma_acq_headctrl	Acquisition	VARCHAR
sma_acq_headctrl_age	Age (month)	INT
sma_acq_jump	Acquisition	VARCHAR
sma_acq_jump_age	Age (month)	INT
sma_acq_rollover	Acquisition	VARCHAR
sma_acq_rollover_age	Age (month)	INT
sma_acq_rollto	Aquisition	VARCHAR
sma_acq_rollto_age	Age (month)	INT
sma_acq_run	Acquisition	VARCHAR
sma_acq_run_age	Age (month)	INT
sma_acq_sit	Acquisition	VARCHAR
sma_acq_sit_age	Age (month)	INT
sma_acq_stand	Acquisition	VARCHAR
sma_acq_stand_age	Age (month)	INT
sma_acq_use	Acquisition	VARCHAR
sma_acq_use_age	Age (month)	INT
sma_acq_walk	Acquisition	VARCHAR
sma_acq_walk_age	Age (month)	INT
sma_apnoea	Sleep apnoea	VARCHAR
sma_assist	Assistive device	VARCHAR

Identifiant	Libellé	Type
sma_assist_a	Ankle Splint	VARCHAR
sma_assist_a_other	Other frequency (please specify)	VARCHAR
sma_assist_ca	Cane	VARCHAR
sma_assist_ca_other	Other frequency (please specify)	VARCHAR
sma_assist_co	Corset	VARCHAR
sma_assist_co_other	Other frequency (please specify)	VARCHAR
sma_assist_com	Communication Device	VARCHAR
sma_assist_com_other	Other frequency (please specify)	VARCHAR
sma_assist_com_sp	If Communication specify name	VARCHAR
sma_assist_f	Foot/Ankle... ?	VARCHAR
sma_assist_f_other	Other frequency (please specify)	VARCHAR
sma_assist_h	Hand/Wrist Splint	VARCHAR
sma_assist_h_other	Other frequency (please specify)	VARCHAR
sma_assist_lb	Lumbar belt	VARCHAR
sma_assist_lb_other	Other frequency (please specify)	VARCHAR
sma_assist_mw	Manual wheelchair	VARCHAR
sma_assist_mw_other	Other frequency (please specify)	VARCHAR
sma_assist_other	Other assistive device	VARCHAR
sma_assist_other_other	Other frequency (please specify)	VARCHAR
sma_assist_other_other2	Other frequency (please specify)	VARCHAR
sma_assist_other_other3	Other frequency (please specify)	VARCHAR
sma_assist_other_other4	Other frequency (please specify)	VARCHAR
sma_assist_other_other5	Other frequency (please specify)	VARCHAR
sma_assist_other_other6	Other frequency (please specify)	VARCHAR
sma_assist_other_sp	Other assistive device name	VARCHAR
sma_assist_other_sp2	Other assistive device name	VARCHAR
sma_assist_other_sp3	Other assistive device name	VARCHAR
sma_assist_other_sp4	Other assistive device name	VARCHAR
sma_assist_other_sp5	Other assistive device name	VARCHAR
sma_assist_other_sp6	Other assistive device name	VARCHAR
sma_assist_other2	Other assistive device - use frequency	VARCHAR
sma_assist_other3	Other assistive device - use frequency	VARCHAR
sma_assist_other4	Other assistive device - use frequency	VARCHAR
sma_assist_other5	Other assistive device - use frequency	VARCHAR
sma_assist_other6	Other assistive device - use frequency	VARCHAR
sma_assist_pw	Power wheelchair	VARCHAR
sma_assist_pw_other	Other frequency (please specify)	VARCHAR
sma_assist_s	Stander	VARCHAR
sma_assist_s_other	Other frequency (please specify)	VARCHAR
sma_assist_w	Walker	VARCHAR
sma_assist_w_other	Other frequency (please specify)	VARCHAR
sma_bipap	Non-invasive BiPAP (bi-level positive airway pressure)	VARCHAR
sma_bipap_fqcy	If currently using, how often	VARCHAR
sma_breathsup	Breathing support	VARCHAR
sma_breathsup_h	Number of hour of ventilation per day	FLOAT

Identifiant	Libellé	Type
sma_care	For teenagers and adults: care level	VARCHAR
sma_cpap	Non-invasive CPAP (continuous positive airway pressure)	VARCHAR
sma_cpap_fqcy	If currently using, how often	VARCHAR
sma_education	Education status	VARCHAR
sma_employment	For adults: Employment status	VARCHAR
sma_event	Number of family and community events missing due to the SMA	INT
sma_feed_dif	Difficulty for feeding	VARCHAR
sma_feed_dif_date	Date of last assessment	DATE
sma_feed_dif_other	If other	VARCHAR
sma_feed_dif_sucking	If difficulty for sucking	VARCHAR
sma_feed_dif_sucking_other	Please specify	VARCHAR
sma_feed_dif_type	Type of difficulty	VARCHAR
sma_feed_gast	Gastrostomy	VARCHAR
sma_feed_gast_end	Date of end	DATE
sma_feed_gast_intake	Percentage of calories intake	INT
sma_feed_gast_onset	If yes, Date of onset	DATE
sma_feed_naso	Naso-gastric tube	VARCHAR
sma_feed_naso_end	Date of end	DATE
sma_feed_naso_intake	Percentage of calories intake	INT
sma_feed_naso_onset	If yes, Date of onset	DATE
sma_feed_tb_current	Current need of a feeding tube	VARCHAR
sma_feed_tb_past	Past need of a feeding tube	VARCHAR
sma_iq	IQ total (formally evaluated)	VARCHAR
sma_iq_age	Age (Months)	INT
sma_iq_test	Method/Test	VARCHAR
sma_iq_value	Value	INT
sma_loss_auto	Loss	VARCHAR
sma_loss_auto_age	Age (month)	INT
sma_loss_bring	Loss	VARCHAR
sma_loss_bring_age	Age (month)	INT
sma_loss_climb	Loss	VARCHAR
sma_loss_climb_age	Age (month)	INT
sma_loss_climbwithout	Loss	VARCHAR
sma_loss_climbwithout_age	Age (month)	INT
sma_loss_crawl	Loss	VARCHAR
sma_loss_crawl_age	Age (month)	INT
sma_loss_dress	Loss	VARCHAR
sma_loss_dress_age	Age (month)	INT
sma_loss_feed	Loss	VARCHAR
sma_loss_feed_age	Age (month)	INT
sma_loss_get	Loss	VARCHAR
sma_loss_get_age	Age (month)	INT
sma_loss_headctrl	Loss	VARCHAR
sma_loss_headctrl_age	Age (month)	INT
sma_loss_jump	Loss	VARCHAR

Identifiant	Libellé	Type
sma_loss_jump_age	Age (month)	INT
sma_loss_rollo	Loss	VARCHAR
sma_loss_rollover_age	Age (month)	INT
sma_loss_rollto	Loss	VARCHAR
sma_loss_rollto_age	Age (month)	INT
sma_loss_run	Loss	VARCHAR
sma_loss_run_age	Age (month)	INT
sma_loss_sit	Loss	VARCHAR
sma_loss_sit_age	Age (month)	INT
sma_loss_stand	Loss	VARCHAR
sma_loss_stand_age	Age (month)	INT
sma_loss_use	Loss	VARCHAR
sma_loss_use_age	Age (month)	INT
sma_loss_walk	Loss	VARCHAR
sma_loss_walk_age	Age (month)	INT
sma_mental	Mental retardation	VARCHAR
sma_npv	NPV (negative pressure ventilation) aka Iron lung	VARCHAR
sma_npv_fqcy	If currently using, how often	VARCHAR
sma_ortho_f	Fracture	VARCHAR
sma_ortho_f_date1	Date	DATE
sma_ortho_f_date2	Date	DATE
sma_ortho_f_date3	Date	DATE
sma_ortho_f_date4	Date	DATE
sma_ortho_f_loca1	Localization	VARCHAR
sma_ortho_f_loca2	Localization	VARCHAR
sma_ortho_f_loca3	Localization	VARCHAR
sma_ortho_f_loca4	Localization	VARCHAR
sma_ortho_sc	Scolosis	VARCHAR
sma_ortho_sc_angle	Scoliosis angle	VARCHAR
sma_ortho_sc_arthro	Arthrodesis	VARCHAR
sma_ortho_sc_arthro_date	If yes, Date	DATE
sma_other	Other breathing support #1 (Use frequency)	VARCHAR
sma_other_frequency	If currently using, how often	VARCHAR
sma_other_frequency2	If currently using, how often	VARCHAR
sma_other_frequency3	If currently using, how often	VARCHAR
sma_other_frequency4	If currently using, how often	VARCHAR
sma_other_specify	If other, please specify	VARCHAR
sma_other_specify2	Other breathing support #2 name	VARCHAR
sma_other_specify3	Other breathing support #3 name	VARCHAR
sma_other_specify4	Other breathing support #4 name	VARCHAR
sma_other2	Other breathing support #2 (Use frequency)	VARCHAR
sma_other3	Other breathing support #3 (Use frequency)	VARCHAR
sma_other4	Other breathing support #4 (Use frequency)	VARCHAR
sma_quest_validated	Questionnaire validated	VARCHAR
sma_respinfection	Respiratory lower tract infection	VARCHAR

Identifiant	Libellé	Type
sma_respinfection_nb	Mean number per year	INT
sma_school	For children: School attendance	VARCHAR
sma_school_level	Level	VARCHAR
sma_schoolday	Number of school or work days missing due to the SMA	INT
sma_sp	Sip and Puff	VARCHAR
sma_sp_fqcy	If currently using, how often	VARCHAR
sma_vca	Vital capacity assessed	VARCHAR
sma_vca_date	Date of last assessment	DATE
sma_vca_value	Value	INT
sma_ventilator	Ventilator (with tracheostomy)	VARCHAR
sma_ventilator_fqcy	If currently using, how often	VARCHAR
sma_workday	Number of work days missing due to caring for a child with SMA	INT
str_completed	Test performed	VARCHAR
str_completed_reason	If not, please comment	VARCHAR
str_completed_reason_other	If other, please specify	VARCHAR
str_date	Assessment Date	DATE
str_dominant_hand	Dominant Hand	VARCHAR
str_evaluator	Evaluator	INT
calc_str_evaluator	Evaluator	VARCHAR
str_grip_comments	Comments	VARCHAR
str_grip_completed	Test performed	VARCHAR
str_grip_completed_reason	If not, please comment	VARCHAR
str_grip_completed_reason_other	If other, please specify	VARCHAR
str_grip_date	Assessment Date	DATE
str_grip_evaluator	Evaluator	INT
calc_str_grip_evaluator	Evaluator	VARCHAR
str_grip_trial1_result_dom	Dominant Hand Result trial #1	FLOAT
str_grip_trial1_result_nondom	Non Dominant Hand Result trial #1	FLOAT
str_grip_trial1_valid_dom	Valid or Invalid	VARCHAR
str_grip_trial1_valid_nondom	Valid or Invalid	VARCHAR
str_grip_trial2_result_dom	Dominant Hand Result trial #2	FLOAT
str_grip_trial2_result_nondom	Non Dominant Hand Result trial #2	FLOAT
str_grip_trial2_valid_dom	Valid or Invalid	VARCHAR
str_grip_trial2_valid_nondom	Valid or Invalid	VARCHAR
str_grip_trial3_result_dom	Dominant Hand Result trial #3	FLOAT
str_grip_trial3_result_nondom	Non Dominant Hand Result trial #3	FLOAT
str_grip_trial3_valid_dom	Valid or Invalid	VARCHAR
str_grip_trial3_valid_nondom	Valid or Invalid	VARCHAR
str_grip_trial4_result_dom	Dominant Hand Result trial #4	FLOAT
str_grip_trial4_result_nondom	Non Dominant Hand Result trial #4	FLOAT
str_grip_trial4_valid_dom	Valid or Invalid	VARCHAR
str_grip_trial4_valid_nondom	Valid or Invalid	VARCHAR
str_grip_trial5_result_dom	Dominant Hand Result trial #5	FLOAT
str_grip_trial5_result_nondom	Non Dominant Hand Result trial #5	FLOAT
str_grip_trial5_valid_dom	Valid or Invalid	VARCHAR

Identifiant	Libellé	Type
str_grip_trial5_valid_nondom	Valid or Invalid	VARCHAR
str_handcircumference_left	Left Hand Circumference	FLOAT
str_handcircumference_right	Right Hand Circumference	FLOAT
str_pinch_comments	Comments	VARCHAR
str_pinch_completed	Test performed	VARCHAR
str_pinch_completed_reason	If not, please comment	VARCHAR
str_pinch_completed_reason_other	If other, please specify	VARCHAR
str_pinch_date	Assessment Date	DATE
str_pinch_evaluator	Evaluator Name	INT
calc_str_pinch_evaluator	Evaluator	VARCHAR
str_pinch_trial1_result_dom	Dominant Hand Result trial #1	FLOAT
str_pinch_trial1_result_nondom	Non Dominant Hand Result trial #1	FLOAT
str_pinch_trial1_valid_dom	Valid or Invalid	VARCHAR
str_pinch_trial1_valid_nondom	Valid or Invalid	VARCHAR
str_pinch_trial2_result_dom	Dominant Hand Result trial #2	FLOAT
str_pinch_trial2_result_nondom	Non Dominant Hand Result trial #2	FLOAT
str_pinch_trial2_valid_dom	Valid or Invalid	VARCHAR
str_pinch_trial2_valid_nondom	Valid or Invalid	VARCHAR
str_pinch_trial3_result_dom	Dominant Hand Result trial #3	FLOAT
str_pinch_trial3_result_nondom	Non Dominant Hand Result trial #3	FLOAT
str_pinch_trial3_valid_dom	Valid or Invalid	VARCHAR
str_pinch_trial3_valid_nondom	Valid or Invalid	VARCHAR
str_pinch_trial4_result_dom	Dominant Hand Result trial #4	FLOAT
str_pinch_trial4_result_nondom	Non Dominant Hand Result trial #4	FLOAT
str_pinch_trial4_valid_dom	Valid or Invalid	VARCHAR
str_pinch_trial4_valid_nondom	Valid or Invalid	VARCHAR
str_pinch_trial5_result_dom	Dominant Hand Result trial #5	FLOAT
str_pinch_trial5_result_nondom	Non Dominant Hand Result trial #5	FLOAT
str_pinch_trial5_valid_dom	Valid or Invalid	VARCHAR
str_pinch_trial5_valid_nondom	Valid or Invalid	VARCHAR
str_quest_validated	Questionnaire validated	VARCHAR
time_10m_comment	Comments	VARCHAR
time_10m_completed	Test performed	VARCHAR
time_10m_completed_reason	If not, please comment	VARCHAR
time_10m_completed_reason_other	If other, please specify	VARCHAR
time_10m_graded_test	Graded Test	VARCHAR
time_10m_time_calc	Time to Run/Walk 10 m	CALC
time_6mwt_100_calc	Time to walk 100 m	CALC
time_6mwt_125_calc	Time to walk 125 m	CALC
time_6mwt_150_calc	Time to walk 150 m	CALC
time_6mwt_175_calc	Time to walk 175 m	CALC
time_6mwt_200_calc	Time to walk 200 m	CALC
time_6mwt_225_calc	Time to walk 225 m	CALC
time_6mwt_25_calc	Time to walk 25 m	CALC
time_6mwt_250_calc	Time to walk 250 m	CALC

Identifiant	Libellé	Type
time_6mwt_275_calc	Time to walk 275 m	CALC
time_6mwt_300_calc	Time to walk 300 m	CALC
time_6mwt_325_calc	Time to walk 325 m	CALC
time_6mwt_350_calc	Time to walk 350 m	CALC
time_6mwt_375_calc	Time to walk 375 m	CALC
time_6mwt_400_calc	Time to walk 400 m	CALC
time_6mwt_425_calc	Time to walk 425 m	CALC
time_6mwt_450_calc	Time to walk 450 m	CALC
time_6mwt_475_calc	Time to walk 475 m	CALC
time_6mwt_50_calc	Time to walk 50 m	CALC
time_6mwt_500_calc	Time to walk 500 m	CALC
time_6mwt_525_calc	Time to walk 525 m	CALC
time_6mwt_550_calc	Time to walk 550 m	CALC
time_6mwt_575_calc	Time to walk 575 m	CALC
time_6mwt_600_calc	Time to walk 600 m	CALC
time_6mwt_75_calc	Time to walk 75 m	CALC
time_6mwt_comments	Comments	VARCHAR
time_6mwt_completed	Test performed	VARCHAR
time_6mwt_completed_reason	If not, please comment	VARCHAR
time_6mwt_completed_reason_other	If other, please specify	VARCHAR
time_6mwt_dist_total	Total Distance (m)	INT
time_6mwt_fall1_calc	Fall #1 Time (sec)	CALC
time_6mwt_fall2_calc	Fall #2 Time (sec)	CALC
time_6mwt_fall3_calc	Fall #3 Time (sec)	CALC
time_6mwt_rest1_calc	Rest #1 Time (sec)	CALC
time_6mwt_rest2_calc	Rest #2 Time (sec)	CALC
time_6mwt_rest3_calc	Rest #3 Time (sec)	CALC
time_6mwt_valid	Test valid	VARCHAR
time_6mwt_valid_reason	If not, please comment	VARCHAR
time_climb_comment	Comments	VARCHAR
time_climb_completed	Test performed	VARCHAR
time_climb_completed_reason	If not, please comment	VARCHAR
time_climb_completed_reason_other	If other, please specify	VARCHAR
time_climb_graded_test	Graded Test	VARCHAR
time_climb_time_calc	Time to Climb 4 stairs	CALC
time_completed	Test performed	VARCHAR
time_completed_reason	If not, please comment	VARCHAR
time_completed_reason_other	If other, please specify	VARCHAR
time_cooperation	Patient Cooperation	VARCHAR
time_date	Assessment Date	DATE
time_descend_comment	Comments	VARCHAR
time_descend_completed	Test performed	VARCHAR
time_descend_completed_reason	If not, please comment	VARCHAR
time_descend_completed_reason_other	If other, please specify	VARCHAR
time_descend_graded_test	Graded Test	VARCHAR

Identifiant	Libellé	Type
time_descend_time_calc	Time to Descend 4 stairs	CALC
time_evaluator	Evaluator Name	INT
calc_time_evaluator	Evaluator	VARCHAR
time_quest_validated	Questionnaire validated	VARCHAR
time_rise_comment	Comments	VARCHAR
time_rise_completed	Test performed	VARCHAR
time_rise_completed_reason	If not, please comment	VARCHAR
time_rise_completed_reason_other	If other, please specify	VARCHAR
time_rise_graded_test	Graded Test	VARCHAR
time_rise_time_calc	Time to Rise From Floor	CALC
ttt_ae	Is the treatment indicated for AE	VARCHAR
ttt_ae_name	If yes, AE name	INT
calc_ttt_ae_name	AE Name	VARCHAR
ttt_dci	DCI	INT
calc_ttt_dci	Treatment DCI	VARCHAR
ttt_dose	Dose	VARCHAR
ttt_enddate	End Date	DATE
ttt_frequency	Frequency	VARCHAR
ttt_frequency_other	If other, please specify	VARCHAR
ttt_indication	Indication	INT
calc_ttt_indication	Treatment Indication	VARCHAR
ttt_name	Commercial name	INT
calc_ttt_name	Treatment Commercial Name	VARCHAR
ttt_ongoing	Ongoing	VARCHAR
ttt_route	Route	VARCHAR
ttt_route_other	If other, please specify	VARCHAR
ttt_startdate	Start Date	DATE
ttt_unit	Unit	VARCHAR
v_agerange	Age Range	VARCHAR
v_amb	Ambulant	VARCHAR
v_date	Date of the visit	DATE
v_physioend	Time Finished	TIME
v_physiostart	Time Started	TIME
v_type	Type of visit	VARCHAR
