# Supplementary material for: Prospective and longitudinal natural history study of patients with Type 2 and 3 spinal muscular atrophy: Baseline data NatHis-SMA study
Source: PLoS One. 2018 Jul 26;13(7):e0201004. doi: 10.1371/journal.pone.0201004 (PMC6062049; doi:10.1371/journal.pone.0201004)
Supplement: S1 Table — Abbreviations: bpm: breath per minute, BPAP: Bi-level positive airway pressure, CPAP: Continuous Positive Airway Pressure, IPV: Intrapulmonary Percussive Ventilation, NPV: Negative Pressure Ventilation, IPPV: Intermittent Positive Pressure Ventilation, AFO: Ankle and Foot Orthosis; Values are median (IQR) and overall population size is n = 81 unless otherwise indicated; * 0.001 < p ≤ 0.05; ** p ≤ 0.001; □ Application conditions of the Chi-square test not fully verified (theoretical effectives ≤ 5, too small effectives); a, b, c Subscript letters represent Post-hoc tests results. In a row, a same subscript letter indicates a subset of categories (non-sitter SMA type 2, sitter SMA type 2, non-ambulant SMA type 3 and ambulant SMA type 3) which column proportions do not differ significantly from each other at level 0.05. (DOCX) [file pone.0201004.s004.docx]

|  | | | | | | | | | **SMA type 2** | | **SMA type 3** | | **Overall (n=81)** |
| --- | --- | --- | --- | --- | --- | --- | --- | --- | --- | --- | --- | --- | --- |
|  | | | | | | | | | **Non-sitter (n=19)** | **Sitter (n=34)** | **Non-Ambulant (n=9)** | **Ambulant (n=19)** |  |
| **Site (n)** | | | | | | | | |  |  |  |  |  |
|  | | | | | | | | *Paris* | *2* | *10* | *4* | *3* | *19* |
|  | | | | | | | | *Liège* | *5* | *5* | *0* | *4* | *14* |
|  | | | | | | | | *Nantes* | *5* | *4* | *0* | *1* | *10* |
|  | | | | | | | | *Toulouse* | *2* | *5* | *1* | *2* | *10* |
|  | | | | | | | | *Lyon* | *0* | *3* | *1* | *3* | *7* |
|  | | | | | | | | *Lille* | *3* | *2* | *1* | *0* | *6* |
|  | | | | | | | | *Leuven* | *1* | *2* | *1* | *2* | *6* |
|  | | | | | | | | *Strasbourg* | *0* | *2* | *0* | *3* | *5* |
|  | | | | | | | | *Essen* | *1* | *1* | *1* | *1* | *4* |
| **Male (n)** | | | | | | | | | *7* | *14* | *6* | *10* | *37* |
| **Other family member affected (%)** | | | | | | | | | *1* | *3* | *2* | *4* | *10* |
| **Medical history (perinatal period)** | | | | | | | | |  |  |  |  |  |
|  | | | *Birth (week of amenorrhea; n=77)* | | | | | | *40 (38-40)* | *39 (39-41)* | *40 (38-41)* | *40 (39-40)* | *40 (39-40)* |
|  | | | *Birth weight (g; n=80) ** | | | | | | *3040* _a_  *(2560-3290)* | *3445* _b, c_  *(3200-3894)* | *3250* _a, c_  *(2050-3460)* | *3360* _a, c_  *(2960-3676)* | *3300*  *(3021-3635)* |
|  | | | *Birth length (cm; n=77)** | | | | | | *48* _a_ *(48-49)* | *50* _b, c_ *(49-52)* | *49* _a, c_ *(47-53)* | *50* _a, c_ *(48-52)* | *48 (48-51)* |
|  | | | *Head circumference (cm; n=65)* | | | | | | *34 (33-35)* | *35 (34-36)* | *35 (33-36)* | *34 (32-35)* | *33 (33-36)* |
| **Medical history (events total number)** | | | | | | | | |  |  |  |  |  |
|  | | | | | *Eye/Ear/Nose/Throat* | | | | *1 (0-2)* | *0 (0-2)* | *0 (0-2)* | *0 (0-1)* | *0 (0-2)* |
|  | | | | | *Neurologic and Psychiatric* | | | | *0 (0-1)* | *0 (0-1)* | *0 (0-1)* | *1 (0-2)* | *0 (0-1)* |
|  | | | | | *Pulmonary *** | | | | *3* _a_ *(1-5)* | *1* _a, b_ *(0-3)* | *1* _a, b_ *(0-2)* | *0* _b, c_ *(0-1)* | *1 (0-3)* |
|  | | | | | *Cardiovascular and Blood* | | | | *0 (0-0)* | *0 (0-0)* | *0 (-)* | *0 (0-0)* | *0 (0-0)* |
|  | | | | | *Musculoskeletal *** | | | | *3* _a_ *(2-3)* | *1* _b_ *(0-3)* | *1* _a, b_ *(0-2)* | *1* _b_ *(1-2)* | *1 (1-3)* |
|  | | | | | *Hepatic-Gastrointestinal* | | | | *0 (0-0)* | *0 (0-0)* | *0 (0-0)* | *0 (0-0)* | *0 (0-0)* |
|  | | | | | *Metabolic* | | | | *0 (0-0)* | *0 (0-0)* | *0 (0-0)* | *0 (0-0)* | *0 (0-0)* |
|  | | | | | *Genitourinary ** | | | | *0 (0-1)* | *0 (0-0)* | *0 (0-1)* | *0 (0-0)* | *0 (0-1)* |
|  | | | | | *Skin* | | | | *0 (0-1)* | *0 (0-0)* | *0 (0-1)* | *0 (0-1)* | *0 (0-1)* |
| **Heart rate (bpm; n=79) **** | | | | | | | | | *99* _a, b_ *(90-115)* | *115* _a_ *(99-122)* | *79* _c_ *(75-84)* | *93* _b, c_ *(72-115)* | *100 (84-117) ^Δ^* |
| **Systolic blood pressure (mmHg; n=78)** | | | | | | | | | *108 (96-124)* | *103 (95-112)* | *111 (107-127)* | *106 (98-115)* | *106 (98-115) ^Δ^* |
| **Diastolic blood pressure (mmHg; n=78)** | | | | | | | | | *69 (58-85)* | *67 (60-73)* | *70 (66-72)* | *62 (57-69)* | *66 (60-72) ^Δ^* |
| **Organs and systems (abnormal, n)** | | | | | | | | |  |  |  |  |  |
|  | | | | *Eye/Ear/Nose/Throat* | | | | | *2* | *3* | *1* | *0* | *6* |
|  | | | | *Neurologic and Psychiatric* | | | | | *14* | *27* | *5* | *18* | *64* |
|  | | | | *Pulmonary ** ^□^ | | | | | *5* | *3* | *0* | *0* | *8* |
|  | | | | *Cardiovascular and Blood* | | | | | *0* | *1* | *0* | *1* | *2* |
|  | | | | *Musculoskeletal* | | | | | *18* | *33* | *9* | *19* | *79* |
|  | | | | *Hepatic-Gastrointestinal* | | | | | *2* | *3* | *0* | *0* | *5* |
|  | | | | *Metabolic ** ^□^ | | | | | *4* _a_ | *0* _b_ | *0* _a, b_ | *0* _a, b_ | *4* |
|  | | | | *Genitourinary* | | | | | *2* | *2* | *0* | *0* | *4* |
|  | | | | *Skin* | | | | | *6* | *3* | *1* | *3* | *13* |
| **Ventilation details (n)** | | | | | | | | |  |  |  |  |  |
|  |  | | | | | Non Invasive Ventilation | | |  |  |  |  |  |
|  |  | | | | | *CPAP* | | | *4* | *3* | *0* | *0* | *7* |
|  |  | | | | | *BPAP* | | | *10* | *10* | *0* | *0* | *20* |
|  |  | | | | | *IPV* | | | *5* | *5* | *0* | *0* | *10* |
|  |  | | | | | *NPV* | | | *1* | *0* | *0* | *0* | *1* |
|  |  | | | | | *Sip and puff* | | | *1* | *0* | *0* | *0* | *1* |
|  |  | | | | | *IPPV* | | | *8* | *11* | *1* | *1* | *21* |
|  |  | | | | | *Cough assist* | | | *8* | *8* | *0* | *0* | *16* |
|  |  | | | | | Invasive Ventilation | | |  |  |  |  |  |
|  |  | | | | | *Tracheostomy* | | | *0* | *1* | *0* | *0* | *1* |
| **Feeding difficulties details (n)** | | | | | | | | |  |  |  |  |  |
|  | | | | | | | *Sucking* | | *2* | *0* | *0* | *0* | *2* |
|  | | | | | | | *Swallowing* | | *8* | *6* | *1* | *0* | *15* |
|  | | | | | | | *Chewing* | | *10* | *7* | *0* | *0* | *17* |
|  | | | | | | | *Reflux/vomiting* | | *2* | *5* | *0* | *0* | *7* |
| **Assistive devices details (n)** | | | | | | | | |  |  |  |  |  |
|  | | *AFO, Hand & ankle splint ** ^□^ | | | | | | | *18* _a_ | *27* _a, b_ | *8* _a, b_ | *6* _b_ | *59* |
|  | | *Power wheelchair *** ^□^ | | | | | | | *17* _a_ | *25* _a_ | *7* _a_ | *2* _b_ | *51* |
|  | | *Manual wheelchair *** ^□^ | | | | | | | *11* _a, b_ | *24* _b_ | *9* _b_ | *3* _a_ | *47* |
|  | | *Stander *** ^□^ | | | | | | | *14* _a_ | *25* _a_ | *3* _a, b_ | *1* _b_ | *43* |
|  | | *Corset, lumbar belt *** ^□^ | | | | | | | *19* _a_ | *18* _b_ | *2* _b, c_ | *1* _c_ | *40* |
|  | | *Walker, cane *** ^□^ | | | | | | | *0* _a_ | *7* _a, b_ | *6* _b_ | *2* _a, b_ | *15* |
|  | | *Medicalized bed, positioning mattress* | | | | | | | *5* | *3* | *3* | *0* | *11* |
|  | | *Orthopaedic soles/shoes, heel cup ** ^□^ | | | | | | | *1* _a, b_ | *1* _b_ | *2* _a, b_ | *5* _a_ | *9* |
|  | | *Communication device* | | | | | | | *1* | *1* | *0* | *2* | *4* |
| **Absence of cognitive impairment (n)** | | | | | | | | | *19* | *34* | *9* | *19* | *81* |
| **School attendance (6-18 years old; n)** | | | | | | | | | *n=11* | *n=8* | *n=2* | *n=5* | *n=26* |
|  | | | | | | | | | *11* | *8* | *2* | *5* | *26* |
| **School level (18-30 years old, n)** | | | | | | | | | *n=4* | *n=1* | *n=7* | *n=5* | *n=17* |
|  | | | | | | | | *High school* | *2* | *0* | *3* | *1* | *6* |
|  | | | | | | | | *College* | *1* | *1* | *3* | *0* | *5* |
|  | | | | | | | | *Post Graduate* | *1* | *0* | *1* | *4* | *6* |
| **Employment status (18-30 years old; n)** | | | | | | | | | *n=4* | *n=1* | *n=6* | *n=5* | *n=16* |
|  | | | | | | | | *Full time* | *1* | *0* | *0* | *1* | *12* |
|  | | | | | | | | *Part time* | *0* | *0* | *1* | *0* | *6* |
|  | | | | | | | | *Student* | *2* | *1* | *3* | *3* | *53* |
|  | | | | | | | | *Unemployed* | *1* | *0* | *2* | *1* | *24* |
