# Supplementary material for: Prospective and longitudinal natural history study of patients with Type 2 and 3 spinal muscular atrophy: Baseline data NatHis-SMA study
Source: PLoS One. 2018 Jul 26;13(7):e0201004. doi: 10.1371/journal.pone.0201004 (PMC6062049; doi:10.1371/journal.pone.0201004)
Supplement: S2 Table — Values are effectives per motor skill; * 0.001 < p ≤ 0.05; ** p ≤ 0.001; □ Application conditions of the Chi-square test not fully verified (theoretical effectives ≤ 5, too small effectives); a, b, c Subscript letters represent Post-hoc tests results. In a row, a same subscript letter indicates a subset of categories (non-sitter SMA type 2, sitter SMA type 2, non-ambulant SMA type 3 and ambulant SMA type 3) which do not differ significantly from each other at level 0.05. (DOCX) [file pone.0201004.s005.docx]

|  | | **Acquisition** | | | | | **Loss** | | | | | |
| --- | --- | --- | --- | --- | --- | --- | --- | --- | --- | --- | --- | --- |
|  |  | **SMA type 2** | | **SMA type 3** | |  | **SMA type 2** | | **SMA type 3** | |  |  |
|  |  | **Non-Sitters** | **Sitters** | **Non-Ambulant** | **Ambulant** | **Overall** | **Non-Sitters** | **Sitters** | **Non-Ambulant** | **Ambulant** | **Overall** |  |
| **General motor skills** | **Head control** | 19 | 34 | 9 | 19 | 81 | 5 | 1 | 0 | 0 | 6 *** ^□^** |  |
|  | **Sit independently when placed** | 16 | 33 | 9 | 19 | 77 | 9 _a_ | 4 _b_ | 2 _a, b_ | 0 _b_ | 15 **** ^□^** |  |
|  | **Roll to one side** | 12 _a_ | 28 _a, b_ | 9 _a, b_ | 19 _b_ | 68 *** ^□^** | 11 _a_ | 7 _b_ | 0 _b_ | 0 _b_ | 18 **** ^□^** |  |
|  | **Roll over completely** | 9 _a_ | 22 _a_ | 9 _a, b_ | 19 _b_ | 59 **** ^□^** | 9 _a_ | 10 _b_ | 1 _b, c_ | 0 _c_ | 20 **** ^□^** |  |
|  | **Crawl** | 4 _a_ | 11 _a_ | 9 _b_ | 18 _b_ | 42 **** ^□^** | 4 _a_ | 9 _a_ | 4 _a, b_ | 1 _b_ | 18 **** ^□^** |  |
|  | **Get into sitting position** | 5 _a_ | 9 _a_ | 9 _b_ | 19 _b_ | 42 **** ^□^** | 4 _a_ | 4 _a_ | 1 _a, b_ | 0 _b_ | 9 **** ^□^** |  |
|  | **Stand up** | 4 _a_ | 8 _a_ | 8 _b_ | 19 _b_ | 39 **** ^□^** | 4 _a_ | 7 _a_ | 7 _a_ | 1 _b_ | 19 **** ^□^** |  |
|  | **Walk with help** | 4 _a_ | 7 _a_ | 9 _b_ | 19 _b_ | 39 **** ^□^** | 4 _a_ | 5 _a_ | 7 _a_ | 0 _b_ | 16 **** ^□^** |  |
|  | **Climb stairs with banister** | 1 _a_ | 1 _a_ | 5 _b_ | 17 _b_ | 24  **** ^□^** | 1 _a_ | 1 _a_ | 5 _a_ | 1 _b_ | 8 **** ^□^** |  |
|  | **Walk without help** | 0 _a_ | 0 _a_ | 9 _b_ | 19 _b_ | 28 **** ^□^** | - | - | 9 _a_ | 1 _b_ | 10 **** ^□^** |  |
|  | **Run** | 0 _a_ | 0 _a_ | 3 _b_ | 9 _b_ | 12 **** ^□^** | - | - | 3 _a_ | 3 _b_ | 6 |  |
|  | **Jump** | 0 _a_ | 0 _a_ | 2 _a, c_ | 9 _c_ | 11 **** ^□^** | - | - | 2 _a_ | 2 _b_ | 4 |  |
|  | **Climb stairs without banister** | 0 _a, b_ | 0 _b_ | 2 _a, c_ | 6 _c_ | 8 **** ^□^** | - | - | 2 | 4 | 6 |  |
| **Fine motor skills** | **Bring hands to mouth** | 17 | 34 | 9 | 19 | 79 | 0 | 2 | 0 | 0 | 2 |  |
|  | **Feed with finger** | 17 | 34 | 9 | 19 | 79 | 1 | 2 | 0 | 0 | 3 |  |
|  | **Eat with ustensils** | 16 | 34 | 9 | 19 | 78 *** ^□^** | 1 | 2 | 0 | 0 | 3 |  |
|  | **Self-dress** | 1 _a_ | 1 _a_ | 9 _b_ | 15 _b_ | 26 **** ^□^** | 1 _a_ | 1 _a_ | 2 _a, b_ | 0 _b_ | 4 *** ^□^** |  |
