# Supplementary figures and images for: Prospective and longitudinal natural history study of patients with Type 2 and 3 spinal muscular atrophy: Baseline data NatHis-SMA study
Source: PLoS One. 2018 Jul 26;13(7):e0201004. doi: 10.1371/journal.pone.0201004 (PMC6062049; doi:10.1371/journal.pone.0201004)

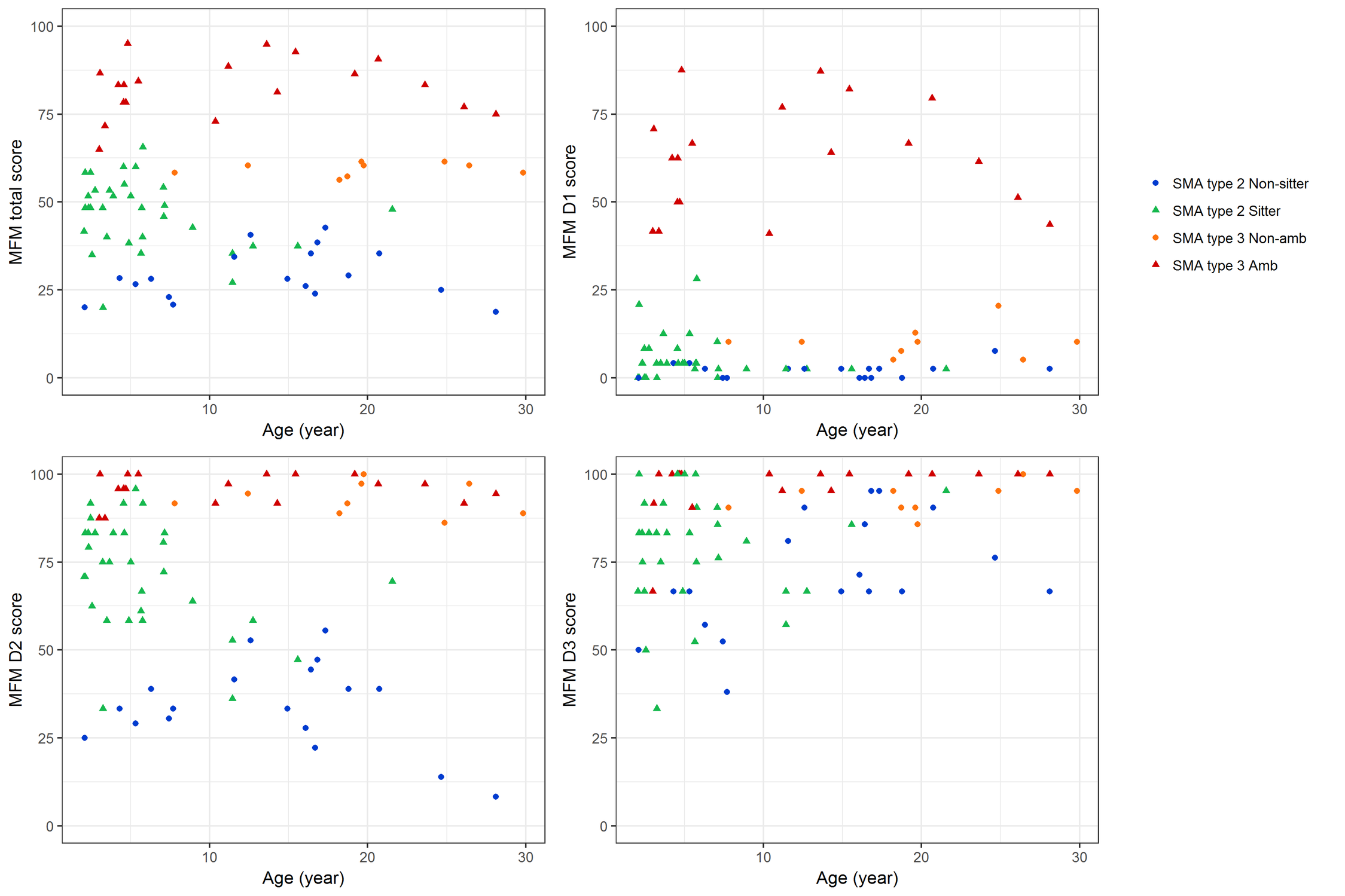

Supplement: S1 Fig — (TIF) [file pone.0201004.s006.tif]
